# Nucleophilicity of Glutathione: A Link to Michael Acceptor Reactivities

**DOI:** 10.1002/anie.201909803

**Published:** 2019-10-31

**Authors:** Robert J. Mayer, Armin R. Ofial

**Affiliations:** ^1^ Department Chemie Ludwig-Maximilians-Universität München Butenandtstraße 5–13 81377 München Germany

**Keywords:** electrophilicity, kinetics, Michael addition, nucleophilicity, thiols

## Abstract

Deprotonated glutathione is among the most potent biological nucleophiles and plays an important physiological role in cellular detoxification by forming covalent conjugates with Michael acceptors. The electrophilicity *E* of various Michael acceptors was characterized recently according to the Patz–Mayr relation lg *k*_2_=*s*_N_(*N*+*E*). We now determined the nucleophilic reactivity (*N*, *s*_N_) of glutathione (GSH) in aqueous solution at 20 °C to connect published GSH reactivities (*k*_GSH_) with Mayr's electrophilicity scale (*E*). In this way, electrophilicities *E* of more than 70 Michael acceptors could be estimated, which can now be used to systematically predict novel reactions with the multitude of nucleophiles whose nucleophilicity parameters *N*/*s*_N_ are known.

## Graphical abstract

**Connected**: The nucleophilicity parameters *N* and *s*_N_ of glutathione (GSH) were determined in aqueous solution at 20 °C to link published GSH reactivities (lg *k*_GSH_) with Mayr's electrophilicity scale (*E*). The electrophilic reactivities of Michael acceptors determined in kinetic chemoassays can now be compared with reactivities of neutral and cationic electrophiles characterized on the *E* scale.

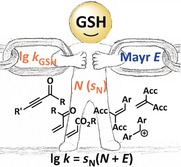

Michael acceptors are often used and versatile electrophiles in organic synthesis. Their capability to form conjugates with peptides bearing nucleophilic groups, such as the thiol of the tripeptide glutathione (GSH), supplies them with a broad spectrum of bioactivity. To assess the toxicity of xenobiotic Michael acceptors^1^, ^2^ as well as to estimate their potential as biological tools^3^, ^4^, ^5^ or covalent drugs,^2^, ^6^ the kinetics of non‐enzymatic GSH thiol–Michael additions have been broadly investigated under physiological conditions.^2^, 6l, 7, 8, 9 The corresponding second‐order rate constants, *k*_GSH_, provide the experimental basis for structure–activity relationships that comprise, for example, α,β‐unsaturated aldehydes, ketones, and esters.^2^, 6g

The kinetics of Michael additions of carbon‐centered reference nucleophiles were extensively studied by Mayr and co‐workers, who used Equation (1) to establish an ordering system for the electrophilic reactivity of structurally diverse Michael acceptors.^10^ Equation (1) is a linear free energy relationship that calculates solvent‐independent electrophilicity parameters *E* from experimentally determined second‐order rate constants *k*_2_ for the reactions of electrophiles with nucleophiles of known nucleophilic reactivities *N* and susceptibilities *s*_N_ (in a certain solvent).^11^

(1) $$\mathrm{lg}\;k{}_{2}\left(20\;{}^{\circ }C\right)=s{}_{N}\left(N+E\right)$$

Kamiya, Urano, and co‐workers recently demonstrated that Mayr's electrophilicities *E* provide a useful guide for the rational design of real‐time dynamic GSH fluorescent probes.4a We therefore set out to interconnect electrophilicity rankings for Michael acceptors originating from rate constants of their reactions with GSH2d, 7, 8, 9 with those relying on Mayr electrophilicities *E*.^10^, ^12^ To achieve the contact between both reactivity scales we determined the Mayr nucleophilicity of GSH in aqueous solution from the kinetics of its reactions with the reference electrophiles **E1**–**E17** (Figure 1). As a consequence, the mutual interconversion of known electrophilic reactivities lg *k*_GSH_ and *E* becomes possible.

**Figure 1:**
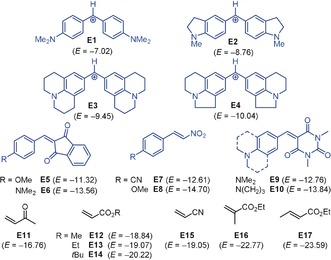
Reference electrophiles **E1**–**E17** used in this study (counterion for benzhydrylium ions **E1**–**E4**: BF_4_^−^; electrophilicities *E* from Refs. 10, 11b, 12).

Dropwise addition of a deeply blue acetonitrile solution of the electrophile **E1** 13 to a neutral, aqueous solution of GSH led to fading of the blue color within seconds because of the rapid formation of the colorless S‐benzhydrylated adduct **GS‐E1**⋅HBF_4_ (Scheme 1).

**Scheme 1:**
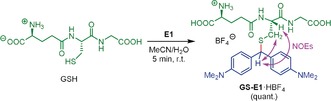
Adduct formation between GSH and the benzhydrylium tetrafluoroborate **E1** in neutral, aqueous solution. NOESY experiments indicated a benzhydrylated Cys moiety in **GS‐E1**.

Thiolate attack at the cationic center of **E1** also occurred, though significantly faster, when **E1** was added to an alkaline, aqueous solution of GSH to yield **GS‐E1**. Competing attack of the γ‐glutamyl NH_2_ group of GSH at **E1** was not detected (see the Supporting Information), in agreement with the rate ratio of >100:1 for the S^−^/NH_2_ attack of GSH at acrylonitrile (pH 8.1, 30 °C) determined by Friedman and co‐workers.7a, 14 Accordingly, the analogous reaction of **E1** with *N*‐acetylcysteine (AcCys), which is devoid of a reactive NH_2_ group, gave rise to S‐benzhydrylated AcCys (see the Supporting Information).

Owing to the similar acidities of the thiol and the ammonium group of GSH(NH_3_^+^/SH), they are deprotonated simultaneously in the pH range 7 to 12, producing two reactive thiolate species, that is, GSH(NH_3_^+^/S^−^) and GSH(NH_2_/S^−^). Depending on the pH value, variable fractions of the individual thiolates are present in the aqueous solutions, and microscopic ionization constants are needed to describe the acid–base equilibria7a, 15 (see the Supporting Information).

The rates of GSH adduct formation with reference electrophiles **E2**–**E10** were investigated at pH 12 in aqueous solution (20 °C). At this pH, the thiol groups of GSH15a–15c and AcCys,15d respectively, are almost quantitatively (>99 %) deprotonated to the corresponding thiolates RS^−^ [that is, GSH(NH_2_/S^−^) from GSH]. The thiolates RS^−^ add directly to the cationic reference electrophiles **E2**–**E4** or undergo conjugate additions to the neutral Michael acceptors **E5**–**E10** (Scheme 2).

**Scheme 2:**
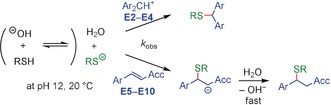
Reactions of cationic and neutral electrophiles **E** with thiolate ions generated from GSH or AcCys in alkaline, aqueous solutions.

The kinetics of the covalent bond formation between the deprotonated GSH (or AcCys) and the electrophiles **E** were monitored by following the decay of the UV/Vis absorbance of the colored cationic or neutral electrophiles by using the stopped‐flow technique (see the Supporting Information). With thiolate ions at at least tenfold higher concentrations than their electrophilic reaction partners (pseudo‐first‐order conditions), we observed rapid mono‐exponential decays of the electrophile concentrations. First‐order rate constants *k*_obs_ (s^−1^) were obtained by least‐squares fitting of the single‐exponential function *A_t_*=*A*_0_e${}^{-k{}_{\mathrm{obs}}t}$ +*C* to the time‐dependent absorbance (Figure 2 a).

**Figure 2:**
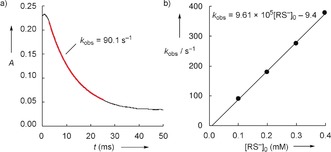
a) Decay of the absorbance *A* (at 383 nm) in the reaction of GSH (*c=*1.00×10^−4^ m) with **E5** (*c=*1.03×10^−5^ m) at 20 °C (aqueous solution, pH 12). The fitted mono‐exponential function is depicted in red. b) The slope of the linear correlation of the first‐order rate constant *k*_obs_ with the initial concentration of the GSH thiolate [RS^−^]_0_ was used to derive the second‐order rate constant *k*_2_ for the attack of the GSH thiolate at the Michael acceptor **E5**.

Table 1 lists the second‐order rate constants *k*_2_ (m ^−1^ s^−1^) for the attack of thiolate ions generated from GSH and AcCys, respectively, at the reference electrophiles **E2**–**E10**, which (according to *k*_obs_=*k*_2_[RS^−^]_0_) were obtained as the slopes of the linear correlations of *k*_obs_ with the thiolate concentrations (as exemplified for the reaction of GSH with **E5** in Figure 2 b and for all other combinations in the Supporting Information). Table 1 also comprises rate constants for the reactions of cysteine with benzhydrylium ions **E2**–**E4**, which were determined by Brotzel and Mayr.^16^ For each of the electrophiles **E2**–**E10**, the reactivities towards GSH and the less functionalized AcCys (or Cys) agree within a factor of 2.5. We conclude from this comparison that exclusively the thiolate reactivity of GSH(NH_2_/S^−^) was detected in our kinetic measurements.

**Table 1:** Second‐order rate constants *k*_2_ for the reactions of **E2–E10** with thiolates generated from glutathione (GSH), *N*‐acetylcysteine (AcCys), and cysteine (Cys) by deprotonation at pH 12 in aqueous solution at 20 °C.

Electrophile		*k*_2_ (m ^−1^ s^−1^)	
	GSH	AcCys	Cys^[a]^
**E2**	2.70×10^6^	1.47×10^6^	1.29×10^6^
**E3**	1.37×10^6^	5.57×10^5^	6.41×10^5^
**E4**	6.60×10^5^	3.47×10^5^	3.79×10^5^
**E5**	9.61×10^5^	5.21×10^5^	–
**E6**	7.60×10^4^	–	–
**E7**	1.46×10^5^	–	–
**E8**	3.62×10^4^	3.14×10^4^	–
**E9**	2.58×10^5^	1.96×10^5^	–
**E10**	5.31×10^4^	4.12×10^4^	–

[a] With *k*_2_ from Ref. 16.

Kinetic assays used to investigate GSH reactivity towards electrophilic targets, such as Michael acceptors, are performed in buffered solutions at physiological pH, that is, usually at pH 7.4. In the range pH 7 to 8, only GSH(NH_3_^+^/S^−^) is formed as a reactive thiolate. Although weakly populated (1–10 % of [GSH]_0_),15a–15c this fraction *F* of nucleophilic thiolate ions accounts for the observed GSH reactivity towards electrophiles under physiological conditions. Equation (2) allows one to convert the second‐order rate constants *k*_GSH_ into second‐order rate constants *k*_2_ for the corresponding GSH(NH_3_^+^/S^−^) thiolate reactions.

(2) $$k{}_{2}\;\left(M{}^{-1}\;s{}^{-1}\right)=k{}_{\mathrm{GSH}}/F$$

Reported pH‐dependent second‐order rate constants *k*_GSH_ 2d, 7 for the reactions of GSH with the Michael acceptors **E11**–**E17**, whose Mayr electrophilicities *E* are known, are compiled in Table 2 along with the second‐order rate constants *k*_2_ for the corresponding thiolate reactivity [from Eq. (2)].

**Table 2:** Second‐order rate constants *k*_GSH_ as reported for the reactions of GSH with electrophiles **E11–E17** at a certain pH value in aqueous, buffered solutions and second‐order rate constants *k*_2_ [calculated by using Eq. (2)] for the reactions of the GSH(NH_3_^+^/S^−^) thiolate.

Electrophile	*E* ^[a]^	*k*_GSH_ (m ^−1^ s^−1^)	*k*_2_ (m ^−1^ s^−1^)
**E11**	−16.76	3.19×10^1^ (pH 7.4)^[b]^	1.14×10^3^
**E12**	−18.84	1.90×10^−1^ (pH 7.4)^[c]^	6.79
**E13**	−19.07	1.77×10^−1^ (pH 7.4)^[c]^	6.31
**E14**	−20.22	4.17×10^−2^ (pH 7.4)^[c]^	1.49
**E15**	−19.05	1.73×10^−1^ (pH 8.1)^[d]^	1.36
**E16**	−22.77	9.67×10^−4^ (pH 7.4)^[c]^	3.45×10^−2^
**E17**	−23.59	3.10×10^−3^ (pH 7.4)^[c]^	1.11×10^−1^

[a] Mayr electrophilicities *E* from Ref. 10. [b] At 20 °C, from Ref. 7b; *F*=0.0280. [c] At 25 °C, from Ref. 2d; *F*=0.0280. [d] At 30 °C, from Ref. 7a; *F*=0.127.

As shown in Table 1, the relative electrophilic reactivities *E* of **E2**–**E10** also hold for their reactions towards the GSH(NH_2_/S^−^) thiolate in aqueous solution. Friedman showed that protonation at the remote γ‐glutamyl NH_2_ group in GSH reduces the thiolate reactivity by a factor of 27a (see the Supporting Information for evidence that this factor is also appropriate in our studies). By applying Friedman's factor to convert the *k*_2_ values from Table 1, a linear correlation over 15 orders of magnitude of electrophilic reactivity results from the combined set of second‐order rate constants for reactions of the thiolate GSH(NH_3_^+^/S^−^) with **E2**–**E17** when correlated with their *E* descriptors (Figure 3).^17^ The slope of the linear correlation in Figure 3 corresponds to *s*_N_ (=0.56) for the thiolate GSH(NH_3_^+^/S^−^) in water, and from the intercept with the abscissa, a nucleophilicity of *N*=20.97 is obtained.

**Figure 3:**
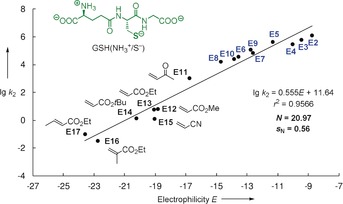
Determination of the nucleophilicity of GSH(NH_3_^+^/S^−^) from the linear plot of lg *k*_2_ for its reactions with the electrophiles **E2**–**E17** (with 0.5 *k*_2_ from Table 1 and *k*_2_ from Table 2) versus the electrophilicity parameters *E*.

Conversely, the thus determined GSH nucleophilicity (*N*, *s*_N_) allows one to estimate the electrophilicity for Michael acceptors whose reactivity had thus far only been investigated in kinetic GSH assays.2d, 7, 8, 9 After converting the reported rate constants *k*_GSH_ into *k*_2_ [Eq. (2)], Equation (1) was used to assess *E* for various enones, ynones, enals, ynals, and further α,β‐unsaturated carbonyl compounds **M1**–**M73** (see Table S4 in the Supporting Information). For example, applying *k*_GSH_=8.12 m ^−1^ s^−1^ (pH 7.4, 25 °C, from Ref. 2d) for 2‐octynal (**M28**) in Equation (2) yields *k*_2_=290 m ^−1^ s^−1^. After inserting *k*_2_ into Equation (1), the electrophilic reactivity of 2‐octynal is rated with *E*=−16.5. Analogously, electrophilicities *E* of another 72 Michael acceptors within the range of −14.4≤*E*≤−25.3 were estimated.^18^ Available rate constants furthermore create opportunities to integrate the recently developed dynamic GSH fluorescent probes **M74**–**M82** into Mayr's electrophilicity scale (Scheme 3).3a, 4b, 19

**Scheme 3:**
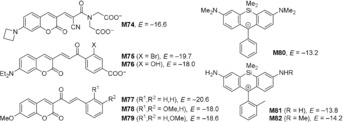
Estimated electrophilicities *E* of the GSH fluorescent probes **M74**–**M82** (see Table S7 in the Supporting Information for details).

It was proposed that quantum‐chemically calculated methyl anion affinities Δ*G*_MA_ (Figure 4 a) could be used to predict trends in the electrophilic reactivities *E* of Michael acceptors.^10^ The *E* vs. Δ*G*_MA_ correlation for the Michael acceptors with *E* derived from reactions with carbon‐centered nucleophiles was reported to be linear (*r* ^2^=0.8857, *n=*44, black dots in Figure 4 b).^10^ The *E* parameters estimated in this work solely from a single rate constant in kinetic GSH assays cannot be expected to be as accurate as classical electrophilicities *E*,^18^ which are based on evaluating a set of kinetics for C−C bond‐forming reactions with carbon‐centered reference nucleophiles.^10^ To assess the general consistency, however, we included calculated Δ*G*_MA_ values for the Michael acceptors **M1**–**M73** with GSH‐derived electrophilicities *E* (orange dots) in Figure 4 b. Although the correlation coefficient decreases to *r* ^2^=0.8331 (*n=*117), the scattering range of the *E* vs. Δ*G*_MA_ correlation does not widen significantly when GSH‐based *E* values are included. Purple rhombs in Figure 4 b show additional entries for **M74**–**M82**. Their positions indicate that the *E* vs. Δ*G*_MA_ correlation for prototypical Michael acceptors also holds for roughly estimating the reactivity of the structurally more sophisticated fluorescent probes **M74**–**M82**.

**Figure 4:**
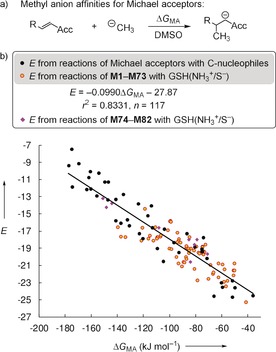
a) Definition of the methyl anion affinities (Δ*G*_MA_) of Michael acceptors. b) Correlation between experimentally determined electrophilicities *E* and methyl anion affinities Δ*G*_MA_ in DMSO [calculated at the SMD(DMSO)/B3LYP/6‐311++G(3df,2pd)//B3LYP/6‐31G(d,p) level of theory, see Supporting Information for details] supplemented by data for the fluorescent probes **M74**–**M82** (not included when calculating the correlation line).

Within a reactivity range that currently covers 40 orders of magnitude, using Equation (1) usually allows chemists to calculate second‐order rate constants within a precision of factor <100 for reactions at 20 °C, in which exactly one new C−X or C−C σ‐bond is formed.^20^ Table S6 collects 80 experimental second‐order rate constants *k*_2_^exp^ for the reactions of Michael acceptors with structurally diverse N‐, O‐, or S‐centered nucleophiles in different solvents at temperatures between 19 and 30 °C. The comparison with second‐order rate constants *k*_2_^eq1^ (20 °C) calculated by using Equation (1) and the reactivity parameters *E*, *N*, and *s*_N_ shows that *k*_2_^exp^/*k*_2_^eq1^<100 is fulfilled for 71 (of 80) Michael additions (Table S6). This analysis confirms that the GSH‐derived *E* values for Michael acceptors can also be used to predict reaction rates for Michael additions of other classes of nucleophiles.^21^ Rates of Corey–Chaykovsky cyclopropanations,^22^ stepwise Huisgen reactions,^10^ Weitz–Scheffer epoxidations,^23^ cyanoethylations,^24^ or simple 1,4‐additions of Michael acceptors with amines, alkoxide ions, and thiolates^24^ can thus be assessed by using Equation (1) and the available reactivity parameters *E*, *N*, and *s*_N_.^12^

In conclusion, the nucleophilic reactivity for the thiolate GSH(NH_3_^+^/S^−^) in water (*N*=20.97; *s*_N_=0.56) has been established. If *k*_2_=10^−3^ m ^−1^ s^−1^ is considered as a minimum rate constant for practical reactions at 20 °C,11c GSH(NH_3_^+^/S^−^) can be expected to react successfully with electrophiles of *E*>−26. This limiting *E* value may give orientation for the future development of so‐called “warheads” in covalently inhibiting drugs.^2^, ^6^ The applicability of Mayr reactivity parameters for the rational design of dynamic real‐time GSH‐selective fluorescent probes has already been shown.4a The conversion of GSH reactivities (lg *k*_GSH_) into Mayr *E* values (Tables S4 and S7), as proposed in this work, enables the prediction of relevant second‐order rate constants for competing nucleophilic sites, which is a frequent challenge in the development of thiol‐selective probe molecules.^3^, ^4^ Furthermore, the GSH‐based estimated electrophilicities of >70 Michael acceptors provide new insight into general structure–reactivity relationships (Figure S4).6g The estimated Mayr *E* parameters considerably enrich the structural diversity in the chemist's toolkit for the systematic prediction of thus far unexplored 1,4‐additions of Michael acceptors to a wide range of C‐, N‐, P‐, O‐, and S‐centered nucleophiles for which *N* and *s*_N_ are known^12^ (Figure S5).

## Conflict of interest

The authors declare no conflict of interest.

## Supporting information

As a service to our authors and readers, this journal provides supporting information supplied by the authors. Such materials are peer reviewed and may be re‐organized for online delivery, but are not copy‐edited or typeset. Technical support issues arising from supporting information (other than missing files) should be addressed to the authors.

Supplementary

Supplementary

## References

[1] R. M. LoPachin , T. Gavin , Free Radical Res. 2016, 50, 195–202. 26559119 10.3109/10715762.2015.1094184 PMC4949956

[2a] T. W. Schultz , J. W. Yarbrough , E. L. Johnson , SAR QSAR Environ. Res. 2005, 16, 313–322; 16234173 10.1080/10659360500204152

[2b] J. W. Yarbrough , T. W. Schultz , Chem. Res. Toxicol. 2007, 20, 558–562; 17319700 10.1021/tx600344a

[2c] A. Böhme , D. Thaens , A. Paschke , G. Schüürmann , Chem. Res. Toxicol. 2009, 22, 742–750; 19317512 10.1021/tx800492x

[2d] A. Böhme , A. Laqua , G. Schüürmann , Chem. Res. Toxicol. 2016, 29, 952–962. 27096880 10.1021/acs.chemrestox.5b00398

[3a] X. Jiang , J. Chen , A. Bajić , C. Zhang , X. Song , S. L. Carroll , Z.-L. Cai , M. Tang , M. Xue , N. Cheng , C. P. Schaaf , F. Li , K. R. MacKenzie , A. C. M. Ferreon , F. Xia , M. C. Wang , M. Maletić-Savatić , J. Wang , Nat. Commun. 2017, 8, 16087; 28703127 10.1038/ncomms16087 PMC5511354

[3b] C. Cossetti , G. Di Giovamberardino , R. Rota , A. Pastore , Nat. Commun. 2018, 9, 1588. 29686354 10.1038/s41467-018-04035-9 PMC5913264

[4a] K. Umezawa , M. Yoshida , M. Kamiya , T. Yamasoba , Y. Urano , Nat. Chem. 2017, 9, 279–286; 28221345 10.1038/nchem.2648

[4b] G. Yin , T. Niu , T. Yu , Y. Gan , X. Sun , P. Yin , H. Chen , Y. Zhang , H. Li , S. Yao , Angew. Chem. Int. Ed. 2019, 58, 4557–4561; 10.1002/anie.201813935 30742366

[5a] X. Chen , Y. Zhou , X. Peng , J. Yoon , Chem. Soc. Rev. 2010, 39, 2120–2135; 20502801 10.1039/b925092a

[5b] S. Lee , J. Li , X. Zhou , J. Yin , J. Yoon , Coord. Chem. Rev. 2018, 366, 29–68.

[6a] S. Amslinger , ChemMedChem 2010, 5, 351–356; 20112330 10.1002/cmdc.200900499

[6b] I. M. Serafimova , M. A. Pufall , S. Krishnan , K. Duda , M. S. Cohen , R. L. Maglathlin , J. M. McFarland , R. M. Miller , M. Frödin , J. Taunton , Nat. Chem. Biol. 2012, 8, 471–476; 22466421 10.1038/nchembio.925 PMC3657615

[6c] N. J. Pace , E. Weerapana , ACS Chem. Biol. 2013, 8, 283–296; 23163700 10.1021/cb3005269

[6d] S. Krishnan , R. M. Miller , B. Tian , R. D. Mullins , M. P. Jacobson , J. Taunton , J. Am. Chem. Soc. 2014, 136, 12624–12630; 25153195 10.1021/ja505194w PMC4160273

[6e] E. H. Krenske , R. C. Petter , K. N. Houk , J. Org. Chem. 2016, 81, 11726–11733; 27934455 10.1021/acs.joc.6b02188

[6f] R. Lonsdale , J. Burgess , N. Colclough , N. L. Davies , E. M. Lenz , A. L. Orton , R. A. Ward , J. Chem. Inf. Model. 2017, 57, 3124–3137; 29131621 10.1021/acs.jcim.7b00553

[6g] P. A. Jackson , J. C. Widen , D. A. Harki , K. M. Brummond , J. Med. Chem. 2017, 60, 839–885; 27996267 10.1021/acs.jmedchem.6b00788 PMC5308545

[6h] J. M. Strelow , SLAS Discov. 2017, 22, 3–20; 27703080 10.1177/1087057116671509

[6i] Z. Zhao , P. E. Bourne , Drug Discovery Today 2018, 23, 727–735; 29337202 10.1016/j.drudis.2018.01.035

[6j] R. Lonsdale , R. A. Ward , Chem. Soc. Rev. 2018, 47, 3816–3830; 29620097 10.1039/c7cs00220c

[6k] A. Keeley , P. Ábrányi-Balogh , G. M. Keserü , MedChemComm 2019, 10, 263–267; 30881613 10.1039/c8md00327k PMC6390469

[6l] for the kinetics of GSH binding to a series of acrylamides at 37 °C, see: V. J. Cee , L. P. Volak , Y. Chen , M. D. Bartberger , C. Tegley , T. Arvedson , J. McCarter , A. S. Tasker , C. Fotsch , J. Med. Chem. 2015, 58, 9171–9178. 26580091 10.1021/acs.jmedchem.5b01018

[7a] M. Friedman , J. F. Cavins , J. S. Wall , J. Am. Chem. Soc. 1965, 87, 3672–3682;

[7b] H. Esterbauer , H. Zollner , N. Scholz , Z. Naturforsch. C 1975, 30, 466–473. 241172 10.1515/znc-1975-7-808

[8a] G. Eisenbrand , J. Schuhmacher , P. Gölzer , Chem. Res. Toxicol. 1995, 8, 40–46; 7703365 10.1021/tx00043a005

[8b] K. Chan , R. Poon , P. J. O'Brien , J. Appl. Toxicol. 2008, 28, 1027–1039. 18626890 10.1002/jat.1369

[9] J. A. H. Schwöbel , D. Wondrousch , Y. K. Koleva , J. C. Madden , M. T. D. Cronin , G. Schüürmann , Chem. Res. Toxicol. 2010, 23, 1576–1585. 20882991 10.1021/tx100172x

[10] D. S. Allgäuer , H. Jangra , H. Asahara , Z. Li , Q. Chen , H. Zipse , A. R. Ofial , H. Mayr , J. Am. Chem. Soc. 2017, 139, 13318–13329. 28921959 10.1021/jacs.7b05106

[11a] H. Mayr , M. Patz , Angew. Chem. Int. Ed. Engl. 1994, 33, 938–957;

[11b] H. Mayr , T. Bug , M. F. Gotta , N. Hering , B. Irrgang , B. Janker , B. Kempf , R. Loos , A. R. Ofial , G. Remennikov , H. Schimmel , J. Am. Chem. Soc. 2001, 123, 9500–9512; 11572670 10.1021/ja010890y

[11c] H. Mayr , A. R. Ofial , SAR QSAR Environ. Res. 2015, 26, 619–646. 26315811 10.1080/1062936X.2015.1078409

[12] A free database of reactivity parameters *E*, *N*, and *s* _N_ can be accessed at: http://www.cup.lmu.de/oc/mayr/DBintro.html.

[13] R. J. Mayer , N. Hampel , P. Mayer , A. R. Ofial , H. Mayr , Eur. J. Org. Chem. 2019, 412–421. 10.1021/acs.joc.9b01485 31241938

[14a] J. A. H. Schwöbel , Y. K. Koleva , S. J. Enoch , F. Bajot , M. Hewitt , J. C. Madden , D. W. Roberts , T. W. Schultz , M. T. D. Cronin , Chem. Rev. 2011, 111, 2562–2596; 21401043 10.1021/cr100098n

[14b] for the detection of the thermodynamically preferred, terminal glutamyl N-bound 1:1 adducts of GSH and hexenones by MS techniques after 24 h reaction time, see: C. Slawik , C. Rickmeyer , M. Brehm , A. Böhme , G. Schüürmann , Environ. Sci. Technol. 2017, 51, 4018–4026. 28225253 10.1021/acs.est.6b04981

[15a] D. L. Rabenstein , J. Am. Chem. Soc. 1973, 95, 2797–2803;

[15b] A. Mirzahosseini , M. Somlyay , B. Noszál , Chem. Phys. Lett. 2015, 622, 50–56; 10.1021/acs.jpcb.5b05708 26172610

[15c] the pH-dependent thiolate concentrations for GSH(NH_3_ ^+^/S^−^) and GSH(NH_2_/S^−^) were calculated by using the GSH ionization scheme of Rabenstein (Ref. [15a]) and the corresponding microscopic ionization constants from Ref. [15b] (see the Supporting Information for details);

[15d] for the SH acidity of AcCys (p*K* _a_ 9.62), see: A. Meißner , P. Gockel , H. Vahrenkamp , Chem. Ber. 1994, 127, 1235–1241.

[16] F. Brotzel , H. Mayr , Org. Biomol. Chem. 2007, 5, 3814–3820. 18004461 10.1039/b713778h

[17] Second-order rate constants determined at different temperatures (from 20 to 30 °C) were used indiscriminately to construct Figure 3.

[18] We estimate that *E*(**M1**–**M73**) may be assessed with a precision of ±2 units in *E* if only rate constants from kinetic GSH assays are available.

[19a] X. Jiang , Y. Yu , J. Chen , M. Zhao , H. Chen , X. Song , A. J. Matzuk , S. L. Carroll , X. Tan , A. Sizovs , N. Cheng , M. C. Wang , J. Wang , ACS Chem. Biol. 2015, 10, 864–874; 25531746 10.1021/cb500986w PMC4371605

[19b] J. Chen , X. Jiang , S. L. Carroll , J. Huang , J. Wang , Org. Lett. 2015, 17, 5978–5981; 26606171 10.1021/acs.orglett.5b02910 PMC4727754

[19c] O. García-Beltrán , C. González , E. G. Pérez , B. K. Cassels , J. G. Santos , D. Millán , N. Mena , P. Pavez , M. E. Aliaga , J. Phys. Org. Chem. 2012, 25, 946–952.

[20] H. Mayr , Angew. Chem. Int. Ed. 2011, 50, 3612–3618;

[21] Only for methyl crotonate (**M61**), the ratio *k* _2_ ^exp^/*k* _2_ ^eq1^ slightly exceeds a factor of 100 for five of six available *k* _2_ ^exp^ values at 30 °C, which may in part be due to the 10 K difference in the reference temperatures for *k* _2_ ^exp^ and *k* _2_ ^eq1^.

[22] R. Appel , N. Hartmann , H. Mayr , J. Am. Chem. Soc. 2010, 132, 17894–17900. 21114316 10.1021/ja1084749

[23a] R. J. Mayer , T. Tokuyasu , P. Mayer , J. Gomar , S. Sabelle , B. Mennucci , H. Mayr , A. R. Ofial , Angew. Chem. Int. Ed. 2017, 56, 13279–13282; 10.1002/anie.201707086 28815833

[23b] R. J. Mayer , A. R. Ofial , Org. Lett. 2018, 20, 2816–2820; 29741385 10.1021/acs.orglett.8b00645

[23c] R. J. Mayer , A. R. Ofial , Eur. J. Org. Chem. 2018, 6010–6017.

[24] For references, see Table S6 in the Supporting Information.

