# Supplementary material for: Nucleophilicity of Glutathione: A Link to Michael Acceptor Reactivities
Source: Angew Chem Int Ed Engl. 2019 Oct 31;58(49):17704–8. doi: 10.1002/anie.201909803 (PMC6899611; doi:10.1002/anie.201909803)
Supplement: Supplementary file 2 — Supplementary [file ANIE-58-17704-s002.pdf]

Angewandte Chemie
Eine Zeitschrift der Gesellschaft Deutscher Chemiker

Supporting Information

Nucleophilicity of Glutathione: A Link to Michael Acceptor Reactivities

*Robert J. Mayer and Armin R. Ofial**

anie_201909803_sm_miscellaneous_information.pdf
anie_201909803_sm_geometries_MAA.sdf

# – Supporting Information –

CONTENT

General **S2**

Product Analysis **S3**

GSH Microscopic Protonation Constants **S9**

Relative Reactivity of GSH(NH_3^+/S^-) and GSH(NH_2/S^-) **S11**

Kinetics **S15**

Kinetics of GSH with cationic and neutral reference electrophiles S16

Kinetics of AcCys with cationic and neutral reference electrophiles S21

Electrophilicity of Michael Acceptors towards GSH in Kinetic Assays **S25**

Quantum-Chemically Calculated Methyl Anion Affinities (ΔG_{MA}) of Michael Acceptors **S32**

Applications **S42**

#1: Prediction of rate constants for the reactions of Michael acceptors with nucleophiles S42

#2: Estimating the electrophilicity of fluorescent thiol probes S50

#3: Quantitative structure/reactivity relationships for α,β-unsaturated carbonyl compounds S56

#4: Rational prediction of Michael additions S57

References **S59**

General

Reagents

Glutathione (GSH) and acetyl cysteine (AcCys) were purchased from Sigma-Aldrich. Stock solutions of potassium hydroxide (KOH) were purchased from Bernd Kraft Laborchemikalien.

Analytics

Melting points were acquired using a Büchi Melting Point B-560 device and are not corrected.

Nuclear magnetic resonance spectra were recorded on 400 MHz NMR spectrometers. The following abbreviations and their combinations are used in the analysis of NMR spectra: s = singlet, d = doublet, t = triplet, q = quartet, m = multiplet, br s = broad singlet. NMR signals were assigned based on information from additional 2D NMR experiments (COSY, gHSQC, gHMBC, NOESY). Internal reference was set to the residual solvent signals (δ_H = 4.79 for D_2O). The ^{13}C NMR spectra were recorded under broad-band proton-decoupling.

Infrared (IR) spectra were recorded on a Perkin Elmer Spectrum BX-59343 instrument with a Smiths Detection DuraSamplIR II Diamond ATR sensor for detection in the range 4500–600 cm^{-1}.

High resolution (HRMS) mass spectra were recorded on a Thermo Finnigan LTQ FT Ultra Fourier Transform ion cyclotron resonance mass spectrometer. For ionization of the samples, electrospray ionization (ESI) was applied.

Kinetic measurements

Kinetics of reactions in alkaline, aqueous solution (degassed and prepared under an atmosphere of N_2) were followed by employing stopped-flow UV/Vis photometry on Applied Photophysics SX20 systems. The temperature (20.0 ± 0.2 °C) was controlled by using circulating bath cryostats. Acetonitrile was used as a co-solvent (0.5 or 11 vol-%) to solubilize the electrophiles.

Product Analysis

GS-E1·HBF_4 (RM551)

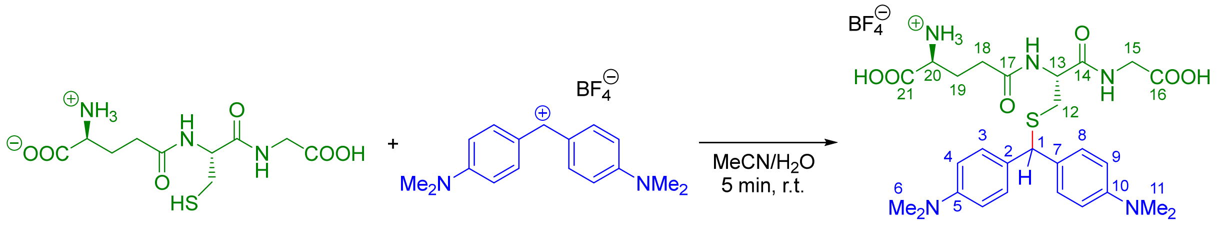

Glutathione (GSH, 35.2 mg, 0.115 mmol) was dissolved in water (5 mL). Under stirring, a solution of **E1**-BF_4 (39.0 mg, 0.115 mmol) in acetonitrile (2 mL) was added dropwise. The resulting pale greenish suspension was stirred for another 2 min. Then all solvents were removed in the vacuum. The residual highly viscous green oil slowly solidified to yield a pale green solid: **GS-E1**·HBF_4 (74.2 mg, yield: quant.).

^{1}H NMR (400 MHz, D_2O): δ = 7.68–7.64 (m, 4 H, 3-H and 8-H), 7.55–7.51 (m, 4 H, 4-H and 9-H), 5.48 (s, 1 H, 1-H), 4.42 (dd, J = 8.7 Hz, J = 5.1 Hz, 1 H, 13-H), 3.84–3.81 (m, 2 H, 15-H), 3.79 (t, J = 6.4 Hz, 1 H, 20-H), 3.234 (s, 6 H, 6-H or 11-H), 3.230 (s, 6 H, 6-H or 11-H), 3.04 (dd, ^2J = 14.3 Hz, ^3J = 5.1, 1 H, 12-H^a), 2.86 (dd, ^2J = 14.2 Hz, ^3J = 8.8 Hz, 1 H, 12-H^b), 2.55–2.51 (m, 2 H, 18-H), 2.20–2.14 (m, 2 H, 19-H).

^{13}C{^{1}H} NMR (101 MHz, D_2O): δ = 174.9 (C_q, C-16), 174.5 (C_q, C-17), 173.7 (C_q, C-21), 171.7 (C_q, C-14), 142.2 (C_q, C-5 and C-10), 141.6 (C-2), 141.3 (C-7), 130.0 (CH, C-3 and C-8), 120.3 (CH, C-4 and C-9), 53.9 (CH, C-20), 52.7 (CH, C-13), 52.0 (CH, C-1), 45.70 (CH_3, C-6 or C-11), 45.66 (CH_3, C-6 or C-11), 42.5 (CH_2, C-15), 33.5 (CH_2, C-12), 31.4 (CH_2, C-18), 26.2 (CH_2, C-19).

HRMS (ESI$^+$): Calcd m/z for $[C_{27}H_{38}N_5O_6S]^+$ [M + H$^+$]: 560.2537; Found: 560.2548.

IR (ATR, neat): 2934, 2509, 1726, 1609, 1518, 1448, 1410, 1351, 1228, 1054, 806, 764 cm^{-1}.

mp 140–145 °C (the sample turned bright blue at 80 °C and then dark blue at 120 °C; the exact melting point could therefore not be determined).

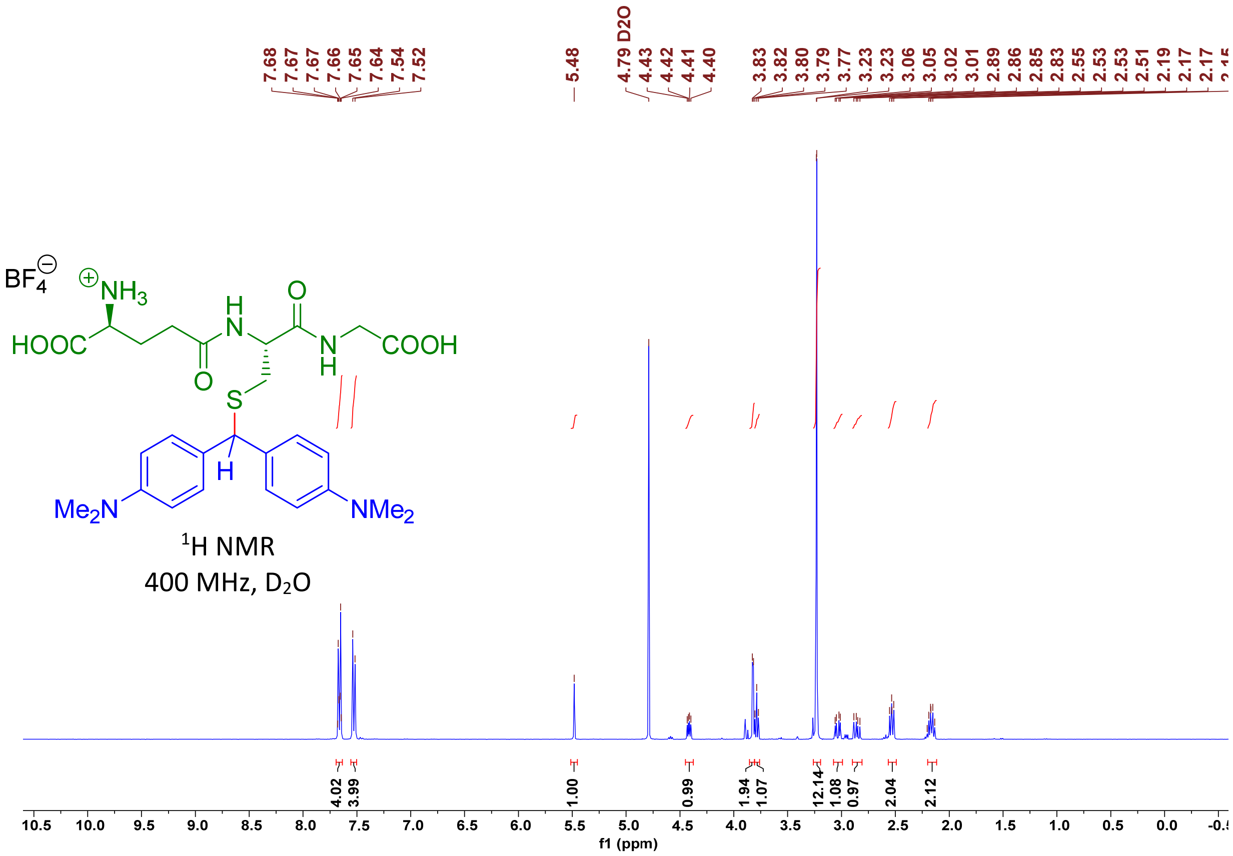
7.68
7.67
7.67
7.66
7.65
7.64
7.54
7.52
5.48
4.79 D2O
4.43
4.42
4.41
4.40
3.83
3.82
3.80
3.79
3.77
3.23
3.23
3.06
3.05
3.02
3.01
2.89
2.86
2.85
2.83
2.55
2.53
2.53
2.51
2.19
2.17
2.17
[illegible]
BF4
NH3
HOOC
H
N
O
N
H
COOH
O
S
H
Me2N
NMe2
1H NMR
400 MHz, D2O
4.02
3.99
1.00
0.99
1.94
1.07
12.14
1.08
0.97
2.04
2.12
10.5 10.0 9.5 9.0 8.5 8.0 7.5 7.0 6.5 6.0 5.5 5.0 4.5 4.0 3.5 3.0 2.5 2.0 1.5 1.0 0.5 0.0
f1 (ppm)

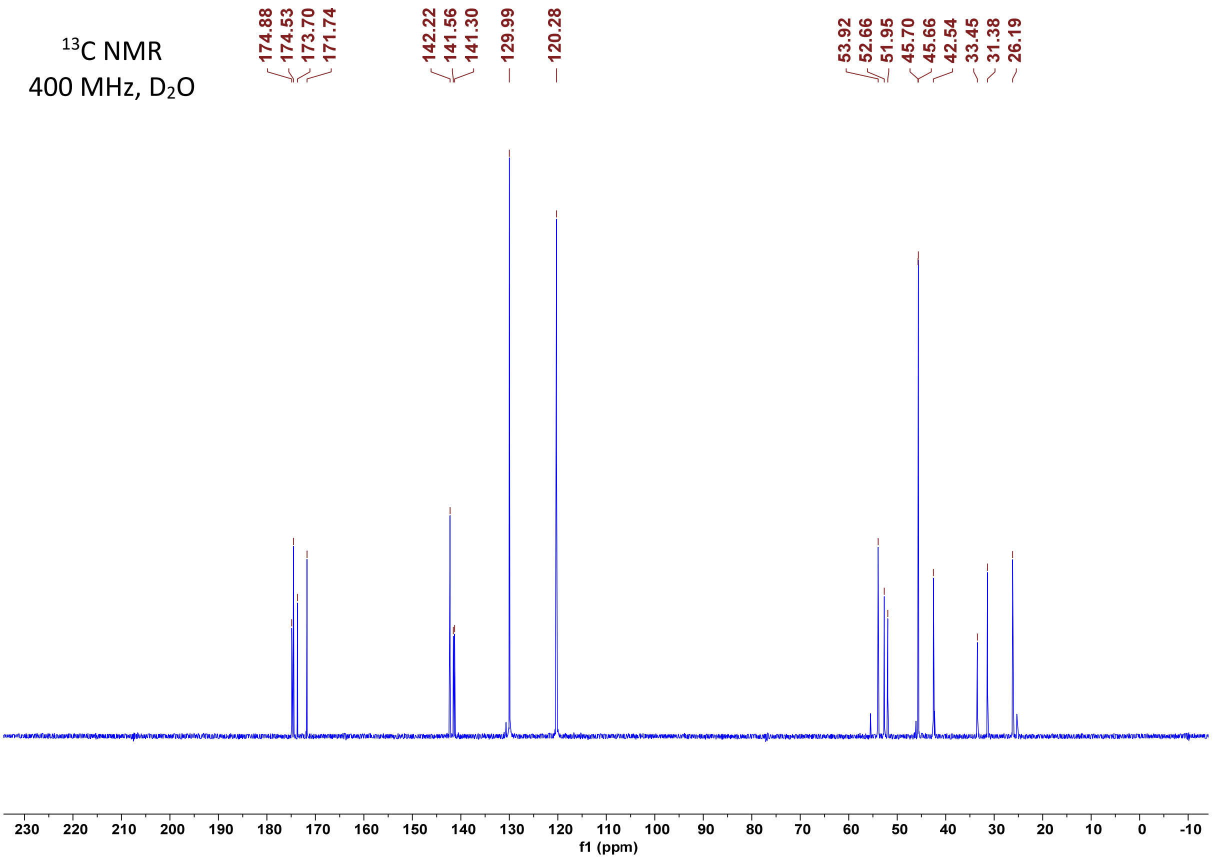
13C NMR
400 MHz, D2O
174.88
174.53
173.70
171.74
142.22
141.56
141.30
129.99
120.28
53.92
52.66
51.95
45.70
45.66
42.54
33.45
31.38
26.19
230 220 210 200 190 180 170 160 150 140 130 120 110 100 90 80 70 60 50 40 30 20 10 0 -10
f1 (ppm)

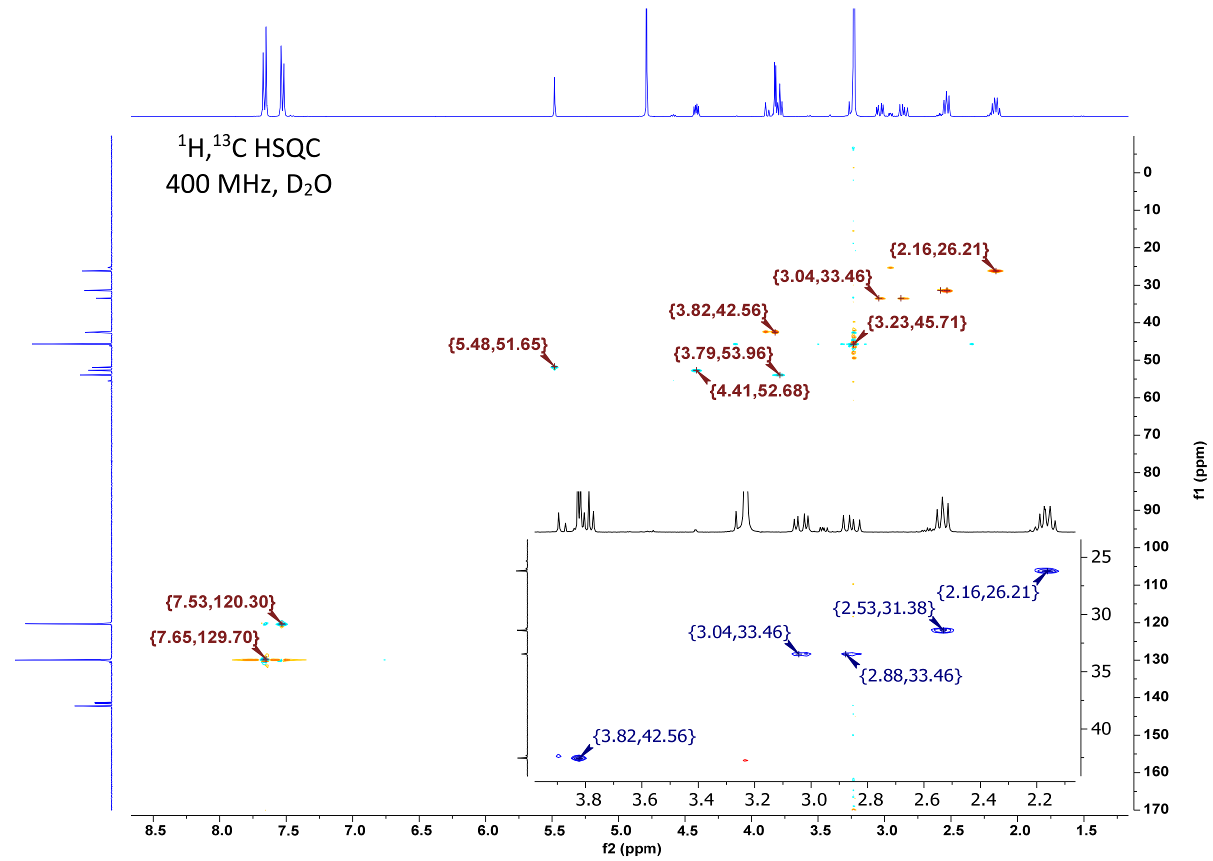

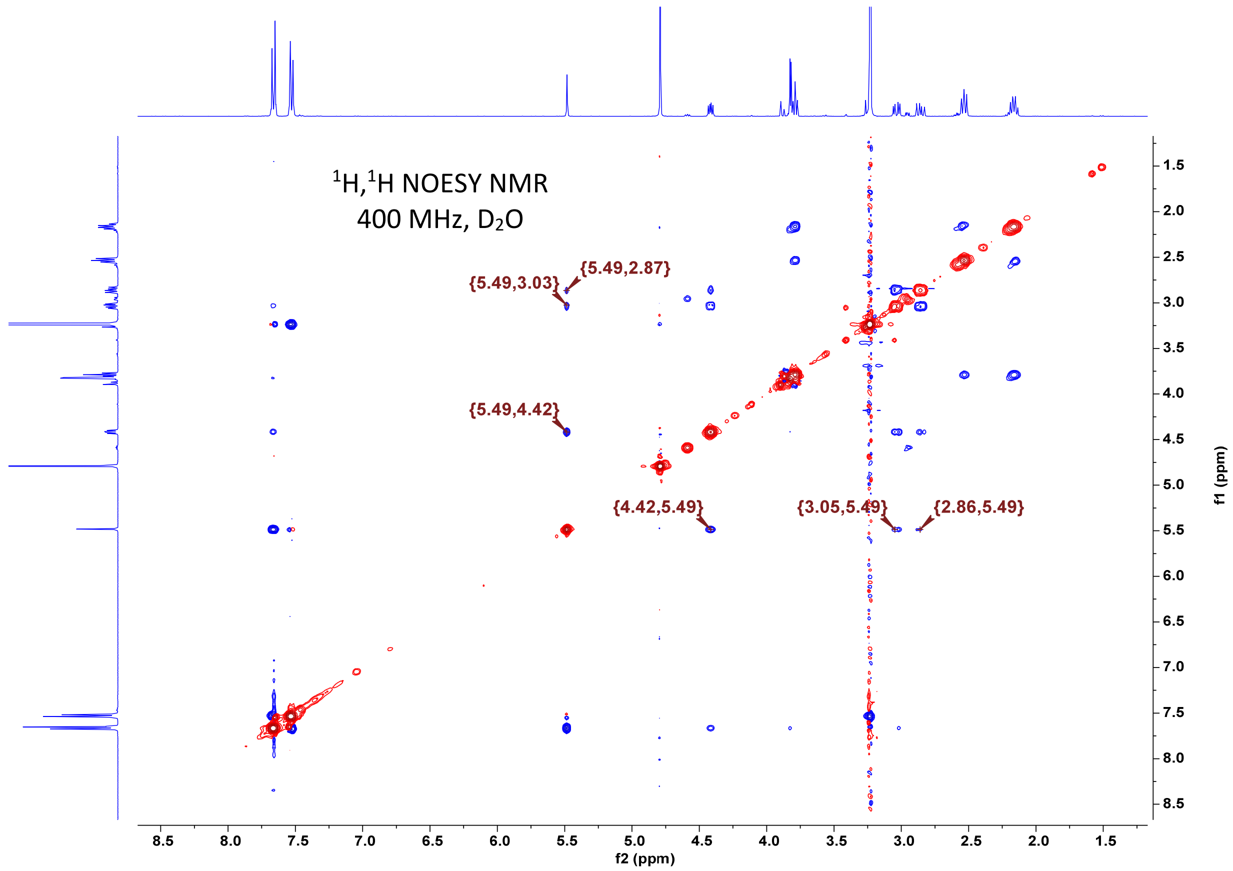

The marked resonances in the NOESY spectrum indicate the vicinity of 1-H with 12-H and 13-H.

AcCys-E1·HBF_4 (RNH45)

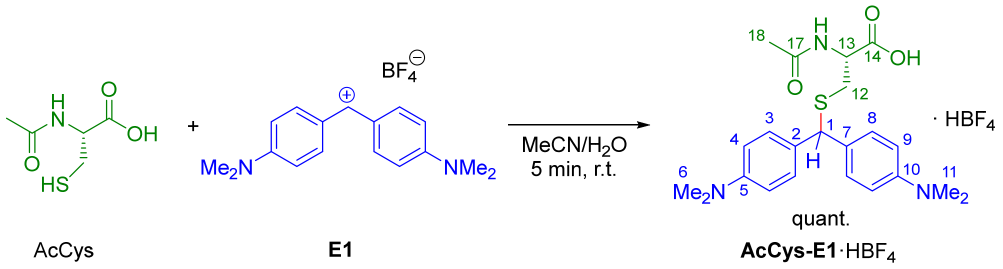

To a stirred solution of *N*-acetylcysteine (18.7 mg, 0.115 mmol) in water (5 mL) was added dropwise a solution of **E1**-BF_4 (39.0 mg, 0.115 mmol) in acetonitrile (2 mL). The resulting pale blue solution was stirred for another 2 min. Then all solvents were removed in the vacuum. The residual highly viscous green oil slowly solidified to yield a pale blue solid: **AcCys-E1**·HBF_4 (57.5 mg, 0.0114 mmol, yield: 99%).

^{1}H NMR (400 MHz, D_2O) δ = 7.71–7.66 (m, 4 H, 3-H and 8-H), 7.58–7.54 (m, 4 H, 4-H and 9-H), 5.52 (s, 1 H, 1-H), 4.30 (dd, ^3J = 8.1 Hz, ^3J = 4.5 Hz, 1 H, 13-H), 3.26 (s, 12 H, 6-H and 11-H), 2.99 (dd, ^2J = 14.0 Hz, ^3J = 4.5 Hz, 1 H, 12-H^a), 2.86 (dd, ^2J = 14.0 Hz, ^3J = 8.1 Hz, 1 H, 12-H^b), 2.03 (s, 3 H, 18-H).

^{13}C{^{1}H} NMR (101 MHz, D_2O) δ = 175.8 (C_q, C-14), 173.4 (C_q, C-17), 142.1 (C_q), 141.9 (C_q), 141.83 (C_q), 141.81 (C_q), 130.09 (CH, C-3 or C-8), 130.07 (CH, C-3 or C-8), 120.44 (CH, C-4 or C-9), 120.39 (CH, C-4 or C-9), 53.7 (CH, C-13), 51.8 (CH, C-1), 45.88 (CH_3, C-6 or C-11), 45.86 (CH_3, C-6 or C-11), 34.0 (CH_2, C-12), 21.8 (CH_3, C-18).

HRMS (ESI$^-$): Calcd m/z for $[C_{22}H_{28}N_3O_3S]^-$: 414.1857; Found: 414.1864.

IR (ATR, neat): 2470, 1727, 1608, 1517, 1374, 1185, 1052, 900, 805, 763 cm^{-1}.

mp 55–65 °C (the sample turned green-black when heated to > 50 °C).

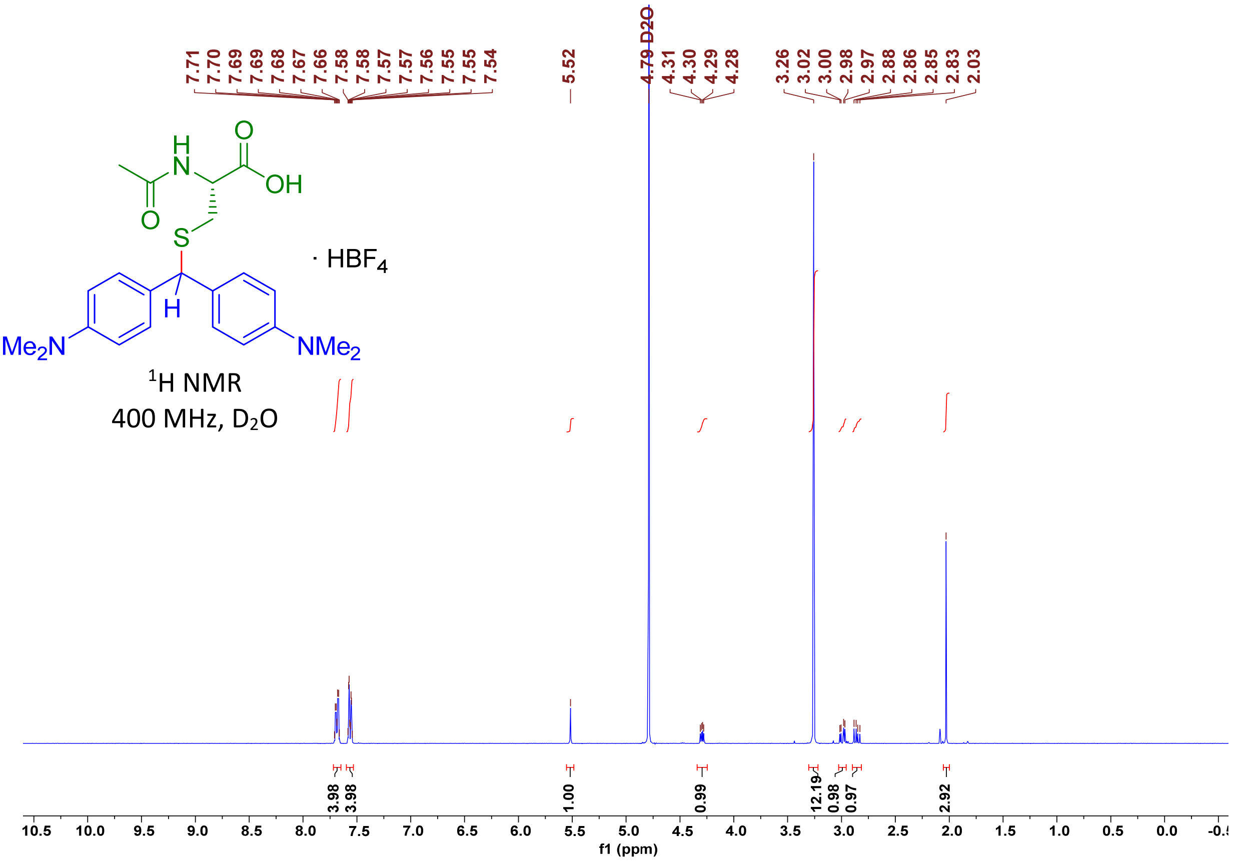
7.71 7.70 7.69 7.69 7.68 7.67 7.66 7.58 7.58 7.57 7.57 7.56 7.55 7.55 7.54
5.52
4.79 D2O
4.31 4.30 4.29 4.28
3.26 3.02 3.00 2.98 2.97 2.88 2.86 2.85 2.83 2.03
· HBF4
Me2N
NMe2
H
N
O
OH
S
1H NMR
400 MHz, D2O
3.98 3.98 1.00 0.99 12.19 0.98 0.97 2.92
f1 (ppm)

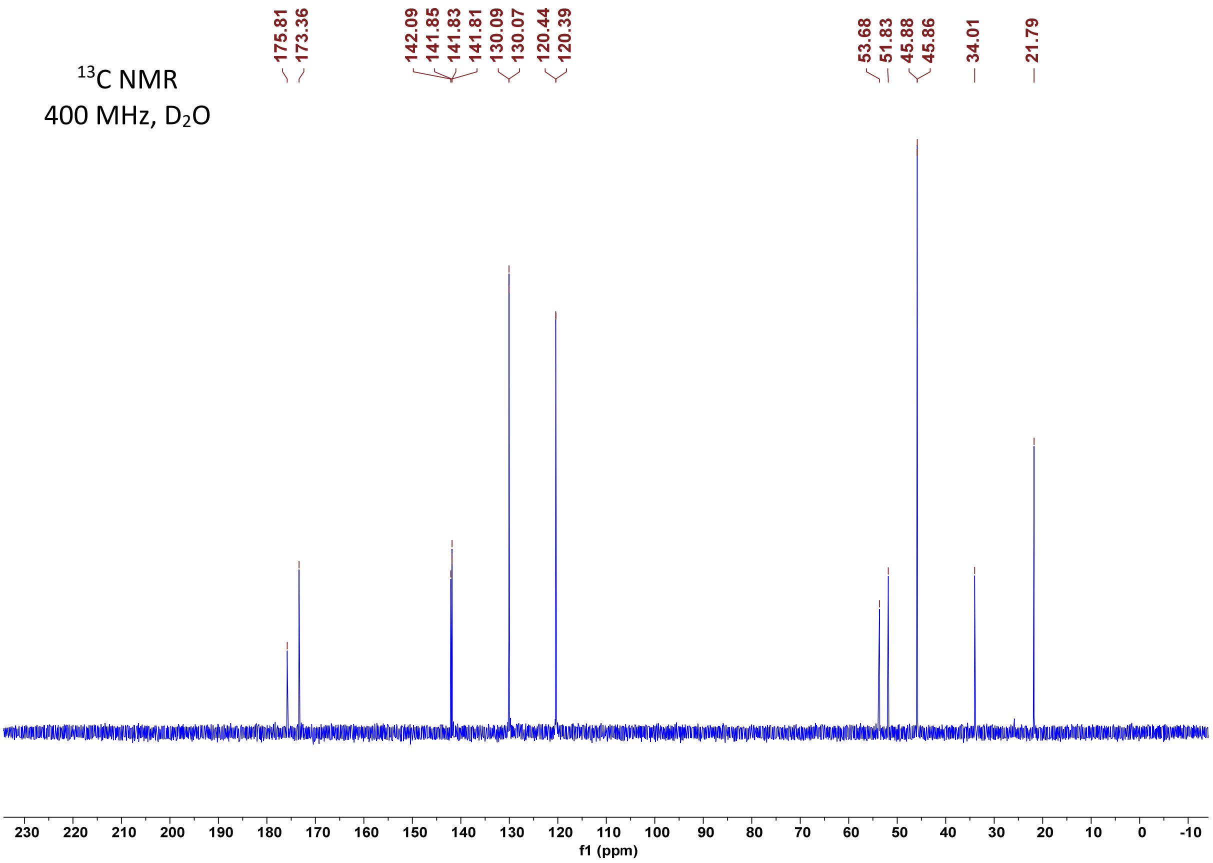
175.81 173.36
142.09 141.85 141.83 141.81 130.09 130.07 120.44 120.39
53.68 51.83 45.88 45.86
34.01
21.79
13C NMR
400 MHz, D2O
f1 (ppm)

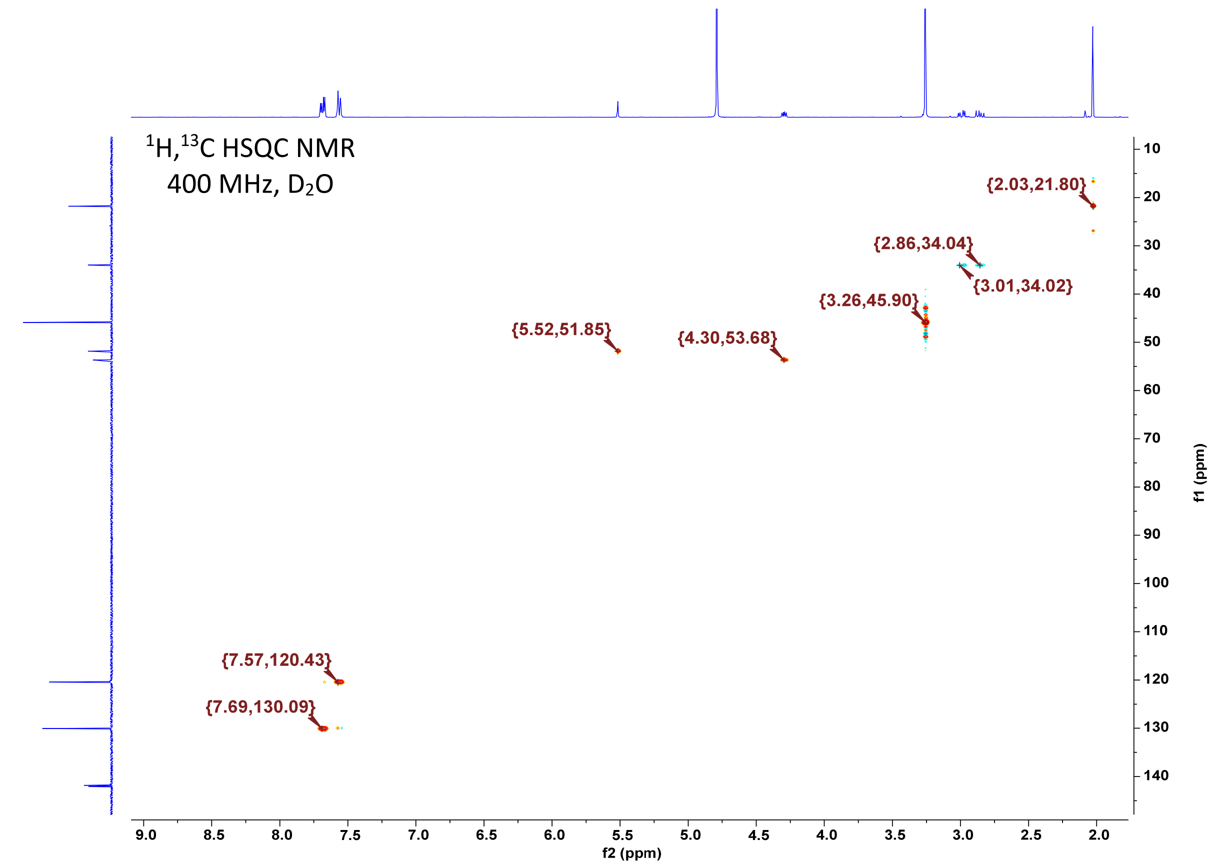

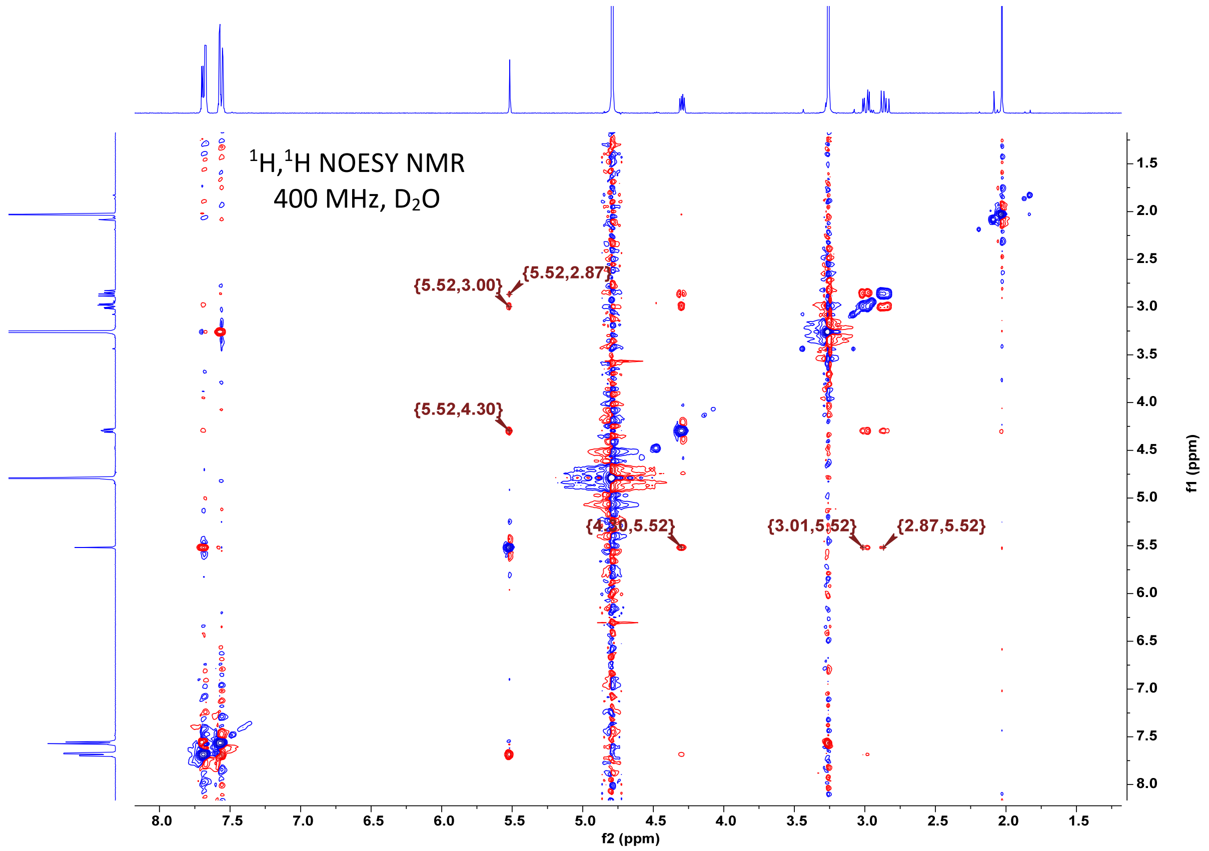

The marked resonances in the NOESY spectrum indicate the vicinity of 1-H to 12-H and 13-H.

GSH Microscopic Protonation Constants

If multiple acidic sites in a molecule dissociate simultaneously at a certain pH value, macroscopic protonation constants are hybrid and do not accurately describe the degree of ionization at the molecular level. Instead, microscopic ionizations constants are required for polyvalent acids to calculate the precise distribution of protonated and deprotonated species at a certain pH.[S1]

Details of the acid-base chemistry of glutathione (GSH) have previously been analyzed by Friedman and co-workers,[S2] Rabenstein,[S3] and more recently by Noszál and co-workers.[S4]

We used the GSH ionization scheme by Rabenstein, which involves the seven species **A** to **H** that are interconnected by eight microscopic dissociation constants (Scheme S1).[S3] In the pH range from 7 to 12, the species **D** to **H** and the dissociation constants K_{DF}, K_{DG}, K_{FH}, and K_{GH} are relevant.

Scheme S1: Protonation scheme for GSH and the related microscopic protonation constants according to Rabenstein.[S3]

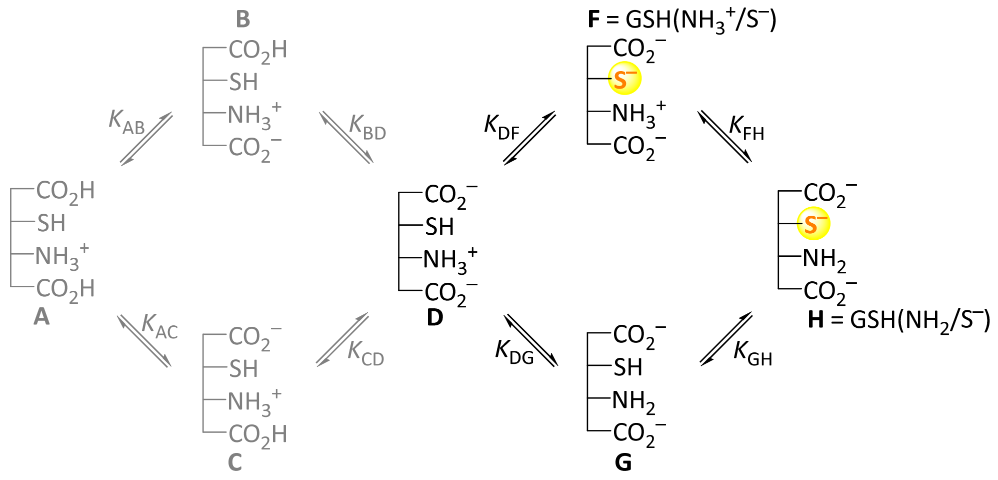

$$K_{AB} = \frac{[H^+][\mathbf{B}]}{[\mathbf{A}]} \qquad K_{BD} = \frac{[H^+][\mathbf{D}]}{[\mathbf{B}]} \qquad K_{DF} = \frac{[H^+][\mathbf{F}]}{[\mathbf{D}]} \qquad K_{FH} = \frac{[H^+][\mathbf{H}]}{[\mathbf{F}]}$$

$$K_{AC} = \frac{[H^+][\mathbf{C}]}{[\mathbf{A}]} \qquad K_{CD} = \frac{[H^+][\mathbf{D}]}{[\mathbf{C}]} \qquad K_{DG} = \frac{[H^+][\mathbf{G}]}{[\mathbf{D}]} \qquad K_{GH} = \frac{[H^+][\mathbf{H}]}{[\mathbf{G}]}$$

At a certain pH, the fraction F_i of each species i present in the equilibrium can be calculated from the microscopic dissociation constants as exemplified for species **F** [= GSH(NH_3^+/S^-)] in equation S1 and for species **H** [= GSH(NH_2/S^-)] in equation S2.

$$\text{for GSH}(NH_3^+/S^-):\quad F_{\mathbf{F}} = \frac{[\mathbf{F}]}{[\mathbf{D}] + [\mathbf{F}] + [\mathbf{G}] + [\mathbf{H}]} = \frac{K_{DF}/K_{DG}}{[H^+]/K_{DG} + K_{DF}/K_{DG} + 1 + K_{GH}/[H^+]} \qquad (S1)$$

$$\text{for GSH}(NH_2/S^-):\quad F_{\mathbf{H}} = \frac{[\mathbf{H}]}{[\mathbf{D}] + [\mathbf{F}] + [\mathbf{G}] + [\mathbf{H}]} = \frac{K_{GH}/[H^+]}{[H^+]/K_{DG} + K_{DF}/K_{DG} + 1 + K_{GH}/[H^+]} \qquad (S2)$$

Analogously, the total fraction of reactive nucleophilic species, for example, for GSH species with a free thiolate group (F_{S-}) or GSH species with a free amino group (F_{NH2}) can be calculated by equations S3 and S4, respectively.

$$F_{S^-} = F_{\mathbf{F}} + F_{\mathbf{H}} = \frac{[\mathbf{F}] + [\mathbf{H}]}{[\mathbf{D}] + [\mathbf{F}] + [\mathbf{G}] + [\mathbf{H}]} = \frac{K_{DF}/K_{DG} + K_{GH}/[H^+]}{[H^+]/K_{DG} + K_{DF}/K_{DG} + 1 + K_{GH}/[H^+]} \qquad (S3)$$

$$F_{NH2} = F_{\mathbf{G}} + F_{\mathbf{H}} = \frac{[\mathbf{G}] + [\mathbf{H}]}{[\mathbf{D}] + [\mathbf{F}] + [\mathbf{G}] + [\mathbf{H}]} = \frac{1 + K_{GH}/[H^+]}{[H^+]/K_{DG} + K_{DF}/K_{DG} + 1 + K_{GH}/[H^+]} \qquad (S4)$$

Microscopic protonation constants for GSH have been determined by ^1H NMR spectroscopic titration experiments.[S3,S4] In this work, we used the microscopic protonation constants by Noszál and co-workers (Table S1) who followed the changes of ^1H NMR chemical shifts in the GSH protonation equilibria by using a 600 MHz NMR spectrometer.[S4]

Table S1: Microscopic protonation constants for the equilibria between GSH species **D**, **F**, **G**, and **H** (in H_2O/D_2O 95:5 (v/v), at 25 °C and 0.15 mol L^{-1} ionic strength, from ref [S4])

pK_{DF}	8.94 ± 0.02
pK_{DG}	9.17 ± 0.02
pK_{FH}	9.49 ± 0.01
pK_{GH}	9.26 ± 0.01

By applying the microscopic protonation constants from Table S1 in equations S1–S3, one can calculate the pH-dependent distribution of reactive GSH species in aqueous solution. As depicted in Figure S1, at pH < 8 nucleophilic GSH reactivity is mostly caused by species **F** [= GSH(NH_3^+/S^-)]. Thiolate **F** is also the only relevant species for nucleophilic reactions of GSH under physiological conditions (pH 7.4). At pH > 11, the GSH species **H** [= GSH(NH_2/S^-)] predominates.

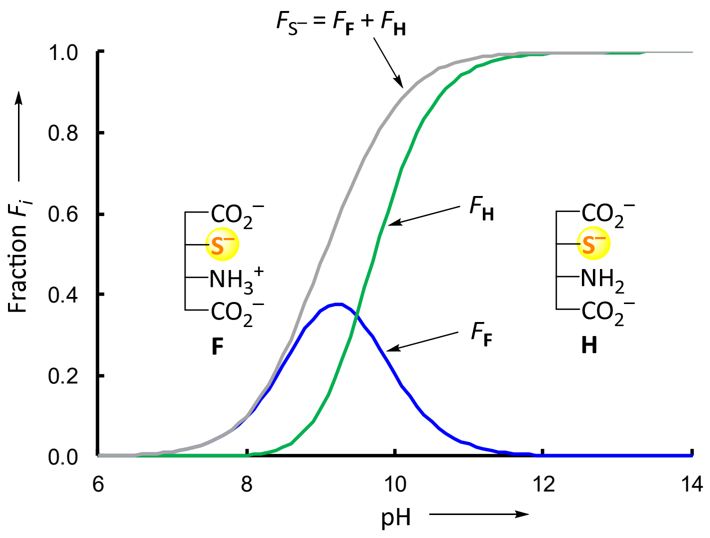

Figure S1: Distribution of GSH species **F** and **H** with a reactive thiolate group as a function of pH.

Relative Reactivity of GSH(NH_3^+/S^-) and GSH(NH_2/S^-)

Friedman showed that protonation at the remote γ-glutamyl NH_2 group (that is, **F** → **H**) reduced the GSH thiolate reactivity toward acrylonitrile by a factor of 2.[S2]

To assess whether this reactivity difference between both reactive GSH thiolate species **F** [that is, GSH(NH_3^+/S^-)] and **H** [that is, GSH(NH_2/S^-)] also holds for our series of experiments, we studied the pH-dependent reactivity of GSH with the benzhydrylium ion **E4**.

Stock solutions of GSH, Na_3PO_4, $Na_2H_2PO_4$ and NaH_2PO_4 were mixed in variable ratios to achieve a certain pH in the reaction chamber of the stopped-flow instrument. The reactions between the electrophile **E4** and the nucleophilic GSH species were then monitored by following the mono-exponential decay of the UV-Vis absorbance of the colored **E4** by using the stopped-flow technique. Experimental conditions and observed first-order rate constants k_{obs} are gathered in Table S2.

Concentrations of the reactive GSH species **F** and **H** at different pH were calculated from the relationships [**F**] = $F_{\mathbf{F}}$[GSG]$_0$ and [**H**] = $F_{\mathbf{H}}$[GSH]$_0$. Partial second-order rate constants $k_{\mathbf{F}}^{calcd}$ and $k_{\mathbf{H}}^{calcd}$ for the nucleophilic GSH species **F** and **H** were then determined by equations S5 and S6, respectively.

$$k_{\mathbf{F}}^{calcd} = 0.5 k_2[\text{GSH}]_0 F_{\mathbf{F}} \qquad \text{(S5)}$$

$$k_{\mathbf{H}}^{calcd} = k_2[\text{GSH}]_0 F_{\mathbf{H}} \qquad \text{(S6)}$$

The second-order rate constant k_2(20 °C) for the reaction of **E4** with **H** [that is, GSH(NH_2/S^-)] at pH 12 was determined experimentally in this work (k_2 = 6.60 × 10^5 M^{-1} s^{-1}, see below). The factor 0.5 in

equation S5 considers the relative reactivity of **F** and **H** towards electrophiles reported by Friedman.[S2] In this way, the overall rate constant k_{S-}^{calcd} for the reaction of of GSH with **E4** at a certain pH was obtained as the sum of both partial rate constants (equation S7).

$$k_{S-}^{calcd} = k_{\mathbf{F}}^{calcd} + k_{\mathbf{H}}^{calcd} \qquad \text{(S7)}$$

Table S2. Dependency of the first-order rate constants for reactions of GSH ($[GSH]_0 = 1.00 \times 10^{-4}$ M) and **E4** ($[\mathbf{E4}]_0 = 5.45 \times 10^{-6}$ M) on the pH of the aqueous solution (20 °C).

pH[a]	k_{obs} (s^{-1})	lg k_{obs}	$F_{\mathbf{F}}$	$k_{\mathbf{F}}^{calcd}$ (s^{-1})	$F_{\mathbf{H}}$	$k_{\mathbf{H}}^{calcd}$ (s^{-1})	lg k_{S-}^{calcd}
11.93	5.66×10^{1}	**1.75**	3.61×10^{-3}	1.19×10^{-1}	9.94×10^{-1}	65.6×10^{1}	**1.82**
11.67	4.80×10^{1}	**1.68**	6.54×10^{-3}	2.16×10^{-1}	9.90×10^{-1}	65.3×10^{1}	**1.82**
11.41	5.43×10^{1}	**1.73**	1.18×10^{-2}	3.89×10^{-1}	9.81×10^{-1}	6.48×10^{1}	**1.81**
10.85	5.39×10^{1}	**1.74**	4.08×10^{-2}	1.35	9.35×10^{-1}	6.17×10^{1}	**1.80**
10.40	5.37×10^{1}	**1.73**	1.03×10^{-1}	3.38	8.34×10^{-1}	5.50×10^{1}	**1.77**
9.81	4.37×10^{1}	**1.64**	2.62×10^{-1}	8.65	5.48×10^{-1}	3.62×10^{1}	**1.65**
9.56	3.93×10^{1}	**1.59**	3.33×10^{-1}	1.10×10^{1}	3.91×10^{-1}	2.58×10^{1}	**1.57**
9.23	2.67×10^{1}	**1.43**	3.77×10^{-1}	1.24×10^{1}	2.07×10^{-1}	1.37×10^{1}	**1.42**
8.64	1.79×10^{1}	**1.25**	2.68×10^{-1}	8.86	3.79×10^{-2}	2.50	**1.06**
8.56	8.92	**0.95**	2.44×10^{-1}	8.04	2.86×10^{-2}	1.89	**1.00**
8.45	1.25×10^{1}	**1.10**	2.10×10^{-1}	6.92	1.91×10^{-2}	1.26	**0.91**
8.01	2.45	**0.39**	9.87×10^{-2}	3.26	3.27×10^{-3}	2.16×10^{-1}	**0.54**
7.17	1.03	**0.01**	1.65×10^{-2}	5.46×10^{-1}	7.91×10^{-5}	5.22×10^{-3}	**–0.26**
6.89	2.92×10^{-1}	**–0.53**	8.79×10^{-3}	2.90×10^{-1}	2.21×10^{-5}	1.46×10^{-3}	**–0.54**
6.12	6.87×10^{-2}	**–1.16**	1.51×10^{-3}	4.98×10^{-2}	6.44×10^{-7}	4.25×10^{-5}	**–1.30**
4.90	4.92×10^{-3}	**–2.31**	9.12×10^{-5}	3.01×10^{-3}	2.34×10^{-9}	1.55×10^{-7}	**–2.52**

[a] An aliquot of each GSH/phosphate buffer solution was mixed with water in a 1:1 ratio (as in the reaction chamber of the stopped-flow spectrometer) and the pH value was determined.

Figures S2 compares the observed pH-dependent reactivity of GSH towards electrophile **E4** (lg k_{obs}) with the predicted behavior for the thiolate species of GSH (lg k_{S-}^{calcd}). The fact that the experimental data points (black dots, from column 3 in Table S2) are located in close vicinity to the predicted rate constants lg k_{S-}^{calcd} (dashed grey line, from last column in Table S2) demonstrates that GSH reactivity in aqueous solutions is clearly dominated by the reactivity of the two thiolate species **F** and **H** in the pH range from 4 to 13.

Analogous calculations showed that the free amino groups in the GSH species **G** and **H** (dashed red line for lg k_{NH2}^{calcd} in Figure S2) can be expected to contribute only to a negligible extent (<0.1%) to the kinetics of the GSH reactions with the electrophile **E4**. Also background reactions of **E4** with hydroxide ions or water (lg k_{OH}^{calcd}) do not play a significant role at pH > 4.

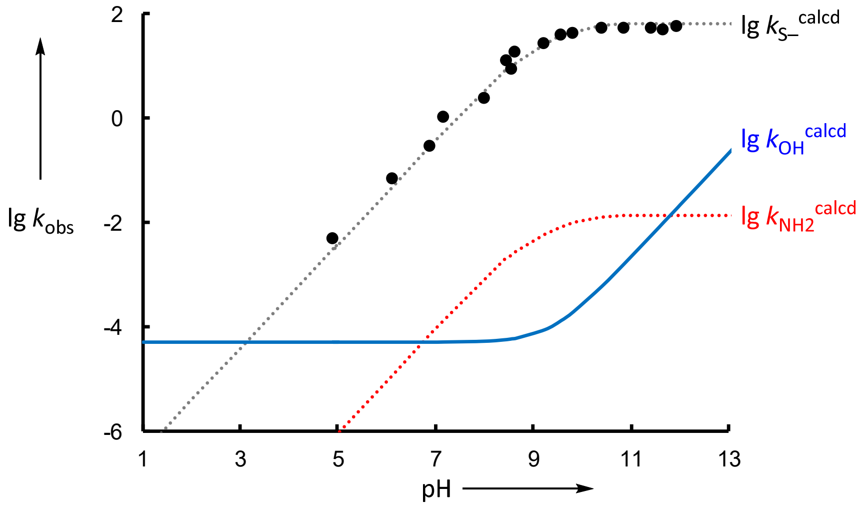

Figure S2: Observed first-order rate constants lg k_{obs} (20 °C) for the reaction of GSH with **E4** (dashed grey line: lg k_{S-}^{calcd} from Table S2; dashed red line: lg k_{NH2}^{calcd} calculated from $k_{NH2} = k_2 F_{NH2}$ by using k_2 = 140 M^{-1} s^{-1} for the reaction of glutamate with **E4**, from ref [S5b] and F_{NH2} as defined in equation S4; blue line: background reaction of **E4** with hydroxide ions and/or water).

Furthermore, the excellent agreement between lg k_{obs} and lg k_{S-}^{calcd} in Figure S2 corroborates that the relative reactivity of the GSH thiolate species **F** and **H** towards electrophiles is generally described by $k_\mathbf{F} = 0.5k_\mathbf{H}$, as initially found by Friedman and co-workers for the reaction of GSH with acrylonitrile.[S2] As a consequence, we have used the relation $k_\mathbf{F} = 0.5k_\mathbf{H}$ for converting our rate constants of GSH/electrophile reactions at pH 12 (where the reactive GSH species **H** prevails) to the more commonly used rate constants of GSH/electrophile reactions at physiological pH 7.4 (where the reactive GSH species **F** predominates).

In Table 2 (main text), we applied equation S8 to convert reported second-order rate constants k_{GSH} into second-order rate constants $k_2(GS^-)$ for the relevant thiolate reactivity of GSH.

$$k_2(GS^-) = k_{GSH}/(F_\mathbf{F} + 2F_\mathbf{H}) = k_{GSH}/F \qquad (S8)$$

The Friedman factor ($k_\mathbf{H} = 2k_\mathbf{F}$) was applied to reflect the different reactivity of the thiolate species **F** and **H** in the overall fraction F of nucleophilic thiolate ions, that is, $F = F_\mathbf{F} + 2F_\mathbf{H}$. The fractions of GSH

thiolates present at pH 7.2, 7.4, 8.0, and 8.1, previously used to determine k_{GSH} (see Table S4 below) are listed in Table S3.

Table S3. Partial fractions $F_\mathbf{F}$ and $F_\mathbf{H}$ and overall fraction F (= $F_\mathbf{F}$ + 2$F_\mathbf{H}$) of reactive GSH thiolate species in aqueous solution at different pH.

pH	$F_\mathbf{F}$	$F_\mathbf{H}$	F
7.2	0.0177	9.07×10^{-5}	0.0179
7.4	0.0276	2.24×10^{-4}	0.0280
8.0	0.0968	3.13×10^{-3}	0.103
8.1	0.117	4.77×10^{-3}	0.127

Kinetics

The aqueous solution of GSH were mixed with an equal volume of an aqueous KOH (0.04 M). Then, the obtained solution (with $[OH^-]$ = 0.02 M) was used to fill one of the reservoir syringes of the stopped-flow spectrometer. After mixing with the electrophile solution, a hydroxide concentration of $[OH^-]_0$ = 0.01 M (pH 12) was achieved in the observation cell of the stopped-flow spectrometer.

We assumed quantitative deprotonation of the Gly-COOH, the glutamyl-NH_3^+, and the cysteine-SH groups in GSH at pH 12, which consumes 3 equivs of hydroxide ions. Under the conditions of the kinetic experiments (pH 12) >99.5% of GSH was deprotonated to the thiolate **H** [that is, GSH(NH_2/S^-)]. Likewise, we assumed quantitative deprotonation of the COOH group in AcCys at pH 12, which consumes 1 equiv of hydroxide ions. The remaining OH^- concentration was then used to calculate the concentration of the thiolate $AcCys^-$ in the reaction mixture from pK_a(AcCys) 9.62[S6] according to the method described in ref [S5]. Under the conditions of the kinetic experiments (that is, at pH 12) >99% of the supplied AcCys was deprotonated to the thiolate $AcCys^-$.

By using a high excess of the nucleophilic thiolates RS^- (GS^- or $AcCys^-$) over the electrophiles **E**, the concentrations of RS^- remained almost constant during the kinetic runs, resulting in mono-exponential decays of the electrophiles' absorptions. First-order rate constants k_{obs} (s^{-1}) were obtained by least-squares fitting the single-exponential function $A_t = A_0 \exp(-k_{obs}t) + C$ to the time-dependent absorbances (averaged from typically four to eight kinetic runs at each nucleophile concentration).

The contribution of competing nucleophiles (H_2O and HO^-) to the consumption of the electrophiles during their reactions with RS^- was found to be negligible (<1% in total, cf Figure S2), which allowed us to directly plot the observed first-order rate constants k_{obs} vs $[RS^-]$ to derive the second-order rate constant k_2 (M^{-1} s^{-1}) from the slopes of the resulting linear correlations.

Kinetics of GSH with cationic and neutral reference electrophiles

E2 + GSH in alkaline 99.5/0.5 (v/v) H_2O/CH_3CN (stopped-flow, detection at 614 nm)

[**E2**]$_0$, mol L^{-1}	[GSH]$_0$, mol L^{-1}	[OH^-]$_0$, mol L^{-1}	[GS^-], mol L^{-1}	[OH^-], mol L^{-1}	k_{obs}, s^{-1}
4.38×10^{-6}	1.00×10^{-4}	1.00×10^{-2}	1.00×10^{-4}	9.70×10^{-3}	2.47×10^{2}
4.38×10^{-6}	2.00×10^{-4}	1.00×10^{-2}	2.00×10^{-4}	9.40×10^{-3}	5.04×10^{2}
4.38×10^{-6}	3.00×10^{-4}	1.00×10^{-2}	3.00×10^{-4}	9.10×10^{-3}	8.83×10^{2}
4.38×10^{-6}	4.00×10^{-4}	1.00×10^{-2}	4.00×10^{-4}	8.80×10^{-3}	1.02×10^{3}

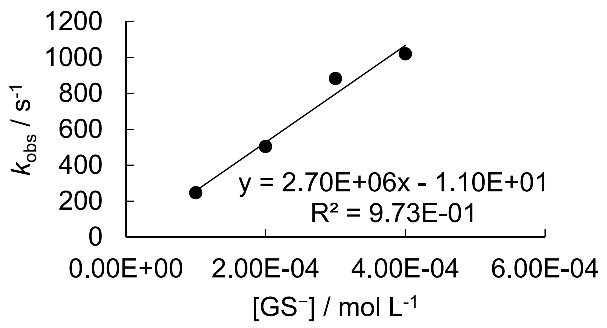

$k_2 = 2.70 \times 10^6$ M^{-1} s^{-1}

E3 + GSH in alkaline 99.5/0.5 (v/v) H_2O/CH_3CN (stopped-flow, detection at 634 nm)

[**E3**]$_0$, mol L^{-1}	[GSH]$_0$, mol L^{-1}	[OH^-]$_0$, mol L^{-1}	[GS^-], mol L^{-1}	[OH^-], mol L^{-1}	k_{obs}, s^{-1}
2.32×10^{-6}	1.00×10^{-4}	1.00×10^{-2}	1.00×10^{-4}	9.70×10^{-3}	8.41×10^{1}
2.32×10^{-6}	2.00×10^{-4}	1.00×10^{-2}	2.00×10^{-4}	9.40×10^{-3}	2.07×10^{2}
2.32×10^{-6}	3.00×10^{-4}	1.00×10^{-2}	3.00×10^{-4}	9.10×10^{-3}	3.27×10^{2}
2.32×10^{-6}	4.00×10^{-4}	1.00×10^{-2}	4.00×10^{-4}	8.80×10^{-3}	5.00×10^{2}

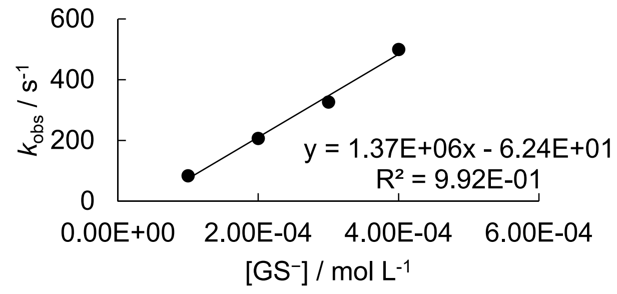

$k_2 = 1.37 \times 10^6$ M^{-1} s^{-1}

E4 + GSH in alkaline 99.5/0.5 (v/v) H_2O/CH_3CN (stopped-flow, detection at 630 nm)

[**E4**]$_0$, mol L^{-1}	[GSH]$_0$, mol L^{-1}	[OH$^-$]$_0$, mol L^{-1}	[GS$^-$], mol L^{-1}	[OH$^-$], mol L^{-1}	k_{obs}, s^{-1}
5.45×10^{-6}	1.00×10^{-4}	1.00×10^{-2}	1.00×10^{-4}	9.70×10^{-3}	6.69×10^{1}
5.45×10^{-6}	2.00×10^{-4}	1.00×10^{-2}	2.00×10^{-4}	9.40×10^{-3}	1.43×10^{2}
5.45×10^{-6}	3.00×10^{-4}	1.00×10^{-2}	3.00×10^{-4}	9.10×10^{-3}	2.06×10^{2}
5.45×10^{-6}	4.00×10^{-4}	1.00×10^{-2}	4.00×10^{-4}	8.80×10^{-3}	2.66×10^{2}

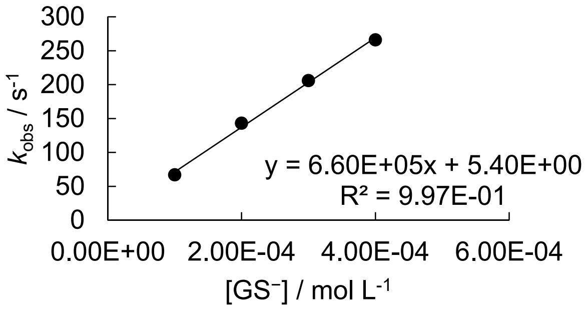

$k_2 = 6.60 \times 10^{5}$ M^{-1} s^{-1}

E5 + GSH in alkaline 89/11 (v/v) H_2O/CH_3CN (stopped-flow, detection at 396 nm)

[**E5**]$_0$, mol L^{-1}	[GSH]$_0$, mol L^{-1}	[OH$^-$]$_0$, mol L^{-1}	[GS$^-$], mol L^{-1}	[OH$^-$], mol L^{-1}	k_{obs}, s^{-1}
1.03×10^{-5}	1.00×10^{-4}	1.00×10^{-2}	1.00×10^{-4}	9.70×10^{-3}	9.01×10^{1}
1.03×10^{-5}	2.00×10^{-4}	1.00×10^{-2}	2.00×10^{-4}	9.40×10^{-3}	1.79×10^{2}
1.03×10^{-5}	3.00×10^{-4}	1.00×10^{-2}	3.00×10^{-4}	9.10×10^{-3}	2.76×10^{2}
1.03×10^{-5}	4.00×10^{-4}	1.00×10^{-2}	4.00×10^{-4}	8.80×10^{-3}	3.78×10^{2}

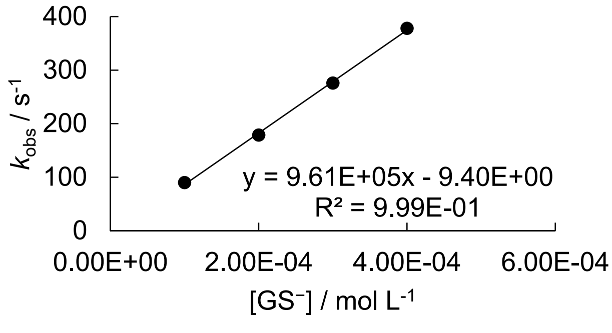

$k_2 = 9.61 \times 10^{5}$ M^{-1} s^{-1}

E6 + GSH in alkaline 89/11 (v/v) H_2O/CH_3CN (stopped-flow, detection at 504 nm)

[**E6**]$_0$, mol L^{-1}	[GSH]$_0$, mol L^{-1}	[OH^-]$_0$, mol L^{-1}	[GS^-], mol L^{-1}	[OH^-], mol L^{-1}	k_{obs}, s^{-1}
1.21×10^{-5}	1.00×10^{-4}	1.00×10^{-2}	1.00×10^{-4}	9.70×10^{-3}	1.14×10^{1}
1.21×10^{-5}	2.00×10^{-4}	1.00×10^{-2}	2.00×10^{-4}	9.40×10^{-3}	1.83×10^{1}
1.21×10^{-5}	3.00×10^{-4}	1.00×10^{-2}	3.00×10^{-4}	9.10×10^{-3}	2.62×10^{1}
1.21×10^{-5}	4.00×10^{-4}	1.00×10^{-2}	4.00×10^{-4}	8.80×10^{-3}	3.41×10^{1}

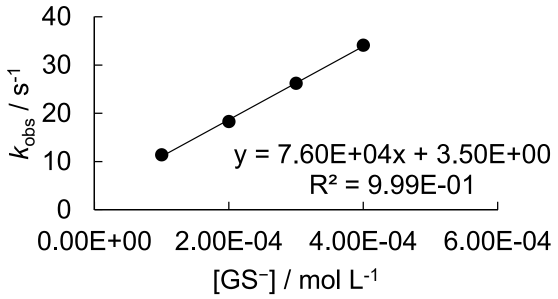

$k_2 = 7.60 \times 10^4$ M^{-1} s^{-1}

E7 + GSH in alkaline 99.5/0.5 (v/v) H_2O/CH_3CN (stopped-flow, detection at 310 nm)

[**E7**]$_0$, mol L^{-1}	[GSH]$_0$, mol L^{-1}	[OH^-]$_0$, mol L^{-1}	[GS^-], mol L^{-1}	[OH^-], mol L^{-1}	k_{obs}, s^{-1}
1.90×10^{-5}	1.00×10^{-4}	1.00×10^{-2}	1.00×10^{-4}	9.70×10^{-3}	1.50×10^{1}
1.90×10^{-5}	2.00×10^{-4}	1.00×10^{-2}	2.00×10^{-4}	9.40×10^{-3}	3.01×10^{1}
1.90×10^{-5}	3.00×10^{-4}	1.00×10^{-2}	3.00×10^{-4}	9.10×10^{-3}	4.65×10^{1}
1.90×10^{-5}	4.00×10^{-4}	1.00×10^{-2}	4.00×10^{-4}	8.80×10^{-3}	5.81×10^{1}

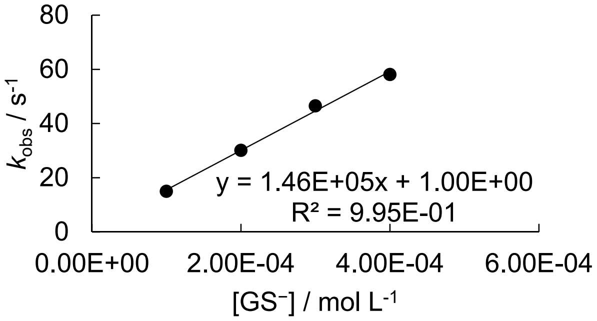

$k_2 = 1.46 \times 10^5$ M^{-1} s^{-1}

E8 + GSH in alkaline 99.5/0.5 (v/v) H_2O/CH_3CN (stopped-flow, detection at 362 nm)

[**E8**]$_0$, mol L^{-1}	[GSH]$_0$, mol L^{-1}	[OH$^-$]$_0$, mol L^{-1}	[GS$^-$], mol L^{-1}	[OH$^-$], mol L^{-1}	k_{obs}, s^{-1}
1.96×10^{-5}	1.00×10^{-4}	1.00×10^{-2}	1.00×10^{-4}	9.70×10^{-3}	3.40
1.96×10^{-5}	2.00×10^{-4}	1.00×10^{-2}	2.00×10^{-4}	9.40×10^{-3}	6.83
1.96×10^{-5}	3.00×10^{-4}	1.00×10^{-2}	3.00×10^{-4}	9.10×10^{-3}	1.06×10^{1}
1.96×10^{-5}	4.00×10^{-4}	1.00×10^{-2}	4.00×10^{-4}	8.80×10^{-3}	1.42×10^{1}

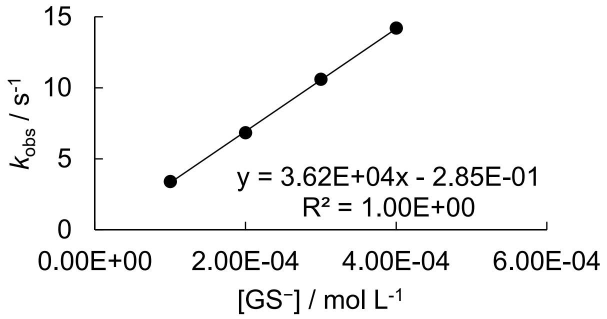

$k_2 = 3.62 \times 10^4$ M^{-1} s^{-1}

E9 + GSH in alkaline 89/11 (v/v) H_2O/CH_3CN (stopped-flow, detection at 487 nm)

[**E9**]$_0$, mol L^{-1}	[GSH]$_0$, mol L^{-1}	[OH$^-$]$_0$, mol L^{-1}	[GS$^-$], mol L^{-1}	[OH$^-$], mol L^{-1}	k_{obs}, s^{-1}
3.27×10^{-5}	1.00×10^{-4}	1.00×10^{-2}	1.00×10^{-4}	9.70×10^{-3}	2.42×10^{1}
3.27×10^{-5}	2.00×10^{-4}	1.00×10^{-2}	2.00×10^{-4}	9.40×10^{-3}	5.16×10^{1}
3.27×10^{-5}	3.00×10^{-4}	1.00×10^{-2}	3.00×10^{-4}	9.10×10^{-3}	7.65×10^{1}
3.27×10^{-5}	4.00×10^{-4}	1.00×10^{-2}	4.00×10^{-4}	8.80×10^{-3}	1.02×10^{2}

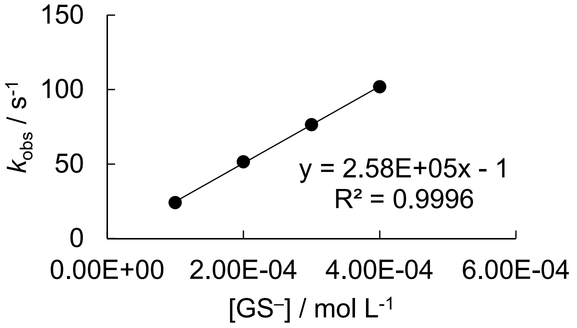

$k_2 = 2.58 \times 10^5$ M^{-1} s^{-1}

E10 + GSH in alkaline 89/11 (v/v) H_2O/CH_3CN (stopped-flow, detection at 512 nm)

[**E10**]$_0$, mol L^{-1}	[GSH]$_0$, mol L^{-1}	[OH$^-$]$_0$, mol L^{-1}	[GS$^-$], mol L^{-1}	[OH$^-$], mol L^{-1}	k_{obs}, s^{-1}
3.36×10^{-5}	1.00×10^{-4}	1.00×10^{-2}	1.00×10^{-4}	9.70×10^{-3}	5.19
3.36×10^{-5}	2.00×10^{-4}	1.00×10^{-2}	2.00×10^{-4}	9.40×10^{-3}	1.09×10^{1}
3.36×10^{-5}	3.00×10^{-4}	1.00×10^{-2}	3.00×10^{-4}	9.10×10^{-3}	1.64×10^{1}
3.36×10^{-5}	4.00×10^{-4}	1.00×10^{-2}	4.00×10^{-4}	8.80×10^{-3}	2.20×10^{1}
3.36×10^{-5}	5.00×10^{-4}	1.00×10^{-2}	5.00×10^{-4}	8.50×10^{-3}	2.62×10^{1}

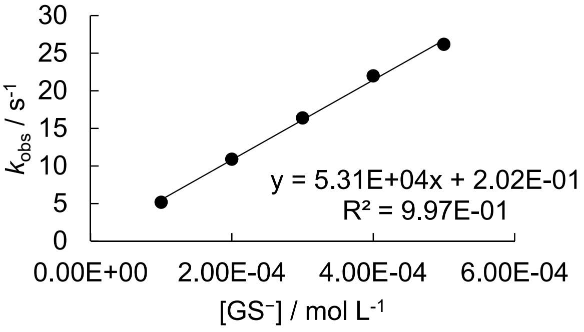

$k_2 = 5.31 \times 10^4$ M^{-1} s^{-1}

Kinetics of AcCys with cationic and neutral reference electrophiles

E2 + AcCys in alkaline 99.5/0.5 (v/v) H_2O/CH_3CN (stopped-flow, detection at 614 nm)

[**E2**]$_0$, mol L^{-1}	[AcCys]$_0$, mol L^{-1}	[OH$^-$]$_0$, mol L^{-1}	[AcCys$^-$], mol L^{-1}	[OH$^-$], mol L^{-1}	k_{obs}, s^{-1}
3.06×10^{-6}	1.00×10^{-4}	1.00×10^{-2}	9.96×10^{-5}	9.80×10^{-3}	1.18×10^{2}
3.06×10^{-6}	2.00×10^{-4}	1.00×10^{-2}	1.99×10^{-4}	9.60×10^{-3}	2.58×10^{2}
3.06×10^{-6}	3.00×10^{-4}	1.00×10^{-2}	2.99×10^{-4}	9.40×10^{-3}	4.16×10^{2}
3.06×10^{-6}	4.00×10^{-4}	1.00×10^{-2}	3.98×10^{-4}	9.20×10^{-3}	5.64×10^{2}
3.06×10^{-6}	5.00×10^{-4}	1.00×10^{-2}	4.98×10^{-4}	9.00×10^{-3}	6.97×10^{2}

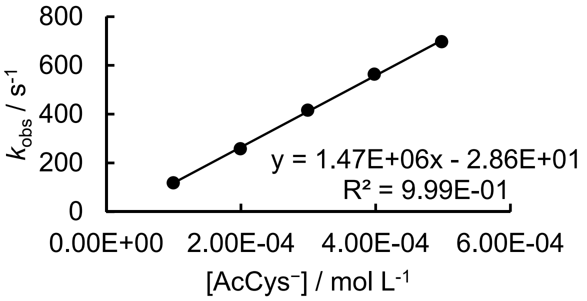

$k_2 = 1.47 \times 10^{6}$ M^{-1} s^{-1}

E3 + AcCys in alkaline 99.5/0.5 (v/v) H_2O/CH_3CN (stopped-flow, detection at 634 nm)

[**E3**]$_0$, mol L^{-1}	[AcCys]$_0$, mol L^{-1}	[OH$^-$]$_0$, mol L^{-1}	[AcCys$^-$], mol L^{-1}	[OH$^-$], mol L^{-1}	k_{obs}, s^{-1}
2.88×10^{-6}	1.00×10^{-4}	1.00×10^{-2}	9.96×10^{-5}	9.80×10^{-3}	4.62×10^{1}
2.88×10^{-6}	2.00×10^{-4}	1.00×10^{-2}	1.99×10^{-4}	9.60×10^{-3}	9.28×10^{1}
2.88×10^{-6}	3.00×10^{-4}	1.00×10^{-2}	2.99×10^{-4}	9.40×10^{-3}	1.54×10^{2}
2.88×10^{-6}	4.00×10^{-4}	1.00×10^{-2}	3.98×10^{-4}	9.20×10^{-3}	2.04×10^{2}
2.88×10^{-6}	5.00×10^{-4}	1.00×10^{-2}	4.98×10^{-4}	9.00×10^{-3}	2.68×10^{2}

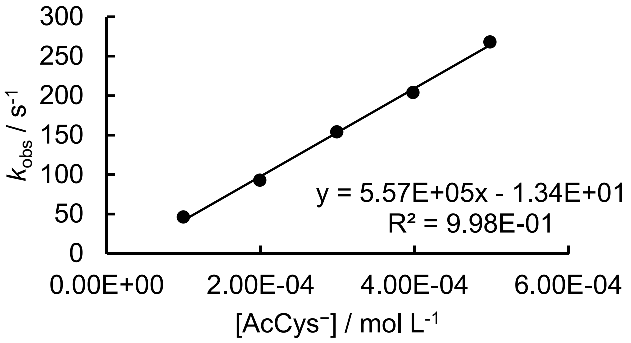

$k_2 = 5.57 \times 10^{5}$ M^{-1} s^{-1}

E4 + AcCys in alkaline 99.5/0.5 (v/v) H_2O/CH_3CN (stopped-flow, detection at 630 nm)

[**E4**]$_0$, mol L^{-1}	[AcCys]$_0$, mol L^{-1}	[OH$^-$]$_0$, mol L^{-1}	[AcCys$^-$], mol L^{-1}	[OH$^-$], mol L^{-1}	k_{obs}, s^{-1}
3.24×10^{-6}	1.00×10^{-4}	1.00×10^{-2}	9.96×10^{-5}	9.80×10^{-3}	3.31×10^{1}
3.24×10^{-6}	2.00×10^{-4}	1.00×10^{-2}	1.99×10^{-4}	9.60×10^{-3}	6.50×10^{1}
3.24×10^{-6}	3.00×10^{-4}	1.00×10^{-2}	2.99×10^{-4}	9.40×10^{-3}	1.02×10^{2}
3.24×10^{-6}	4.00×10^{-4}	1.00×10^{-2}	3.98×10^{-4}	9.20×10^{-3}	1.37×10^{2}
3.24×10^{-6}	5.00×10^{-4}	1.00×10^{-2}	4.98×10^{-4}	9.00×10^{-3}	1.70×10^{2}

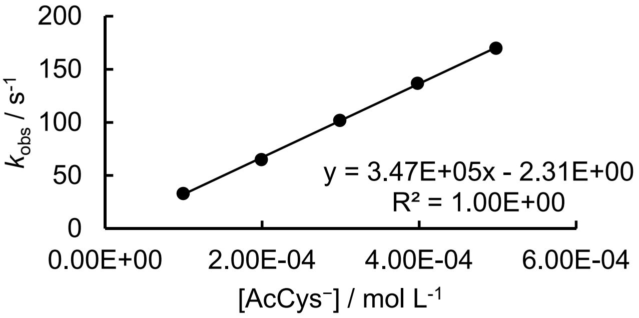

$k_2 = 3.47 \times 10^5$ M^{-1} s^{-1}

E5 + AcCys in alkaline 89/11 (v/v) H_2O/CH_3CN (stopped-flow, detection at 396 nm)

[**E5**]$_0$, mol L^{-1}	[AcCys]$_0$, mol L^{-1}	[OH$^-$]$_0$, mol L^{-1}	[AcCys$^-$], mol L^{-1}	[OH$^-$], mol L^{-1}	k_{obs}, s^{-1}
3.09×10^{-5}	1.00×10^{-4}	1.00×10^{-2}	9.96×10^{-5}	9.80×10^{-3}	5.32×10^{1}
3.09×10^{-5}	2.00×10^{-4}	1.00×10^{-2}	1.99×10^{-4}	9.60×10^{-3}	1.08×10^{2}
3.09×10^{-5}	3.00×10^{-4}	1.00×10^{-2}	2.99×10^{-4}	9.40×10^{-3}	1.55×10^{2}
3.09×10^{-5}	4.00×10^{-4}	1.00×10^{-2}	3.98×10^{-4}	9.20×10^{-3}	2.13×10^{2}
3.09×10^{-5}	5.00×10^{-4}	1.00×10^{-2}	4.98×10^{-4}	9.00×10^{-3}	2.60×10^{2}

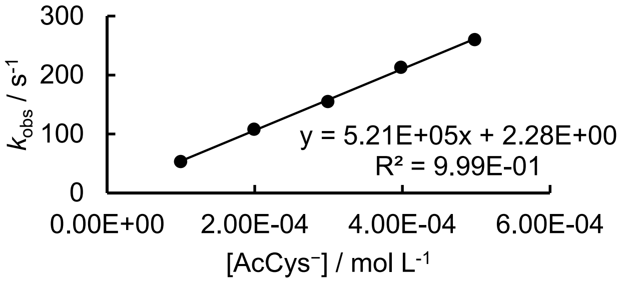

$k_2 = 5.21 \times 10^5$ M^{-1} s^{-1}

E8 + AcCys in alkaline 99.5/0.5 (v/v) H_2O/CH_3CN (stopped-flow, detection at 362 nm)

[**E8**]$_0$, mol L^{-1}	[AcCys]$_0$, mol L^{-1}	[OH$^-$]$_0$, mol L^{-1}	[AcCys$^-$], mol L^{-1}	[OH$^-$], mol L^{-1}	k_{obs}, s^{-1}
4.30×10^{-5}	1.00×10^{-4}	1.00×10^{-2}	9.96×10^{-5}	9.80×10^{-3}	2.60
4.30×10^{-5}	2.00×10^{-4}	1.00×10^{-2}	1.99×10^{-4}	9.60×10^{-3}	5.93
4.30×10^{-5}	3.00×10^{-4}	1.00×10^{-2}	2.99×10^{-4}	9.40×10^{-3}	8.88
4.30×10^{-5}	4.00×10^{-4}	1.00×10^{-2}	3.98×10^{-4}	9.20×10^{-3}	1.22×10^{1}
4.30×10^{-5}	5.00×10^{-4}	1.00×10^{-2}	4.98×10^{-4}	9.00×10^{-3}	1.51×10^{1}

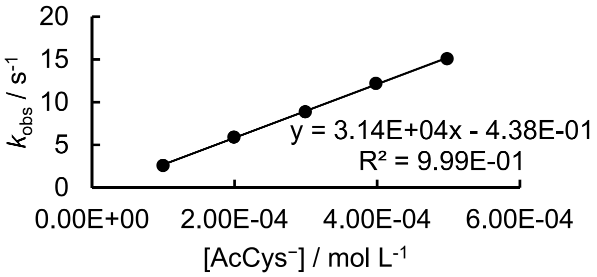

$k_2 = 3.14 \times 10^4$ M^{-1} s^{-1}

E9 + AcCys in alkaline 89/11 (v/v) H_2O/CH_3CN (stopped-flow, detection at 487 nm)

[**E9**]$_0$, mol L^{-1}	[AcCys]$_0$, mol L^{-1}	[OH$^-$]$_0$, mol L^{-1}	[AcCys$^-$], mol L^{-1}	[OH$^-$], mol L^{-1}	k_{obs}, s^{-1}
3.27×10^{-5}	1.00×10^{-4}	1.00×10^{-2}	9.96×10^{-5}	9.80×10^{-3}	2.00×10^{1}
3.27×10^{-5}	2.00×10^{-4}	1.00×10^{-2}	1.99×10^{-4}	9.60×10^{-3}	4.03×10^{1}
3.27×10^{-5}	3.00×10^{-4}	1.00×10^{-2}	2.99×10^{-4}	9.40×10^{-3}	5.96×10^{1}
3.27×10^{-5}	4.00×10^{-4}	1.00×10^{-2}	3.98×10^{-4}	9.20×10^{-3}	7.94×10^{1}
3.27×10^{-5}	5.00×10^{-4}	1.00×10^{-2}	4.98×10^{-4}	9.00×10^{-3}	9.80×10^{1}

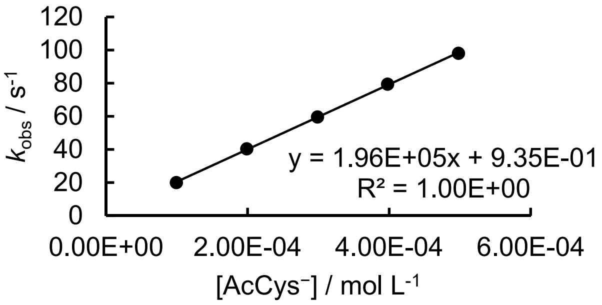

$k_2 = 1.96 \times 10^5$ M^{-1} s^{-1}

E10 + AcCys in alkaline 89/11 (v/v) H_2O/CH_3CN (stopped-flow, detection at 512 nm)

[**E10**]$_0$, mol L$^{–1}$	[AcCys]$_0$, mol L$^{–1}$	[OH$^–$]$_0$, mol L$^{–1}$	[AcCys$^–$], mol L$^{–1}$	[OH$^–$], mol L$^{–1}$	k_{obs}, s$^{–1}$
3.36×10^{-5}	1.00×10^{-4}	1.00×10^{-2}	9.96×10^{-5}	9.80×10^{-3}	4.35
3.36×10^{-5}	2.00×10^{-4}	1.00×10^{-2}	1.99×10^{-4}	9.60×10^{-3}	8.85×10^{0}
3.36×10^{-5}	3.00×10^{-4}	1.00×10^{-2}	2.99×10^{-4}	9.40×10^{-3}	1.28×10^{1}
3.36×10^{-5}	4.00×10^{-4}	1.00×10^{-2}	3.98×10^{-4}	9.20×10^{-3}	1.70×10^{1}
3.36×10^{-5}	5.00×10^{-4}	1.00×10^{-2}	4.98×10^{-4}	9.00×10^{-3}	2.08×10^{1}

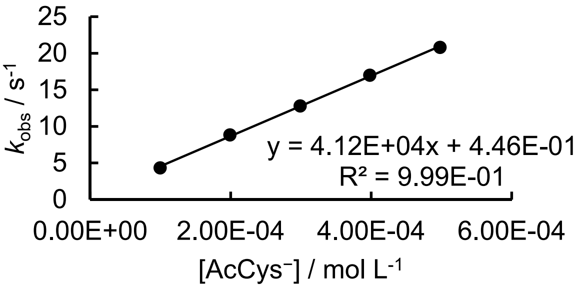

$k_2 = 4.12 \times 10^4$ M$^{–1}$ s$^{–1}$

Electrophilicity of Michael Acceptors towards GSH in Kinetic Assays

Table S4: Rate constants for reactions of Michael acceptors with GSH from kinetic assays.

Label	Michael acceptor	k_{GSH} ($M^{-1}\ s^{-1}$)	pH (*T*)	$k_2(GS^-)$[a] ($M^{-1}\ s^{-1}$)	Estimated *E*[b]	ΔG_{MA}[c] (kJ mol^{-1})
	**α,β-unsaturated aldehydes**					
**M1**	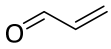<br>acrolein	1.21×10^2<br>1.20×10^2<br>7.28	7.4 (20 °C)[d]<br>7.2 (25 °C)[e]<br>8.0 (25 °C)[f]	4.32×10^3	−14.4	−119.0
**M2**	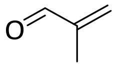<br>methacrolein	3.38	7.4 (25 °C)[g]	121	−17.2	−94.2
**M3**	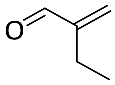<br>2-ethyl acrolein	9.90×10^{-1}	7.4 (25 °C)[g]	35.4	−18.2	−93.2
**M4**	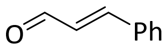<br>cinnamaldehyde	9.15×10^{-2}	7.4 (25 °C)[g]	3.27	−20.0	−94.9
**M5**	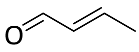<br>crotonaldehyde	7.85×10^{-1}<br>7.2×10^{-1}<br>2.67	7.4 (20 °C)[d]<br>7.2 (25 °C)[e]<br>8.0 (25 °C)[f]	28.0	−18.4	−91.6
**M6**	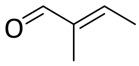<br>trans-2-methyl-2-butenal	7.90×10^{-3}	7.4 (25 °C)[g]	2.82×10^{-1}	−22.0	−66.8
**M7**	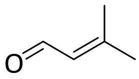<br>3-methyl-2-butenal	2.85×10^{-2}	7.4 (25 °C)[g]	1.02	−21.0	−68.8
**M8**	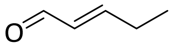<br>2-pentenal	4.71×10^{-1}<br>4.72×10^{-1}<br>1.5	7.4 (20 °C)[d]<br>7.4 (25 °C)[g]<br>8.0 (25 °C)[f]	16.8	−18.8	−87.3
**M9**	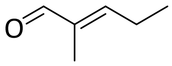<br>trans-2-methyl-2-pentenal	4.57×10^{-3}	7.4 (25 °C)[g]	1.63×10^{-1}	−22.4	−62.5
**M10**	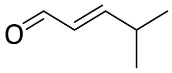<br>4-methyl-2-pentenal	1.77×10^{-1}	7.4 (25 °C)[g]	6.31	−19.5	−78.0
**M11**	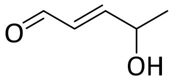<br>4-hydroxy-2-pentenal	2.19	7.4 (20 °C)[d]	78.2	−17.6	−115.2

**M12**	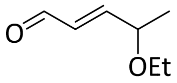<br>4-ethoxy-2-pentenal	1.83	7.4 (20 °C)[d]	65.4	−17.7	−85.0
**M13**	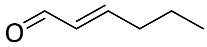<br>2-hexenal	3.30×10^{-1}<br>4.18×10^{-1}<br>3.40×10^{-1}<br>9.0×10^{-1}	7.4 (20 °C)[d]<br>7.4 (25 °C)[g]<br>7.2 (25 °C)[e]<br>8.0 (25 °C)[f]	11.8	−19.0	−85.7
**M14**	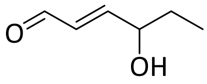<br>4-hydroxy-2-hexenal	1.56	7.4 (20 °C)[d]	55.7	−17.8	−109.7
**M15**	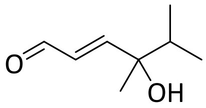<br>(E)-4-hydroxy-4,5-dimethylhex-2-enal	3.57×10^{-2}	7.4 (20 °C)[d]	1.28	−20.8	−93.4
**M16**	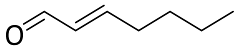<br>2-heptenal	9.3×10^{-1}	8.0 (25 °C)[f]	9.0	−19.2	−85.2
**M17**	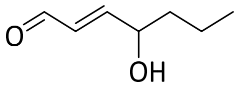<br>4-hydroxy-2-heptenal	1.83	7.4 (20 °C)[d]	65.4	−17.7	−109.4
**M18**	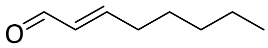<br>trans-2-octenal	3.00×10^{-1}<br>2.8×10^{-1}<br>7.2×10^{-1}	7.4 (25 °C)[g]<br>7.2 (25 °C)[e]<br>8.0 (25 °C)[f]	10.7	−19.1	−85.1
**M19**	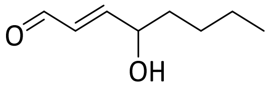<br>4-hydroxy-2-octenal	1.74	7.4 (20 °C)[d]	62.1	−17.7	−109.0
**M20**	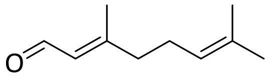<br>citral	3.23×10^{-2}<br>3.3×10^{-2}	7.4 (20 °C)[d]<br>8.0 (25 °C)[f]	1.15	−20.9	−59.5
**M21**	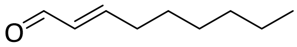<br>trans-2-nonenal	2.52×10^{-1}<br>6.5×10^{-1}	7.4 (25 °C)[g]<br>8.0 (25 °C)[f]	8.99	−19.3	−81.5
**M22**	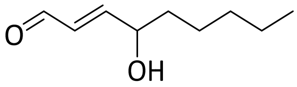<br>4-hydroxy-2-nonenal	1.09	7.4 (20 °C)[d]	38.9	−18.1	−112.3
**M23**	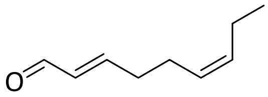<br>trans-2-cis-6-nonadienal	3.80×10^{-1}<br>5.7×10^{-1}<br>1.1	7.4 (25 °C)[g]<br>7.2 (25 °C)[e]<br>8.0 (25 °C)[f]	13.6	−18.9	−87.3
**M24**	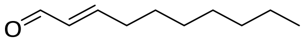<br>trans-2-decenal	1.68×10^{-1}	7.4 (25 °C)[g]	6.01	−19.6	−85.1

**M25**	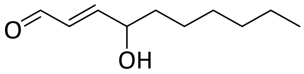 4-hydroxy-2-decenal	1.96	7.4 (20 °C)[d]	70.0	–17.6	–107.1
**M26**	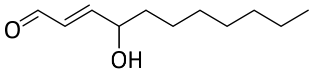 4-hydroxy-2-undecenal	1.47	7.4 (20 °C)[d]	52.5	–17.9	–109.7
**M27**	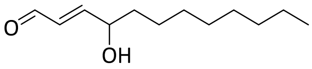 4-hydroxy-2-dodecenal	2.44	7.4 (20 °C)[d]	87.1	–17.5	–106.8
**M28**	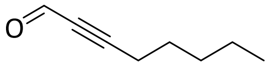 2-octynal	8.12	7.4 (25 °C)[g]	290	–16.5	–111.0
**M29**	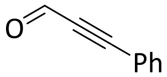 phenyl propiolaldehyde	7.43	7.4 (25 °C)[g]	265	–16.6	–121.0
	**α,β-unsaturated ketones**					
**E11**	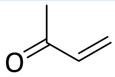 methyl vinylketone	3.19×10^{1}	7.4 (20 °C)[d]	1.14×10^{3}	–16.8[h]	–104.2[h]
**M30**	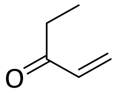 1-penten-3-one	2.10×10^{1}	7.4 (25 °C)[g]	751	–15.8	–99.3
**M31**	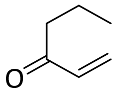 1-hexen-3-one	1.96×10^{1}	7.4 (25 °C)[g]	698	–15.8	–99.7
**M32**	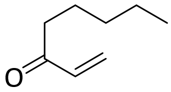 1-octen-3-one	1.79×10^{1}	7.4 (25 °C)[g]	639	–15.9	–99.0
**M33**	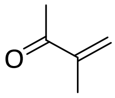 3-methyl-3-buten-2-one	6.00×10^{-1}	7.4 (20 °C)[d]	21.4	–18.6	–72.6
**M34**	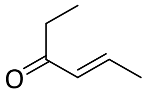 4-hexen-3-one	4.03×10^{-1}	7.4 (25 °C)[g]	14.4	–18.9	–74.9
**M35**	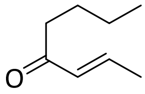 2-octen-4-one	4.35×10^{-1}	7.4 (25 °C)[g]	15.5	–18.8	–74.8
**M36**	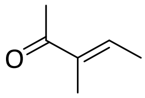 3-methyl-3-penten-2-one	1.30×10^{-2}	7.4 (25 °C)[g]	4.64×10^{-1}	–21.6	–51.0
**M37**	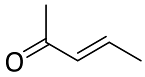 3-penten-2-one	4.45×10^{-1}	7.4 (25 °C)[g]	15.9	–18.8	–79.5
**M38**	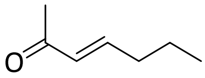	2.08×10^{-1}	7.4 (25 °C)[g]	7.44	–19.4	–73.7

	3-hepten-2-one					
**M39**	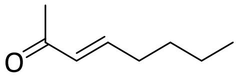 3-octen-2-one	1.90×10^{-1}	7.4 (25 °C)[g]	6.79	−19.5	−73.5
**M40**	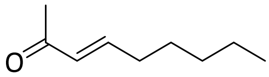 3-nonen-2-one	1.80×10^{-1}	7.4 (25 °C)[g]	6.43	−19.5	−75.4
**M41**	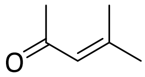 mesityl oxide	2.30×10^{-3}<br>3.47×10^{-3}	7.4 (20 °C)[d]<br>7.4 (25 °C)[g]	8.21×10^{-2}	−22.9	−58.6
**M42**	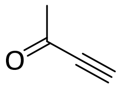 3-butyn-2-one	7.62	7.4 (25 °C)[i]	272	−16.6	−139.5
**M43**	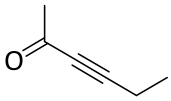 3-hexyn-2-one	1.33	7.4 (25 °C)[g]	47.6	−17.9	−92.9
**M44**	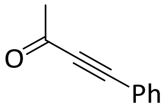 4-phenyl-3-butyn-2-one	5.03	7.4 (25 °C)[i]	180	−16.9	−101.5
	**α,β-unsaturated esters**					
**M45**	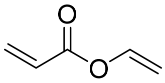 vinyl acrylate	1.49	7.4 (25 °C)[i]	53.1	−17.9	−116.4
**M46**	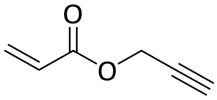 propargyl acrylate	8.57×10^{-1}	7.4 (25 °C)[g]	30.6	−18.3	−94.2
**M47**	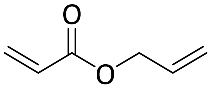 allyl acrylate	3.27×10^{-1}	7.4 (25 °C)[g]	11.7	−19.0	−83.6
**M48**	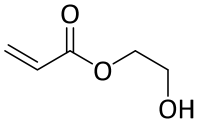 2-hydroxyethyl acrylate	3.32×10^{-1}	7.4 (25 °C)[g]	11.8	−19.0	−100.3
**M49**	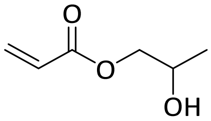 2-hydroxypropyl acrylate	3.00×10^{-1}	7.4 (25 °C)[g]	10.7	−19.1	−98.6
**E12**	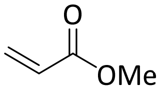 methyl acrylate	1.90×10^{-1}	7.4 (25 °C)[g]	6.79	−18.8[h]	−80.7[h]
**E13**	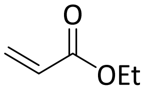 ethyl acrylate	1.77×10^{-1}	7.4 (25 °C)[g]	6.31	−19.1[h]	−75.1[h]

**M50**	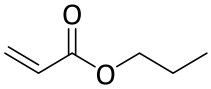 n-propyl acrylate	1.70 × 10^{-1}	7.4 (25 °C)[g]	6.07	−19.6	−75.1
**M51**	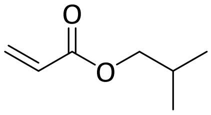 iso-butyl acrylate	1.57 × 10^{-1}	7.4 (25 °C)[g]	5.60	−19.6	−77.3
**M52**	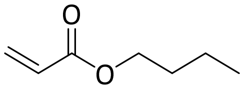 n-butyl acrylate	1.42 × 10^{-1}	7.4 (25 °C)[g]	5.08	−19.7	−74.3
**M53**	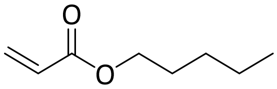 hexyl acrylate	1.32 × 10^{-1}	7.4 (25 °C)[i]	4.73	−19.8	−74.1
**E14**	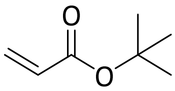 tert-butyl acrylate	4.17 × 10^{-2}	7.4 (25 °C)[g]	1.49	−20.2[h]	−71.6[h]
**M54**	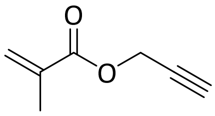 propargyl methacrylate	3.67 × 10^{-3}	7.4 (25 °C)[g]	1.31 × 10^{-1}	−22.6	−70.4
**M55**	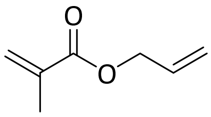 allyl methacrylate	1.52 × 10^{-2}	7.4 (25 °C)[i]	5.43 × 10^{-1}	−21.4	−58.2
**M56**	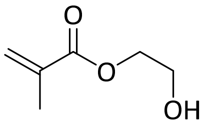 2-hydroxyethyl methacrylate	2.90 × 10^{-3}	7.4 (25 °C)[i]	1.03 × 10^{-1}	−22.7	−78.8
**M57**	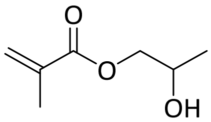 2-hydroxypropyl methacrylate	1.56 × 10^{-2}	7.4 (25 °C)[i]	5.56 × 10^{-1}	−21.4	−81.2
**M58**	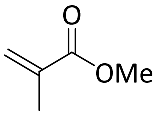 methyl methacrylate	1.20 × 10^{-3}	7.4 (25 °C)[g]	4.29 × 10^{-2}	−23.4	−57.0
**E16**	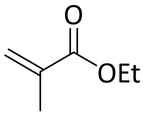 ethyl methacrylate	9.67 × 10^{-4}	7.4 (25 °C)[g]	3.45 × 10^{-2}	−22.8[h]	−51.9[h]

**M59**	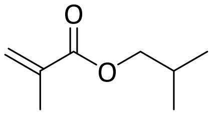 isobutyl methacrylate	6.05×10^{-3}	7.4 (25 °C)[i]	2.16×10^{-1}	−22.2	−51.0
**M60**	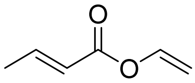 vinyl crotonate	2.30×10^{-2}	7.4 (25 °C)[i]	8.22×10^{-1}	−21.1	−90.7
**M61**	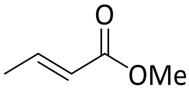 methyl crotonate	2.73×10^{-3}	7.4 (25 °C)[g]	9.76×10^{-2}	−22.8	−55.8
**E17**	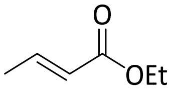 ethyl crotonate	3.10×10^{-3}<br>2.68×10^{-3}	7.4 (20 °C)[d]<br>7.4 (25 °C)[g]	1.11×10^{-1}	−23.6[h]	−49.8[h]
**M62**	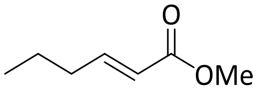 methyl trans-2-hexenoate	3.63×10^{-2}	7.4 (25 °C)[g]	1.30	−20.8	−50.5
**M63**	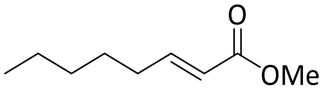 methyl trans-2-octenoate	1.31×10^{-2}	7.4 (25 °C)[g]	4.67×10^{-1}	−21.6	−50.2
**M64**	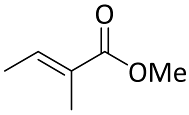 methyl tiglate	1.17×10^{-4}	7.4 (25 °C)[g]	4.17×10^{-3}	−25.3	−41.4
**M65**	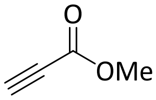 methyl propiolate	1.95	7.4 (25 °C)[g]	69.6	−17.6	−137.3
**M66**	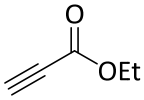 ethyl propiolate	1.75	7.4 (25 °C)[g]	62.5	−17.7	−133.9
**M67**	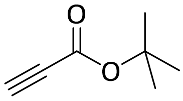 tert-butyl propiolate	1.04	7.4 (25 °C)[g]	37.3	−18.1	−121.7
**M68**	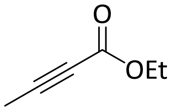 ethyl 2-butynoate	4.03×10^{-2}	7.4 (25 °C)[g]	1.44	−20.7	−88.8
**M69**	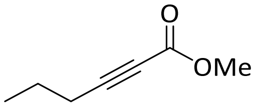 methyl 2-hexynoate	9.50×10^{-2}	7.4 (25 °C)[g]	3.39	−20.0	−87.4
**M70**	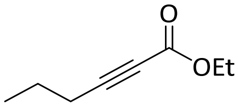 ethyl hex-2-ynoate	6.95×10^{-2}	7.4 (25 °C)[i]	2.48	−20.3	−87.3

**M71**	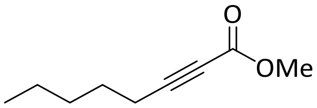 methyl 2-octynoate	4.60 × 10^{-2}	7.4 (25 °C)[g]	1.64	–20.6	–89.6
	**further Michael acceptors**					
**M72**	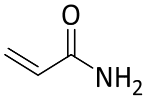 acrylamide	9.27 × 10^{-3}	7.4 (25 °C)[g]	3.31 × 10^{-1}	–21.8	–66.5
**E15**	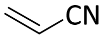 acrylonitrile	1.73 × 10^{-1}	8.1 (30 °C)[j]	1.36	–19.1[h]	–109.4[h]
**M73**	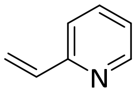 2-vinylpyridine	3.48 × 10^{-2}	7.4 (25 °C)[i]	1.24	–20.8	–62.8

[a] Calculated by using Equation 2 (main text). [b] Calculated by using equation 1 (main text), lg k_2(GS$^-$), and the reactivity parameters for GS$^-$ (N = 20.97, s_N = 0.55$_5$). [c] For details of the quantum-chemical calculation of methyl anion affinities ΔG_{MA} see Table S5. [d] From ref [S7a]. [e] From ref [S7b]. [f] From ref [S7c]. [g] From ref [S8]. [h] From ref [S10c]. [i] From ref [S9]. [j] From ref [S2].

Quantum-Chemically Calculated Methyl Anion Affinities (ΔG_{MA}) of Michael Acceptors

Gas phase methyl anion affinities ΔG_{MA} were calculated as described in ref[S10] as negative Gibbs free energies at 298.15 K for the reactions of Michael acceptors (from Table S4) with the methyl anion:

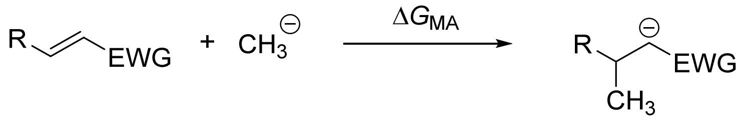

All structures were subjected to a conformational search employing the OPLS-3[S11] force field in gas phase with the MCMM method as implemented in Macromodel 2019-1.[S12] The 10 conformers with the lowest OPLS-3 energy for every system were subsequently optimized at the B3LYP/6-31G(d,p)[S13–S15] level in gas phase. For the optimized structures, single-point gas-phase energies with the B3LYP/6-311++G(3df,2pd)[S13–S16] method and single-point solvation energies at the B3LYP/6-31G(d,p) level employing the SMD model for DMSO[S17] were calculated. Final Gibbs free energies were obtained by combining the single point energies at the B3LYP/6-311++G(3df,2pd) with the thermochemical corrections at the B3LYP/6-31G(d,p) level and single point solvation energies at the B3LYP/6-31G(d,p) level. Methyl anion affinities were then determined for every electrophile by calculating the reaction energy for the lowest lying conformer. The quantum-mechanical calculations were performed with Gaussian 16, Rev. A.03.[S18]

Table S5: Quantum-mechanically calculated methyl anion affinities ΔG_{MA} for the 1,4-addition to Michael acceptors [in kJ mol^{-1}, calculated at the SMD(DMSO)/B3LYP/6-311++G(3df,2pd)//B3LYP/6-31G(d,p) level of theory]

Michael acceptor	Filename	E_{tot} (B3LYP/6-31G(d,p)	G_{298}	$E_{tot, solv.}$ (SMD(DMSO)/B3LYP/6-31G(d,p)// B3LYP/6-31G(d,p))	$E_{tot, high.}$ (B3LYP/6-311++G(3df,2pd)// B3LYP/6-31G(d,p))	ΔG	ΔG_{solv}	G_{298} (SMD(DMSO)/ B3LYP/6-311++G(3df,2pd)// B3LYP/6-31G(d,p))
	Methyl anion	-39.796028	-39.787379	-39.921582	-39.856638	0.008649	-0.125554	-39.973543
**M1**	REDO_i1_1	-191.918015	-191.882803	-191.924399	-191.988117	0.035212	-0.006383	-191.959289
	REDO_i1_me_1	-231.853632	-231.784191	-231.943587	-231.957660	0.069441	-0.089955	-231.978175
	ΔG_{MA} [kJ/mol]							-119.0
**M2**	REDO_c3_1	-231.242222	-231.181138	-231.247954	-231.322077	0.061084	-0.005733	-231.266726
	REDO_c3_me_1	-271.175466	-271.079880	-271.260892	-271.286296	0.095586	-0.085426	-271.276136
	ΔG_{MA} [kJ/mol]							-94.2
**M3**	REDO_c4_2	-270.558038	-270.470110	-270.564109	-270.648129	0.087928	-0.006072	-270.566273
	REDO_c4_me_1	-310.492882	-310.371014	-310.576525	-310.613557	0.121868	-0.083643	-310.575331
	ΔG_{MA} [kJ/mol]							-93.2
**M4**	REDO_d5_2	-422.980777	-422.871238	-422.992696	-423.116060	0.109539	-0.011919	-423.018440
	REDO_d5_me_1	-462.922634	-462.778142	-463.006667	-463.088570	0.144492	-0.084033	-463.028111
	ΔG_{MA} [kJ/mol]							-94.9
**M5**	REDO_i2_1	-231.242908	-231.181824	-231.250957	-231.323428	0.061084	-0.008048	-231.270392
	REDO_i2_me_1	-271.172711	-271.077290	-271.260402	-271.286556	0.095421	-0.087691	-271.278826
	ΔG_{MA} [kJ/mol]							-91.6
**M6**	REDO_d8_1	-270.564682	-270.478248	-270.571891	-270.654736	0.086434	-0.007209	-270.575511
	REDO_d8_me_1	-310.492232	-310.370375	-310.576055	-310.612538	0.121857	-0.083823	-310.574504
	ΔG_{MA} [kJ/mol]							-66.8
**M7**	REDO_d7_1	-270.562109	-270.475089	-270.570839	-270.652713	0.087020	-0.008730	-270.574423

	REDO_d7_me_1	-310.492110	-310.368810	-310.575659	-310.613938	0.123300	-0.083549	-310.574187
	ΔG_{MA} [kJ/mol]							-68.8
**M8**	REDO_c5_2	-270.559083	-270.471641	-270.567542	-270.650239	0.087442	-0.008459	-270.571256
	REDO_c5_me_1	-310.488659	-310.366958	-310.575798	-310.612606	0.121701	-0.087139	-310.578044
	ΔG_{MA} [kJ/mol]							-87.3
**M9**	REDO_d9_1	-309.880666	-309.767875	-309.888274	-309.981348	0.112791	-0.007608	-309.876165
	REDO_d9_me_2	-349.810641	-349.662218	-349.892601	-349.939981	0.148423	-0.081959	-349.873517
	ΔG_{MA} [kJ/mol]							-62.5
**M10**	REDO_d1_2	-309.876274	-309.762585	-309.884656	-309.977986	0.113689	-0.008382	-309.872678
	REDO_d1_me_3	-349.806352	-349.658561	-349.890765	-349.939297	0.147791	-0.084413	-349.875919
	ΔG_{MA} [kJ/mol]							-78.0
**M11**	REDO_j1_5	-345.769903	-345.679573	-345.782967	-345.895474	0.090330	-0.013064	-345.818208
	REDO_j1_me_1	-385.728671	-385.601316	-385.810742	-385.880908	0.127355	-0.082071	-385.835624
	ΔG_{MA} [kJ/mol]							-115.2
**M12**	REDO_k2_2	-424.399116	-424.257026	-424.409095	-424.542253	0.142090	-0.009980	-424.410143
	REDO_k2_me_6	-464.336063	-464.159079	-464.419347	-464.509774	0.176984	-0.083284	-464.416075
	ΔG_{MA} [kJ/mol]							-85.0
**M13**	REDO_c6_5	-309.875846	-309.762144	-309.884757	-309.977593	0.113702	-0.008912	-309.872803
	REDO_c6_me_1	-349.808250	-349.660388	-349.893620	-349.941483	0.147862	-0.085370	-349.878991
	ΔG_{MA} [kJ/mol]							-85.7
**M14**	REDO_j2_1	-385.086609	-384.969895	-385.099891	-385.222268	0.116714	-0.013282	-385.118836
	REDO_j2_me_5	-425.046221	-424.892369	-425.126745	-425.207475	0.153852	-0.080524	-425.134147
	ΔG_{MA} [kJ/mol]							-109.7
**M15**	REDO_k1_1	-463.721092	-463.551338	-463.733724	-463.875862	0.169754	-0.012631	-463.718740
	REDO_k1_me_4	-503.678261	-503.471030	-503.754772	-503.858591	0.207231	-0.076512	-503.727871
	ΔG_{MA} [kJ/mol]							-93.4

**M16**	REDO_heptenal_sm_6	-349.192414	-349.052486	-349.201785	-349.304745	0.139928	-0.009372	-349.174188
	REDO_heptenal_z_me1	-389.125072	-388.951038	-389.210507	-389.268765	0.174034	-0.085435	-389.180167
	ΔG_{MA} [kJ/mol]							-85.2
**M17**	REDO_j3_2	-424.403103	-424.260133	-424.416793	-424.549299	0.142970	-0.013690	-424.420018
	REDO_j3_me_5	-464.363255	-464.183259	-464.443591	-464.534889	0.179996	-0.080336	-464.435229
	ΔG_{MA} [kJ/mol]							-109.4
**M18**	REDO_c8_6	-388.508941	-388.342793	-388.518748	-388.631841	0.166148	-0.009807	-388.475500
	REDO_c8_me_1	-428.441839	-428.241638	-428.527426	-428.596065	0.200201	-0.085586	-428.481451
	ΔG_{MA} [kJ/mol]							-85.1
**M19**	REDO_j4_1	-463.719708	-463.550470	-463.733819	-463.876493	0.169238	-0.014111	-463.721366
	REDO_j4_me_8	-503.680033	-503.473806	-503.760513	-503.862170	0.206227	-0.080480	-503.736423
	ΔG_{MA} [kJ/mol]							-109.0
**M20**	REDO_i5_5	-465.912235	-465.718285	-465.923361	-466.057924	0.193950	-0.011126	-465.875099
	REDO_i5_me_3	-505.847732	-505.616551	-505.927987	-506.022214	0.231181	-0.080255	-505.871287
	ΔG_{MA} [kJ/mol]							-59.5
**M21**	REDO_c9_6	-427.825467	-427.633000	-427.835713	-427.958932	0.192467	-0.010246	-427.776711
	REDO_c9_me_3	-467.756998	-467.530912	-467.842703	-467.921660	0.226086	-0.085705	-467.781280
	ΔG_{MA} [kJ/mol]							-81.5
**M22**	REDO_j5_1	-503.034905	-502.839469	-503.049570	-503.202112	0.195436	-0.014665	-503.021341
	REDO_j5_me_8	-542.996725	-542.764285	-543.077451	-543.189391	0.232440	-0.080725	-543.037676
	ΔG_{MA} [kJ/mol]							-112.3
**M23**	REDO_c7_9	-426.589054	-426.420075	-426.600138	-426.725468	0.168979	-0.011083	-426.567572
	REDO_c7_me_6	-466.521497	-466.318566	-466.608479	-466.690333	0.202931	-0.086982	-466.574385
	ΔG_{MA} [kJ/mol]							-87.3
**M24**	REDO_d2_3	-467.141984	-466.923270	-467.152666	-467.286009	0.218714	-0.010682	-467.077977
	REDO_d2_me_1	-507.075054	-506.822461	-507.161207	-507.250380	0.252593	-0.086153	-507.083940
	ΔG_{MA} [kJ/mol]							-85.1

**M25**	REDO_j6_2	-542.351407	-542.130080	-542.365984	-542.529800	0.221327	-0.014578	-542.323051
	REDO_j6_me_1	-582.311861	-582.052922	-582.393036	-582.515167	0.258939	-0.081175	-582.337403
	ΔG_{MA} [kJ/mol]							-107.1
**M26**	REDO_j7_2	-581.670815	-581.422442	-581.685464	-581.858547	0.248373	-0.014649	-581.624823
	REDO_j7_me_3	-621.629888	-621.344964	-621.711313	-621.843637	0.284924	-0.081425	-621.640138
	ΔG_{MA} [kJ/mol]							-109.7
**M27**	REDO_j8_2	-620.986526	-620.712325	-621.001121	-621.185797	0.274201	-0.014595	-620.926191
	REDO_j8_me_2	-660.950233	-660.638070	-661.030356	-661.172447	0.312163	-0.080123	-660.940407
	ΔG_{MA} [kJ/mol]							-106.8
**M28**	REDO_c1_10	-387.247611	-387.106584	-387.257439	-387.372770	0.141027	-0.009828	-387.241571
	REDO_c1_me_3	-427.188505	-427.012085	-427.276907	-427.345391	0.176420	-0.088402	-427.257373
	ΔG_{MA} [kJ/mol]							-111.0
**M29**	REDO_c2_2	-421.726202	-421.641391	-421.737083	-421.864057	0.084811	-0.010882	-421.790128
	REDO_c2_me_1_geo	-461.680011	-461.559559	-461.763700	-461.846506	0.120452	-0.083689	-461.809743
	ΔG_{MA} [kJ/mol]							-121.0
**M30**	REDO_a1_1	-270.561057	-270.473673	-270.568330	-270.651156	0.087384	-0.007273	-270.571045
	REDO_a1_me_2	-310.497782	-310.375663	-310.583127	-310.619165	0.122119	-0.085345	-310.582391
	ΔG_{MA} [kJ/mol]							-99.3
**M31**	REDO_a2_1	-309.877494	-309.763885	-309.885157	-309.977992	0.113609	-0.007663	-309.872046
	REDO_a2_me_1	-349.815492	-349.667236	-349.900267	-349.947029	0.148256	-0.084775	-349.883547
	ΔG_{MA} [kJ/mol]							-99.7
**M32**	REDO_a3_1	-349.194018	-349.054189	-349.202152	-349.305099	0.139829	-0.008134	-349.173404
	REDO_a3_me_1	-389.132468	-388.958031	-389.217107	-389.274442	0.174437	-0.084639	-389.184644
	ΔG_{MA} [kJ/mol]							-99.0
**M33**	REDO_k4_1	-270.566698	-270.479635	-270.573222	-270.656490	0.087063	-0.006524	-270.575951
	REDO_k4_me_2	-310.493668	-310.373137	-310.577268	-310.614084	0.120531	-0.083599	-310.577152

	ΔG_{MA} [kJ/mol]							-72.6
**M34**	REDO_a8_1	-309.886761	-309.773701	-309.894743	-309.986154	0.113060	-0.007983	-309.881077
	REDO_a8_me_1	-349.816660	-349.668600	-349.899881	-349.947975	0.148060	-0.083220	-349.883136
	ΔG_{MA} [kJ/mol]							-74.9
**M35**	REDO_a6_1	-388.519660	-388.354156	-388.528523	-388.640032	0.165504	-0.008862	-388.483390
	REDO_a6_me_1	-428.451287	-428.250883	-428.533907	-428.603226	0.200404	-0.082620	-428.485441
	ΔG_{MA} [kJ/mol]							-74.8
**M36**	REDO_b3_1	-309.887652	-309.774991	-309.895490	-309.987539	0.112661	-0.007838	-309.882716
	REDO_b3_me_1	-349.810485	-349.664179	-349.892164	-349.940304	0.146306	-0.081679	-349.875677
	ΔG_{MA} [kJ/mol]							-51.0
**M37**	REDO_a5_2	-270.567929	-270.481160	-270.576801	-270.658667	0.086769	-0.008871	-270.580770
	REDO_a5_me_1	-310.497079	-310.375772	-310.582637	-310.620354	0.121307	-0.085558	-310.584604
	ΔG_{MA} [kJ/mol]							-79.5
**M38**	REDO_a9_9	-349.200855	-349.061497	-349.210606	-349.312785	0.139358	-0.009751	-349.183178
	REDO_a9_me_1	-389.132472	-388.958878	-389.215618	-389.275222	0.173594	-0.083147	-389.184775
	ΔG_{MA} [kJ/mol]							-73.7
**M39**	REDO_b1_10	-388.517412	-388.351807	-388.527629	-388.639923	0.165605	-0.010217	-388.484535
	REDO_b1_me_1	-428.449287	-428.249591	-428.532540	-428.602508	0.199696	-0.083253	-428.486065
	ΔG_{MA} [kJ/mol]							-73.5
**M40**	REDO_b2_10	-427.835075	-427.643448	-427.844884	-427.966892	0.191627	-0.009810	-427.785075
	REDO_b2_me_1	-467.766057	-467.540292	-467.849347	-467.929818	0.225765	-0.083290	-467.787343
	ΔG_{MA} [kJ/mol]							-75.4
**M41**	REDO_b4_1	-309.889482	-309.777238	-309.896846	-309.988608	0.112244	-0.007364	-309.883728
	REDO_b4_me_1	-349.816162	-349.666903	-349.897634	-349.947366	0.149259	-0.081471	-349.879579
	ΔG_{MA} [kJ/mol]							-58.6
**M42**	REDO_s38_1	-229.979031	-229.942369	-229.986544	-230.062459	0.036662	-0.007514	-230.033311

	REDO_s38_me_1	-269.925383	-269.853863	-270.015191	-270.041716	0.071520	-0.089808	-270.060004
	ΔG_{MA} [kJ/mol]							-139.5
**M43**	REDO_a4_1	-269.310353	-269.249924	-269.319851	-269.403699	0.060429	-0.009498	-269.352768
	REDO_a4_me_1	-309.246005	-309.148475	-309.334159	-309.371062	0.097530	-0.088154	-309.361686
	ΔG_{MA} [kJ/mol]							-92.9
**M44**	REDO_s40_1	-461.055363	-460.945369	-461.067571	-461.203712	0.109994	-0.012208	-461.105926
	REDO_s40_me_1_geo	-501.004588	-500.858374	-501.088023	-501.180896	0.146214	-0.083436	-501.118118
	ΔG_{MA} [kJ/mol]							-101.5
**M45**	REDO_s25_1	-344.559792	-344.491526	-344.564092	-344.682385	0.068266	-0.004300	-344.618420
	REDO_s25_me_2	-384.507671	-384.405545	-384.586318	-384.659793	0.102126	-0.078646	-384.636313
	ΔG_{MA} [kJ/mol]							-116.4
**M46**	REDO_e4_1	-382.611097	-382.540714	-382.618557	-382.748691	0.070383	-0.007460	-382.685769
	REDO_e4_me_1	-422.550945	-422.447162	-422.632856	-422.717045	0.103783	-0.081911	-422.695174
	ΔG_{MA} [kJ/mol]							-94.2
**M47**	REDO_e6_3	-383.873145	-383.779175	-383.879726	-384.007423	0.093970	-0.006581	-383.920034
	REDO_e6_me_5	-423.808625	-423.681165	-423.890388	-423.971102	0.127460	-0.081763	-423.925405
	ΔG_{MA} [kJ/mol]							-83.6
**M48**	REDO_e5_5	-421.006849	-420.912312	-421.016937	-421.161743	0.094537	-0.010088	-421.077294
	REDO_e5_me_1	-460.959985	-460.830140	-461.039119	-461.139742	0.129845	-0.079135	-461.089031
	ΔG_{MA} [kJ/mol]							-100.3
**M49**	REDO_e7_9	-460.329314	-460.208734	-460.339281	-460.494273	0.120580	-0.009966	-460.383659
	REDO_e7_me_1	-500.282433	-500.126524	-500.361094	-500.472012	0.155909	-0.078661	-500.394764
	ΔG_{MA} [kJ/mol]							-98.6
**M50**	REDO_f1_4	-385.114737	-384.997384	-385.121528	-385.245495	0.117353	-0.006791	-385.134933
	REDO_f1_me_3	-425.046330	-424.895343	-425.129172	-425.205218	0.150987	-0.082842	-425.137073
	ΔG_{MA} [kJ/mol]							-75.1

**M51**	REDO_f2_1	-385.119670	-385.001940	-385.125603	-385.249716	0.117730	-0.005933	-385.137919
	REDO_f2me_1	-425.050950	-424.900204	-425.133080	-425.209506	0.150746	-0.082129	-425.140889
	ΔG_{MA} [kJ/mol]							-77.3
**M52**	REDO_f3_5	-424.431136	-424.287561	-424.438385	-424.572486	0.143575	-0.007249	-424.436160
	REDO_f3_me_3	-464.363013	-464.185753	-464.445904	-464.532388	0.177260	-0.082891	-464.438019
	ΔG_{MA} [kJ/mol]							-74.3
**M53**	REDO_s64_10	-503.064217	-502.868172	-503.072358	-503.226701	0.196045	-0.008140	-503.038796
	REDO_s64_me_3	-542.996417	-542.766784	-543.079781	-543.186837	0.229633	-0.083364	-543.040568
	ΔG_{MA} [kJ/mol]							-74.1
**M54**	REDO_g2_1	-421.932781	-421.836619	-421.939734	-422.080027	0.096162	-0.006953	-421.990818
	REDO_g2_me_2	-461.868901	-461.739398	-461.946956	-462.042630	0.129503	-0.078055	-461.991182
	ΔG_{MA} [kJ/mol]							-70.4
**M55**	REDO_s62_2	-423.194194	-423.075700	-423.200225	-423.337971	0.118494	-0.006031	-423.225508
	REDO_s62_me_2	-463.126791	-462.973723	-463.203977	-463.297092	0.153068	-0.077185	-463.221209
	ΔG_{MA} [kJ/mol]							-58.2
**M56**	REDO_s37_6	-460.328261	-460.207879	-460.338018	-460.492747	0.120382	-0.009757	-460.382123
	REDO_s37_me_1	-500.278211	-500.122503	-500.353902	-500.465704	0.155708	-0.075690	-500.385687
	ΔG_{MA} [kJ/mol]							-78.8
**M57**	REDO_s51_5	-499.650642	-499.503808	-499.659726	-499.824702	0.146834	-0.009084	-499.686953
	REDO_s51_me_1	-539.600643	-539.418866	-539.675892	-539.797965	0.181777	-0.075249	-539.691438
	ΔG_{MA} [kJ/mol]							-81.2
**M58**	REDO_g5_1	-345.798024	-345.707217	-345.802809	-345.917744	0.090807	-0.004785	-345.831722
	REDO_g5_me_2	-385.723908	-385.601028	-385.802900	-385.870870	0.122880	-0.078992	-385.826982
	ΔG_{MA} [kJ/mol]							-57.0
**M59**	REDO_s61_10	-463.753656	-463.584181	-463.759741	-463.904147	0.169475	-0.006085	-463.740757
	REDO_s61_me_8	-503.683880	-503.480710	-503.760830	-503.859937	0.203170	-0.076950	-503.733717
	ΔG_{MA} [kJ/mol]							-51.0

**M60**	REDO_s35_1	-383.884854	-383.790704	-383.890649	-384.017526	0.094150	-0.005794	-383.929170
	REDO_s35_me_1	-423.826011	-423.697914	-423.903119	-423.988231	0.128097	-0.077108	-423.937242
	ΔG_{MA} [kJ/mol]							-90.7
**M61**	REDO_g3_1	-345.801220	-345.710301	-345.807848	-345.921379	0.090919	-0.006628	-345.837089
	REDO_g3_me_1	-385.724244	-385.601022	-385.805589	-385.873751	0.123222	-0.081346	-385.831874
	ΔG_{MA} [kJ/mol]							-55.8
**M62**	REDO_f8_5	-424.434122	-424.290559	-424.441684	-424.575444	0.143563	-0.007562	-424.439443
	REDO_f8_me_1	-464.359591	-464.183742	-464.438973	-464.528681	0.175849	-0.079382	-464.432214
	ΔG_{MA} [kJ/mol]							-50.5
**M63**	REDO_f9_6	-503.067191	-502.871141	-503.075660	-503.229650	0.196050	-0.008468	-503.042068
	REDO_f9_me_1	-542.993147	-542.764966	-543.072783	-543.183266	0.228181	-0.079636	-543.034721
	ΔG_{MA} [kJ/mol]							-50.2
**M64**	REDO_g7_2	-385.119375	-385.003005	-385.125372	-385.248982	0.116370	-0.005997	-385.138609
	REDO_g7_me_1	-425.040635	-424.894278	-425.117847	-425.197066	0.146357	-0.077212	-425.127922
	ΔG_{MA} [kJ/mol]							-41.4
**M65**	REDO_e1_1	-305.206873	-305.165722	-305.213310	-305.320452	0.041151	-0.006436	-305.285737
	REDO_e1_me_1	-345.152337	-345.077868	-345.241631	-345.296744	0.074469	-0.089294	-345.311570
	ΔG_{MA} [kJ/mol]							-137.3
**M66**	REDO_e2_2	-344.528890	-344.461676	-344.536280	-344.652701	0.067214	-0.007390	-344.592877
	REDO_e2_me_1	-384.474398	-384.374038	-384.563758	-384.628411	0.100360	-0.089360	-384.617411
	ΔG_{MA} [kJ/mol]							-133.9
**M67**	REDO_e3_1	-423.166769	-423.046397	-423.173448	-423.309934	0.120372	-0.006679	-423.196241
	REDO_e3_me_3	-463.113635	-462.959444	-463.198988	-463.284991	0.154191	-0.085353	-463.216153
	ΔG_{MA} [kJ/mol]							-121.7
**M68**	REDO_f7_1	-344.538437	-344.474223	-344.547013	-344.661982	0.064214	-0.008577	-344.606345
	REDO_f7_me_1	-384.472952	-384.372716	-384.560795	-384.626113	0.100236	-0.087844	-384.613721

	ΔG_{MA} [kJ/mol]							-88.8
**M69**	REDO_f4_7	-423.171856	-423.053801	-423.181180	-423.316533	0.118055	-0.009325	-423.207803
	REDO_f4_me_2_geo	-463.106414	-462.954081	-463.193210	-463.280169	0.152333	-0.086796	-463.214631
	ΔG_{MA} [kJ/mol]							-87.4
**M70**	REDO_s44_3	-462.493614	-462.348919	-462.503764	-462.648212	0.144695	-0.010150	-462.513667
	REDO_s44_me_2_geo2	-502.428423	-502.250172	-502.515311	-502.611814	0.178251	-0.086888	-502.520451
	ΔG_{MA} [kJ/mol]							-87.3
**M71**	REDO_f5_8	-501.804833	-501.633796	-501.814833	-501.970453	0.171037	-0.010000	-501.809416
	REDO_f5_me_8	-541.739889	-541.535104	-541.827089	-541.934658	0.204785	-0.087199	-541.817073
	ΔG_{MA} [kJ/mol]							-89.6
**M72**	REDO_g1_1	-247.303114	-247.252383	-247.314463	-247.395434	0.050731	-0.011349	-247.356052
	REDO_g1_me_1	-287.222902	-287.137727	-287.314249	-287.348744	0.085175	-0.091347	-287.354916
	ΔG_{MA} [kJ/mol]							-66.5
**M73**	REDO_s29_1	-325.698761	-325.607659	-325.705499	-325.798965	0.091102	-0.006738	-325.714601
	REDO_s29_me_1	-365.620293	-365.496916	-365.706127	-365.749589	0.123377	-0.085834	-365.712046
	ΔG_{MA} [kJ/mol]							-62.8

Applications

Example 1: Prediction of rate constants for the reactions of Michael acceptors with nucleophiles

The GSH-derived Mayr electrophilicity parameters E (Table S4) can be applied in equation 1 to predict second-order rate constants for the reactions of the Michael acceptors **M1**–**M73** with further types of nucleophiles, whose nucleophilicity parameters N and s_N have been determined.

While N and s_N for nucleophiles are solvent-dependent, the same electrophilicity parameter E for a certain Michael acceptor can be used in different solvents. Within a total reactivity range of 40 orders of magnitude, using equation 1 usually allows to calculate second-order rate constants within a precision of factor <100 for reactions at 20 °C, in which exactly one new C-X or C-C σ-bond is formed.[S19] Significant deviations of more than a factor of 1000 may indicate the operation of a different mechanism, the influence of specific solvation on the energetics of the observed reaction or further effects that alter the energy profile of a reaction.

Table S6 compares the second-order rate constants $k_2^{\text{eq 1}}$ (M^{-1} s^{-1}) calculated by equation 1 for reactions at 20 °C with reported experimental second-order rate constants k_2^{exp} (M^{-1} s^{-1}) for the reactions of Michael acceptors with diverse nitrogen-, oxygen-, or sulfur-centered nucleophiles in different solvents at the given temperature.

For example, a second-order rate constant k_2^{exp} = 4.3 M^{-1} s^{-1} has been experimentally determined by Heo and Bunting for the reaction of acrolein (**M1**) with the pyridone anion in aqueous solution at 25 °C (Table S6, entry 1).[S20] Using equation 1, the

electrophilicity E = –14.4 of acrolein (**M1**) from Table S4, and the

nucleophile-specific reactivity parameters of the 4-pyridone anion (in H_2O):

N = 14.76, s_N = 0.48 (from Mayr's reactivity database[S21]) we calculate

$\lg k_2^{\text{eq 1}} = s_N(N + E) = 0.17$, which corresponds to $k_2^{\text{eq 1}}$ = 1.5 M^{-1} s^{-1} (at 20 °C).

The comparison with second-order rate constants $k_2^{\text{eq 1}}$ (20 °C) calculated by using equation 1 and the Mayr reactivity parameters E, N, and s_N shows that $k_2^{\text{exp}}/k_2^{\text{eq 1}} < 100$ is fulfilled for 71 Michael additions (Table S6). In the reaction of hydrazine (in water) with the butynone **M42** (Table S6, entry 28) a lowering of the activation barrier may be due to the simultaneous C–N and C–H bond formation in the transition state of, as proposed by Um and coworkers.[S28] Only for methyl crotonate (**M61**) the ratio

$k_2^{exp}/k_2^{eq\ 1}$ is in the range of 130 to 600 for 5 of 6 available k_2^{exp} at 30 °C (Table S6, entries 48–53). The slight exceeding of the precision criterion of $k_2^{exp}/k_2^{eq\ 1} < 100$ may in part be due to the 10 K difference in the reference temperatures for k_2^{exp} and $k_2^{eq\ 1}$ but we refrain from a further interpretation of this deviation. The analysis in Table S6 proves that the GSH-derived *E* for Michael acceptors can, in general, be used to predict reaction rates for Michael additions of other classes of nucleophiles.

Table S6: Experimental (k_2^{exp}) and calculated second-order rate constants ($k_2^{eq\,1}$) for the 1,4-additions of various N-, O- and S-centered nucleophiles to Michael acceptors **M1**, **M2**, **M5**, **E11**, **M30**, **M33**, **M42**, **E12**, **M58**, **M61**, **M66**, **M72**, and **E15**.

Entry	Electrophile	E[a]	Nucleophile (Solvent)	N (s_N)[b]	k_2^{exp} (M^{-1} s^{-1})	T (°C)	Reference	$k_2^{eq\,1}$ (M^{-1} s^{-1})	$k_2^{exp}/k_2^{eq\,1}$
1	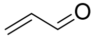 **M1**	−14.4	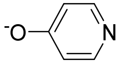 (in H_2O)	14.76 (0.48)	**4.3**	25	[S20]	**1.49**	2.9
2	**M1**	−14.4	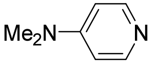 (in H_2O)	13.19 (0.56)	**2.39**	25	[S20]	**0.21**	11
3	**M1**	−14.4	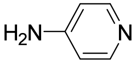 (in H_2O)	12.19 (0.66)	**1.65**	25	[S20]	**3.48 × 10^{-2}**	47
4	**M1**	−14.4	morpholine (in MeOH)	15.40 (0.64)	**1.28**	30	[S22]	**4.37**	0.29
5	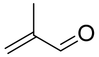 **M2**	−17.2	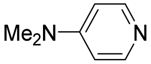 (in H_2O)	13.19 (0.56)	**1.8 × 10^{-2}**	25	[S20]	**5.68 × 10^{-3}**	3.2
6	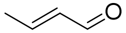 **M5**	−18.4	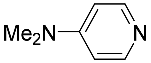 (in H_2O)	13.19 (0.56)	**8 × 10^{-3}**	25	[S20]	**1.21 × 10^{-3}**	6.6
7	**M5**	−18.4	$^{-}O_2C$-CH_2-S^{-} (in H_2O)	22.62 (0.43)	**15**	25	[S23]	**65.3**	0.23
8	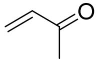 **E11**	−16.8	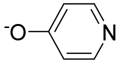 (in H_2O)	14.76 (0.48)	**0.96**	25	[S20]	**0.105**	9.2
9	**E11**	−16.8	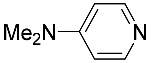 (in H_2O)	13.19 (0.56)	**0.55**	25	[S20]	**9.51 × 10^{-3}**	58

10	**E11**	−16.8	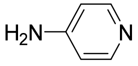 (in H_2O)	12.19 (0.66)	**0.42**	25	[S20]	**9.07 × 10^{-4}**	4.6 × 10^{2}
11	**E11**	−16.8	morpholine (in MeOH)	15.40 (0.64)	**1.20**	30	[S22]	**0.127**	9.4
12	**E11**	−16.8	Gly^{-} (in H_2O)	13.51 (0.58)	**0.400**	30	[S24]	**1.24 × 10^{-2}**	32
13	**E11**	−16.8	Ala^{-} (in H_2O)	13.01 (0.58)	**0.228**	30	[S24]	**6.34 × 10^{-3}**	36
14	**E11**	−16.8	MeO^{-} (in MeOH)	15.78 (0.56)	**0.378**	19	[S25]	**0.268**	1.4
15	**E11**	−16.8	EtO^{-} (in EtOH)	15.78 (0.65)	**1.56**	19	[S25]	**0.217**	7.2
16	**E11**	−16.8	$iPrO^{-}$ (in iPrOH)	17.03 (0.63)	**3.15**	19	[S25]	**1.40**	2.3
17	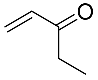 **M30**	−15.8	MeO^{-} (in MeOH)	15.78 (0.56)	**0.235**	24	[S26]	**0.975**	0.24
18	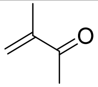 **M33**	−18.6	MeO^{-} (in MeOH)	15.78 (0.56)	**8.0 × 10^{-3}** [c]	24	[S26]	**2.64 × 10^{-2}**	0.30
19	**M33**	−18.6	$iPrO^{-}$ (in iPrOH)	17.03 (0.63)	**2.6 × 10^{-2}** [c]	24	[S26]	**0.103**	0.25
20	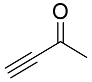 **M42**	−16.6	aniline (in H_2O)	12.99 (0.73)	**0.375**	25	[S27]	**2.32 × 10^{-3}**	1.6 × 10^{2}
21	**M42**	−16.6	*p*-anisidine (in H_2O)	14.28 (0.68)	**1.34**	25	[S27]	**2.64 × 10^{-2}**	51
22	**M42**	−16.6	$EtGly^{-}$ (in H_2O)	12.08 (0.60)	**0.147**	25	[S28]	**1.94 × 10^{-3}**	76
23	**M42**	−16.6	ethanolamine (in H_2O)	12.61 (0.58)	**0.336**	25	[S28]	**4.85 × 10^{-3}**	69
24	**M42**	−16.6	$GlyGly^{-}$ (in H_2O)	12.91 (0.59)	**0.160**	25	[S28]	**6.65 × 10^{-3}**	24

25	**M42**	−16.6	Gly$^-$ (in H_2O)	13.51 (0.58)	**0.594**	25	[S28]	**1.61 × 10^{-2}**	37
26	**M42**	−16.6	ethylamine (in H_2O)	12.87 (0.58)	**0.670**	25	[S28]	**6.86 × 10^{-3}**	98
27	**M42**	−16.6	benzylamine (in H_2O)	13.44 (0.55)	**0.454**	25	[S28]	**1.83 × 10^{-2}**	25
28	**M42**	−16.6	hydrazine (in H_2O)	13.46 (0.57)	**3.66**	25	[S28]	**1.62 × 10^{-2}**	2.3 × 10^{2} [d]
29	**M42**	−16.6	piperidine (in H_2O)	18.13 (0.44)	**4.12**	25	[S29]	**4.71**	0.87
30	**M42**	−16.6	morpholine (in H_2O)	15.62 (0.54)	**10.9**	25	[S29]	**0.296**	37
31	**M42**	−16.6	piperazine (in H_2O)	17.22 (0.50)	**13.3**	25	[S29]	**2.04**	6.5
32	**M42**	−16.6	piperidine (in MeCN)	17.35 (0.68)	**5.62**	25	[S29]	**3.24**	1.7
33	**M42**	−16.6	morpholine (in MeCN)	15.65 (0.74)	**0.520**	25	[S29]	**0.198**	2.6
34	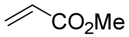 **E12**	−18.8	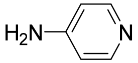 (in H_2O)	12.19 (0.66)	**1.46 × 10^{-2}**	25	[S20]	**7.22 × 10^{-4}**	20
35	**E12**	−18.8	morpholine (in MeOH)	15.40 (0.64)	**1.04 × 10^{-2}**	30	[S22]	**6.67 × 10^{-3}**	1.6
36	**E12**	−18.8	GlyGly$^-$ (in H_2O)	12.91 (0.59)	**4.6 × 10^{-3}**	30	[S24]	**3.35 × 10^{-4}**	14
37	**E12**	−18.8	Gly$^-$ (in H_2O)	13.51 (0.58)	**1.82 × 10^{-2}**	30	[S24]	**8.55 × 10^{-4}**	21
38	**E12**	−18.8	β-Ala$^-$ (in H_2O)	13.26 (0.58)	**2.84 × 10^{-2}**	30	[S24]	**6.12 × 10^{-4}**	46
39	**E12**	−18.8	Phe$^-$ (in H_2O)	14.12 (0.53)	**6.40 × 10^{-3}**	30	[S24]	**3.31 × 10^{-3}**	1.9
40	**E12**	−18.8	Met$^-$ (in H_2O	13.16 (0.58)	**6.90 × 10^{-3}**	30	[S24]	**5.36 × 10^{-4}**	13
41	**E12**	−18.8	Ala$^-$ (in H_2O)	13.01 (0.58)	**1.11 × 10^{-2}**	30	[S24]	**4.38 × 10^{-4}**	25
42	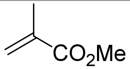 **M58**	−23.4	GlyGly$^-$ (in H_2O)	12.91 (0.59)	**1.35 × 10^{-5}**	30	[S24]	**6.47 × 10^{-7}**	21

43	**M58**	−23.4	Gly⁻ (in H_2O)	13.51 (0.58)	**5.80 × 10^{-5}**	30	[S24]	**1.84 × 10^{-6}**	32
44	**M58**	−23.4	β-Ala⁻ (in H_2O	13.26 (0.58)	**1.37 × 10^{-4}**	30	[S24]	**1.31 × 10^{-6}**	1.0 × 10^{2}
45	**M58**	−23.4	Phe⁻ (in H_2O)	14.12 (0.53)	**2.48 × 10^{-5}**	30	[S24]	**1.21 × 10^{-5}**	2.1
46	**M58**	−23.4	Met⁻ (in H_2O	13.16 (0.58)	**2.93 × 10^{-5}**	30	[S24]	**1.15 × 10^{-6}**	26
47	**M58**	−23.4	Ala⁻ (in H_2O)	13.01 (0.58)	**3.64 × 10^{-5}**	30	[S24]	**9.41 × 10^{-7}**	39
48	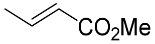 **M61**	−22.8	GlyGly⁻ (in H_2O)	12.91 (0.59)	**2.20 × 10^{-4}**	30	[S24]	**1.46 × 10^{-6}**	1.5 × 10^{2}
49	**M61**	−22.8	Gly⁻ (in H_2O)	13.51 (0.58)	**1.20 × 10^{-3}**	30	[S24]	**4.09 × 10^{-6}**	2.9 × 10^{2}
50	**M61**	−22.8	β-Ala⁻ (in H_2O)	13.26 (0.58)	**1.76 × 10^{-3}**	30	[S24]	**2.93 × 10^{-6}**	6.0 × 10^{2}
51	**M61**	−22.8	Phe⁻ (in H_2O)	14.12 (0.53)	**3.31 × 10^{-4}**	30	[S24]	**2.51 × 10^{-5}**	13
52	**M61**	−22.8	Met⁻ (in H_2O	13.16 (0.58)	**3.36 × 10^{-4}**	30	[S24]	**2.56 × 10^{-6}**	1.3 × 10^{2}
53	**M61**	−22.8	Ala⁻ (in H_2O)	13.01 (0.58)	**5.85 × 10^{-4}**	30	[S24]	**2.10 × 10^{-6}**	2.8 × 10^{2}
54	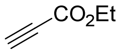 **M66**	−17.7	piperidine (in H_2O)	18.13 (0.44)	**1.24**	25	[S30]	**1.55**	0.80
55	**M66**	−17.7	morpholine (in H_2O)	15.62 (0.54)	**0.245**	25	[S30]	**7.53× 10^{-2}**	3.3
56	**M66**	−17.7	piperazine (in H_2O)	17.22 (0.50)	**0.935**	25	[S30]	**0.575**	1.6
57	**M66**	−17.7	piperidine (in MeCN)	17.35 (0.68)	**0.902**	25	[S30]	**0.578**	1.6
58	**M66**	−17.7	morpholine (in MeCN)	15.65 (0.74)	**7.48 × 10^{-2}**	25	[S30]	**3.04 × 10^{-2}**	2.5
59	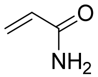 **M72**	−21.8	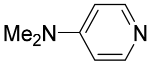 (in H_2O)	13.19 (0.56)	**6.1 × 10^{-4}**	25	[S20]	**1.51 × 10^{-5}**	41

60	**M72**	−21.8	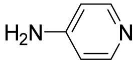 (in H_2O)	12.19 (0.66)	**5.3 × 10^{-4}**	25	[S20]	**4.54 × 10^{-7}**	1.2 × 10^{3}
61	**M72**	−21.8	morpholine (in MeOH)	15.40 (0.64)	**3.57 × 10^{-4}**	30	[S22]	**8.02 × 10^{-5}**	0.65
62	**M72**	−21.8	pyrrolidine (in MeOH)	15.97 (0.63)	**8.27× 10^{-3}**	30	[S22]	**2.12 × 10^{-4}**	1.6
63	**M72**	−21.8	GlyGly^{-} (in H_2O)	12.91 (0.59)	**2.0 × 10^{-4}**	30	[S24]	**5.69 × 10^{-6}**	35
64	**M72**	−21.8	Gly^{-} (in H_2O)	13.51 (0.58)	**6.3 × 10^{-4}**	30	[S24]	**1.56 × 10^{-5}**	41
65	**M72**	−21.8	Phe^{-} (in H_2O)	14.12 (0.53)	**2.2 × 10^{-4}**	30	[S24]	**8.50 × 10^{-5}**	2.6
66	**M72**	−21.8	Ala^{-} (in H_2O)	13.01 (0.58)	**3.5 × 10^{-4}**	30	[S24]	**7.98 × 10^{-6}**	44
67	**M72**	−21.8	iPrO^{-} (in iPrOH)	17.03 (0.63)	**3.2 × 10^{-4}**	24	[S26]	**9.88 × 10^{-4}**	0.30
68	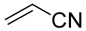 **E15**	−19.1	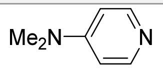 (in H_2O)	13.19 (0.56)	**4.03 × 10^{-3}**	25	[S20]	**4.90 × 10^{-4}**	8.2
69	**E15**	−19.1	morpholine (in MeOH)	15.40 (0.64)	**5.68 × 10^{-3}**	30	[S22]	**4.29 × 10^{-3}**	1.3
70	**E15**	−19.1	GlyGly^{-} (in H_2O)	12.91 (0.59)	**1.37 × 10^{-3}**	30	[S24]	**2.23 × 10^{-4}**	6.2
71	**E15**	−19.1	Gly^{-} (in H_2O)	13.51 (0.58)	**5.00 × 10^{-3}**	30	[S24]	**5.73 × 10^{-4}**	8.7
72	**E15**	−19.1	β-Ala^{-} (in H_2O)	13.26 (0.58)	**8.92 × 10^{-3}**	30	[S24]	**4.10 × 10^{-4}**	22
73	**E15**	−19.1	Phe^{-} (in H_2O)	14.12 (0.53)	**1.76 × 10^{-3}**	30	[S24]	**2.29 × 10^{-3}**	0.77
74	**E15**	−19.1	Met^{-} (in H_2O	13.16 (0.58)	**1.76 × 10^{-3}**	30	[S24]	**3.59 × 10^{-4}**	4.9
75	**E15**	−19.1	Ala^{-} (in H_2O)	13.01 (0.58)	**3.53 × 10^{-3}**	30	[S24]	**2.94 × 10^{-4}**	12
76	**E15**	−19.1	MeO^{-} (in MeOH)	15.78 (0.56)	**5.80 × 10^{-3}**	20	[S31]	**1.38 × 10^{-2}**	0.42
77	**E15**	−19.1	MeO^{-} (in MeOH)	15.78 (0.56)	**1.22 × 10^{-2}**	24	[S26]	**1.38 × 10^{-2}**	0.88
78	**E15**	−19.1	EtO^{-} (in EtOH)	15.78 (0.65)	**5.25 × 10^{-2}**	20	[S31]	**6.95 × 10^{-3}**	7.6

79	**E15**	−19.1	$nPrO^-$ (in nPrOH)	16.03 (0.70)	**9.58×10^{-2}**	20	[S31]	**7.10×10^{-3}**	14
80	**E15**	−19.1	$iPrO^-$ (in iPrOH)	17.03 (0.63)	**0.287**	20	[S31]	**4.96×10^{-2}**	5.8

[a] From Table S4. [b] From ref[S21]. [c] Second-order rate constants during the initial phase of the reactions. [d] Concerted C–N and C–H bond formation in the transition state was proposed in ref[S28].

Example 2: Estimating the electrophilicity of fluorescent thiol probes

Rate constants with GSH are an often used measure for the reactivity of fluorescent electrophilic thiol probes. Electrophilicity parameters *E* for these fluorescence probes can be derived from the available rate constants k_{GSH}. Once the electrophilicity parameter *E* of a fluorescent thiol probe is known, rate constants of their reactions with further competing nucleophilic sites in peptides can be estimated by using equation 1.

For example, Jiang et al. introduced the GSH probe 'RealThiol' for quantitative real-time imaging of glutathione in living cells. For the reaction of RealThiol with GSH the authors measured a second-order rate constant k_{GSH} = 7.5 M^{-1} s^{-1} (pH 7.4, 37 °C).[S32] Applying k_{GSH} in equation 2 (with *F* = 0.0280) gives a second-order rate constant k_2 = 2.7 × 10^2 M^{-1} s^{-1}. With equation 1 and the nucleophilicity parameter of GSH(NH_3^+/S^-), that is *N* = 20.97 (s_N = 0.56), an approximate electrophilicity of *E* = –16.6 can be calculated for RealThiol (Table S7, entry 1). Further examples for estimated electrophilicity parameters *E* for fluorescent thiol probes are collected in Table S7.

Though in lack of the nucleophilicity parameters for GSH, Kamyia and Urano used Mayr electrophilicity parameters *E* for a rational design strategy to develop highly reactive fluorescence probes.[S33] ThiolQuant Green (TQ Green)[S34] was reported to undergo relatively slow reactions with GSH (see Table S7, entry 2). Kamiya, Urano and coworkers then estimated the electrophilicity *E* of TQ Green based on a comparison with the structurally analogous chalcone fragment whose *E* is available in Mayr's database (*E* = –19).[S21] They concluded, that in order to achieve the required higher reactivity of the GSH probe for real-time dynamic quantification of GSH in living cells, a carbocationic fragment in the GSH probe would be more suitable. The nucleophilicity of GSH (*N* = 20.97, s_N = 0.56), determined in this work, can now be used to estimate the electrophilicities *E* of the fluorescent probes developed by Urano and Kamiya (Table S7, entries 7–9), which may help to further improve the performance of these fluorescent probes in real-time dynamic GSH monitoring in living cells.

Table S7: Rate constants k_{GSH}^{exp} (at pH 7.4) for reactions of fluorescence probes **M74**–**M82** with GSH, estimated electrophilicities E of the probes, and their quantum-chemically calculated methyl anion affinities ΔG_{MA}.

Entry	Probe	k_{GSH}^{exp} (M^{-1} s^{-1})	T	Reference	$k_2(GS^-)$ (M^{-1} s^{-1})	Estimated E[a]	ΔG_{MA}[b] (kJ mol^{-1})
1	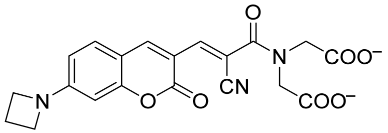 **M74** (RealThiol)	7.5	37 °C	[S32]	2.7×10^2	–16.6	–108.4
2	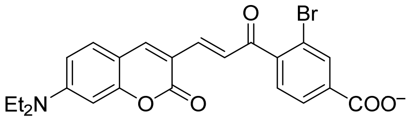 **M75** (TQ Green)	0.15	– [c]	[S34]	5.4	–19.7	–70.9
3	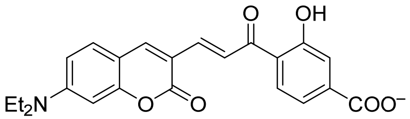 **M76**	1.29	– [c]	[S35]	46.1	–18.0	–85.2
4	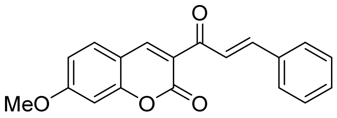 **M77**	--	30 °C	[S36]	1.56[d]	–20.6	–83.9
5	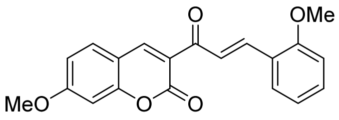 **M78**	--	30 °C	[S36]	44.4[d]	–18.0	–79.3

6	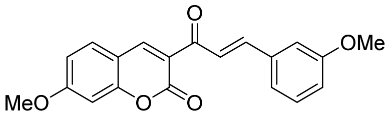 **M79**	--	30 °C	[S36]	19.9[d]	−18.6	−81.1
7	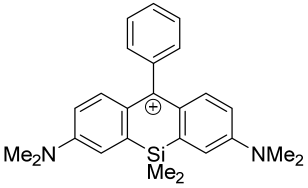 **M80** (Ph SiR650)	560	– [c]	[S33]	2.00×10^4	−13.2	−148.4
8	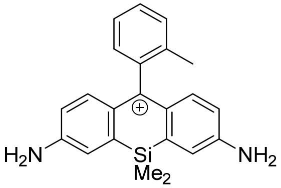 **M81** (2’Me SiR600)	272	– [c]	[S33]	9.71×10^3	−13.8	−151.2
9	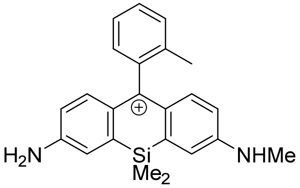 **M82** (2’Me-SiR610)	153	– [c]	[S33]	5.46×10^3	−14.2	−144.4

[a] Calculated by using equation 1 (main text), lg k_2(GS^-), and the reactivity parameters for GS^- (N = 20.97, s_N = 0.55$_5$). [b] For details of the quantum-chemical calculation of methyl anion affinities ΔG_{MA} see Table S8. [c] Temperature not reported. [d] As reported in ref.[S36]

Quantum-chemically calculated methyl anion affinities (ΔG_{MA}) of fluorescent thiol probes

The methyl anion affinities ΔG_{MA} for **M74**–**M82** listed in column 8 of Table S7 were calculated by following the same procedure as described on page S32. Figure S3 shows the linear correlation of electrophilicities E with ΔG_{MA} for 117 Michael acceptors (black dots: from ref[S10c], orange dots: this work). Purple rhombs (◆) show the additional E vs ΔG_{MA} data for the 9 fluorescence probes **M74**–**M82** from Table S7.

Figure S3 demonstrates that the correlation between quantum chemically calculated methyl anion affinities ΔG_{MA} and the electrophilicities E of prototypical Michael acceptors is also applicable for roughly estimating the reactivity of the structurally more sophisticated fluorescence probes **M74**–**M82**.

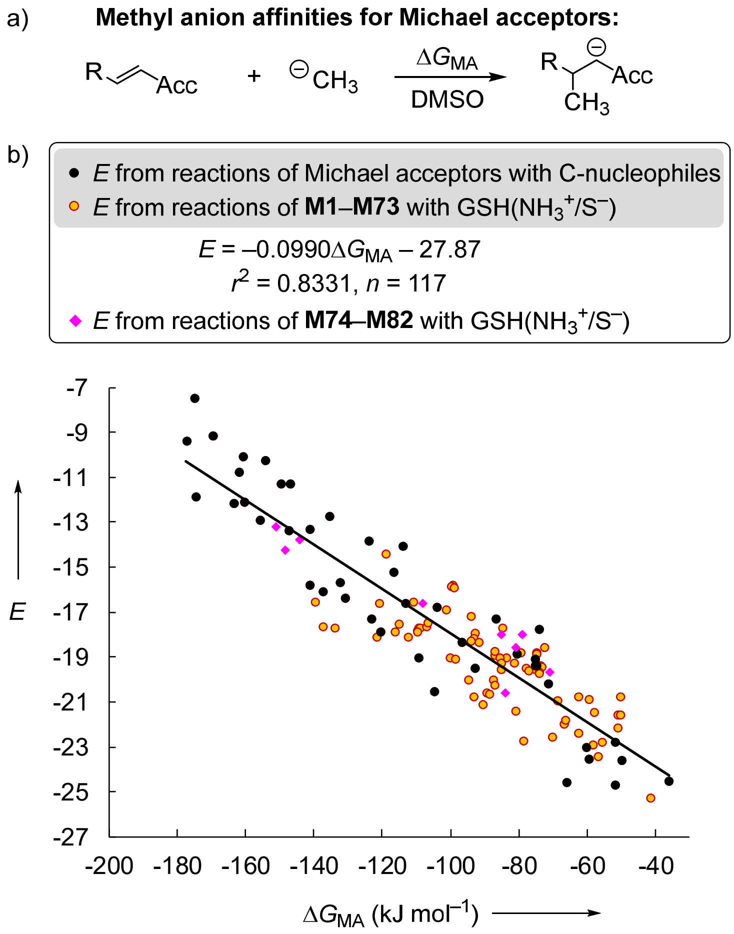

Figure S3. Correlation of experimentally determined Michael acceptor electrophilicities E and methyl anion affinities ΔG_{MA} in DMSO [calculated at the SMD(DMSO)/B3LYP/6-311++G(3df,2pd)//B3LYP/6-31G(d,p) level of theory, ref[S10c] and this work] supplemented by data for the fluorescent thiol probes **M74**–**M82** (purple rhombs ◆, with data from Table S7, not included when calculating the correlation line; details of the quantum-chemical calculations for **M74**–**M82** are given in Table S8).

Table S8: Quantum-mechanically calculated methyl anion affinities ΔG_{MA} for Michael acceptors **M74**–**M82** [in kJ mol^{-1}, calculated at the SMD(DMSO)/B3LYP/6-311++G(3df,2pd)//B3LYP/6-31G(d,p) level of theory]

Michael acceptor	Filename	E_{tot} (B3LYP/6-31G(d,p)	G_{298}	$E_{tot, solv.}$ (SMD(DMSO)/B3LYP/6-31G(d,p)// B3LYP/6-31G(d,p))	$E_{tot, high.}$ (B3LYP/6-311++G(3df,2pd)// B3LYP/6-31G(d,p))	ΔG	ΔG_{solv}	G_{298} (SMD(DMSO)/ B3LYP/6-311++G(3df,2pd)// B3LYP/6-31G(d,p))
	Methyl anion	-39.796028	-39.787379	-39.921582	-39.856638	0.008649	-0.125554	-39.973543
**M74**	REDO_rt_sm_4	-1461.991496	-1461.726753	-1462.242350	-1462.528398	0.264743	-0.250854	-1462.514509
	REDO_rt_me_e_10	-1501.767394	-1501.464592	-1502.265339	-1502.334190	0.302802	-0.497945	-1502.529333
	ΔG_{MA} [kJ/mol]							-108.4
**M75**	REDO_tq_green_3	-3890.559432	-3890.249532	-3890.661877	-3893.442293	0.309900	-0.102446	-3893.234839
	REDO_tq_green_me_z_6	-3930.424837	-3930.081397	-3930.666410	-3933.337252	0.343440	-0.241572	-3933.235385
	ΔGMA [kJ/mol]							-70.9
**M76**	REDO_tq_oh_4	-1394.693066	-1394.368129	-1394.798509	-1395.165252	0.324937	-0.105443	-1394.945758
	REDO_tq_oh_me_z_1	-1434.562243	-1434.201384	-1434.808150	-1435.066722	0.360859	-0.245907	-1434.951770
	ΔG_{MA} [kJ/mol]							-85.2
**M77**	REDO_og1_1	-1033.361672	-1033.127333	-1033.383033	-1033.690881	0.234339	-0.021361	-1033.477902
	REDO_og1_me_6	-1073.309088	-1073.041347	-1073.392650	-1073.667583	0.267741	-0.083562	-1073.483404
	ΔG_{MA} [kJ/mol]							-83.9
**M78**	REDO_og5_2	-1147.885858	-1147.622673	-1147.908687	-1148.255234	0.263185	-0.022829	-1148.014878
	REDO_og5_me_6	-1187.830709	-1187.532938	-1187.917590	-1188.229515	0.297771	-0.086881	-1188.018625
	ΔGMA [kJ/mol]							-79.3
**M79**	REDO_og3_1	-1147.887399	-1147.623893	-1147.909568	-1148.257465	0.263506	-0.022169	-1148.016127
	REDO_og3_me_4	-1187.836132	-1187.539153	-1187.918804	-1188.234850	0.296979	-0.082673	-1188.020543
	ΔG_{MA} [kJ/mol]							-81.1

**M80**	REDO_sir650_1	-1369.005284	-1368.586678	-1369.069411	-1369.337823	0.418606	-0.064127	-1368.983344
	REDO_sir650_me_2	-1409.102782	-1408.645544	-1409.118965	-1409.454462	0.457238	-0.016183	-1409.013407
	ΔGMA [kJ/mol]							-148.4
**M81**	REDO_sir600_1	-1251.087586	-1250.746939	-1251.160216	-1251.392682	0.340647	-0.072630	-1251.124664
	REDO_sir600_me_1	-1291.189851	-1290.810080	-1291.209840	-1291.515573	0.379771	-0.019988	-1291.155791
	ΔGMA [kJ/mol]							-151.2
**M82**	REDO_sir610_2	-1290.399281	-1290.032363	-1290.469631	-1290.713713	0.366918	-0.070350	-1290.417145
	REDO_sir610_me_2	-1330.498349	-1330.092475	-1330.517105	-1330.832799	0.405874	-0.018757	-1330.445682
	ΔGMA [kJ/mol]							-144.4

Example 3: Quantitative structure/reactivity relationships for α,β-unsaturated carbonyl compounds

With the Mayr electrophilicities *E* for a broad set of α,β-unsaturated carbonyl compounds at hand (from this work), we analyzed the impact of substituents on their electrophilic reactivity in 1,4-additions. The differences in *E* between typical classes of Michael acceptors might be used as increments (expressed as Δ*E*) together with equation 1 to rationalize or estimate the reactivity of Michael acceptors of unknown *E*.

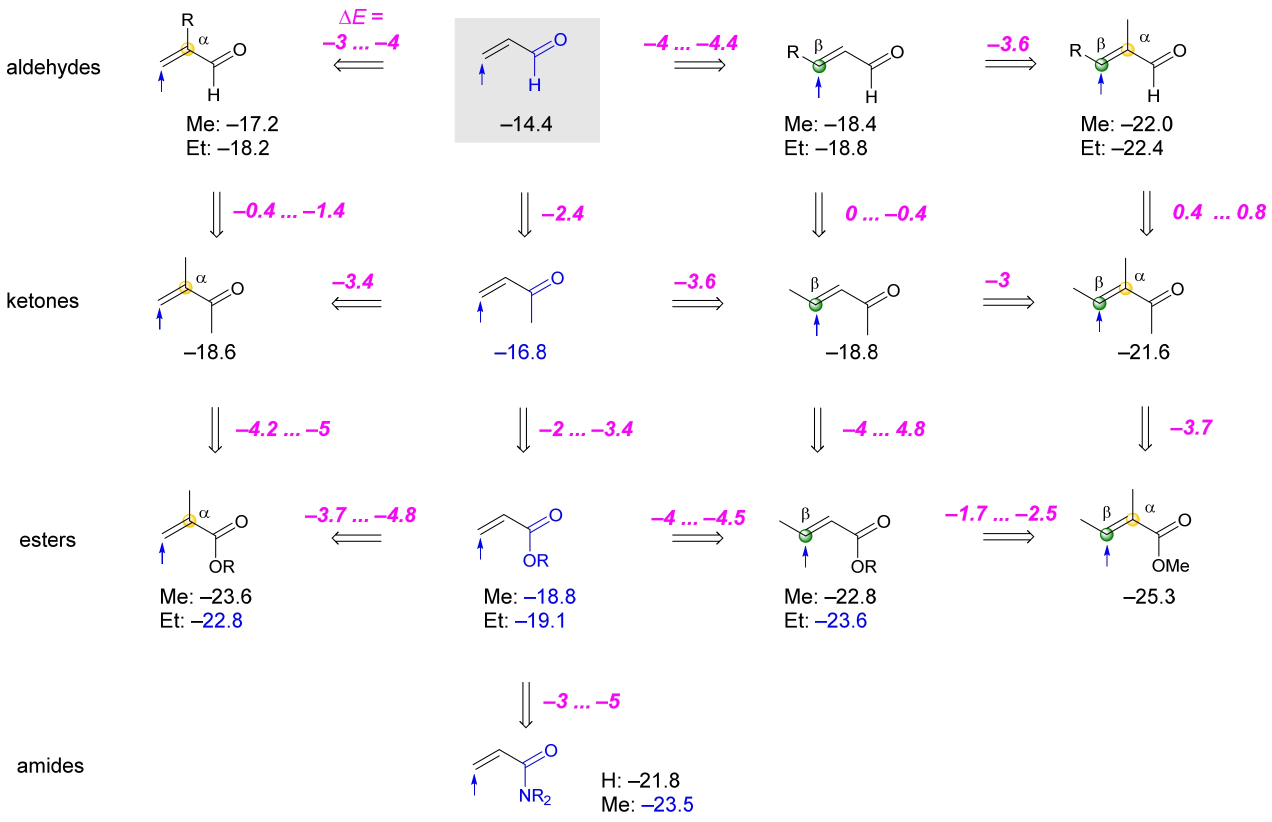

Figure S4. Structure/electrophilicity relationships for Michael acceptors based on the electrophilicities *E* (in blue color: from ref[S10c]; in black color: this work).

Generally, introduction of alkyl substituents in the α-position reduces the electrophilic reactivity of the Michael acceptor on average by 3 to 4 *E* units. Analogously, alkyl substituents in the β-position reduce reactivity by 3.5 to 4.5 *E* units. While the reactivity of α,β-unsaturated aldehydes and the corresponding ketones is in a comparable range (going from an aldehyde to a structurally related ketone generally slightly reduces the reactivity, but the effects are rather small), esters are significantly less reactive by on average of 3 to 5 *E* units than the corresponding ketones. Swapping a ketone for an amide reduces the electrophilicity significantly (Δ*E* = –5 to –8).

Example 4: Rational prediction of Michael additions

According to the Patz-Mayr equation (equation 1, main text), electrophilicity E is defined as a solvent-independent parameter.[S37a] Therefore, the electrophilicity parameters E of Michael acceptors determined in this study can be combined with any known solvent-dependent nucleophilicity parameter N (and s_N) of potential reaction partners[S21] to predict second-order rate constants for their 1,4-addition reactions.[S37b]

Will I get a product within reasonable reaction time? In the majority of the possible electrophile-nucleophile combinations the answer to this most important question for synthetic chemists can be given by calculating the sum lg k_2(20 °C) = $E + N$. If $E + N > –3$, the reaction should be feasible at 20 °C when applying typical reactant concentrations of 0.1 M.

In Figure S5 Michael acceptors and nucleophiles are arranged in a way that $E + N = −3$ for reaction partners on the same horizontal level. Michael acceptors can be expected to react at ambient temperature with nucleophiles on the same level in Figure S5. When equimolar concentrations of 0.1 M are used for the electrophile-nucleophile mixture and s_N is in the typical range (0.5 to 0.8 for nucleophiles with $N > 11$ that undergo Michael additions), a half-reaction time between 1 and 3 hours is expected for a certain Michael acceptor when reacting with nucleophilic reaction partners located at the same horizontal level in Figure S5. A certain Michael acceptor will also react at ambient temperature with those nucleophiles, which are positioned below itself in Figure S5, but only sluggish or not at all with those above its own position. Slower reactions may become feasible under harsher reaction conditions, for example, upon heating or increasing the concentrations of reactants up to neat. This is only a crude approximation, however, and considering the susceptibility parameter s_N in equation 1 is advised for borderline cases.

Please note that the Patz-Mayr equation (equation 1) only analyzes the *kinetic* feasibility for the formation of a σ-bond. Sufficiently *favorable thermodynamics* is another condition for the success of an intended Michael addition. Nevertheless, Figure S5 summarizes well-known synthetically employed reactions of Michael acceptors with nucleophiles. For example, rates of Corey-Chaykovsky cyclopropanations,[S38] stepwise 1,3-dipolar cycloadditions (Huisgen reactions),[S10c] Weitz-Scheffer epoxidations,[S39] cyanoethylations[S20,S22,S24,S26,S31] or simple Michael adduct formations (1,4-addition) of Michael acceptors with amines, alkoxide ions, and thiolates[S20–S31] can be predicted by using equation 1 and the available Mayr reactivity parameters E, N and s_N.[S21] The precision of these predictions can be assessed from the $k_2^{exp}/k_2^{eq\,1}$ ratios, which compare reported experimental rate constants k_2^{exp} with rate constants $k_2^{eq\,1}$ predicted by equation 1 (see Table S6 above).

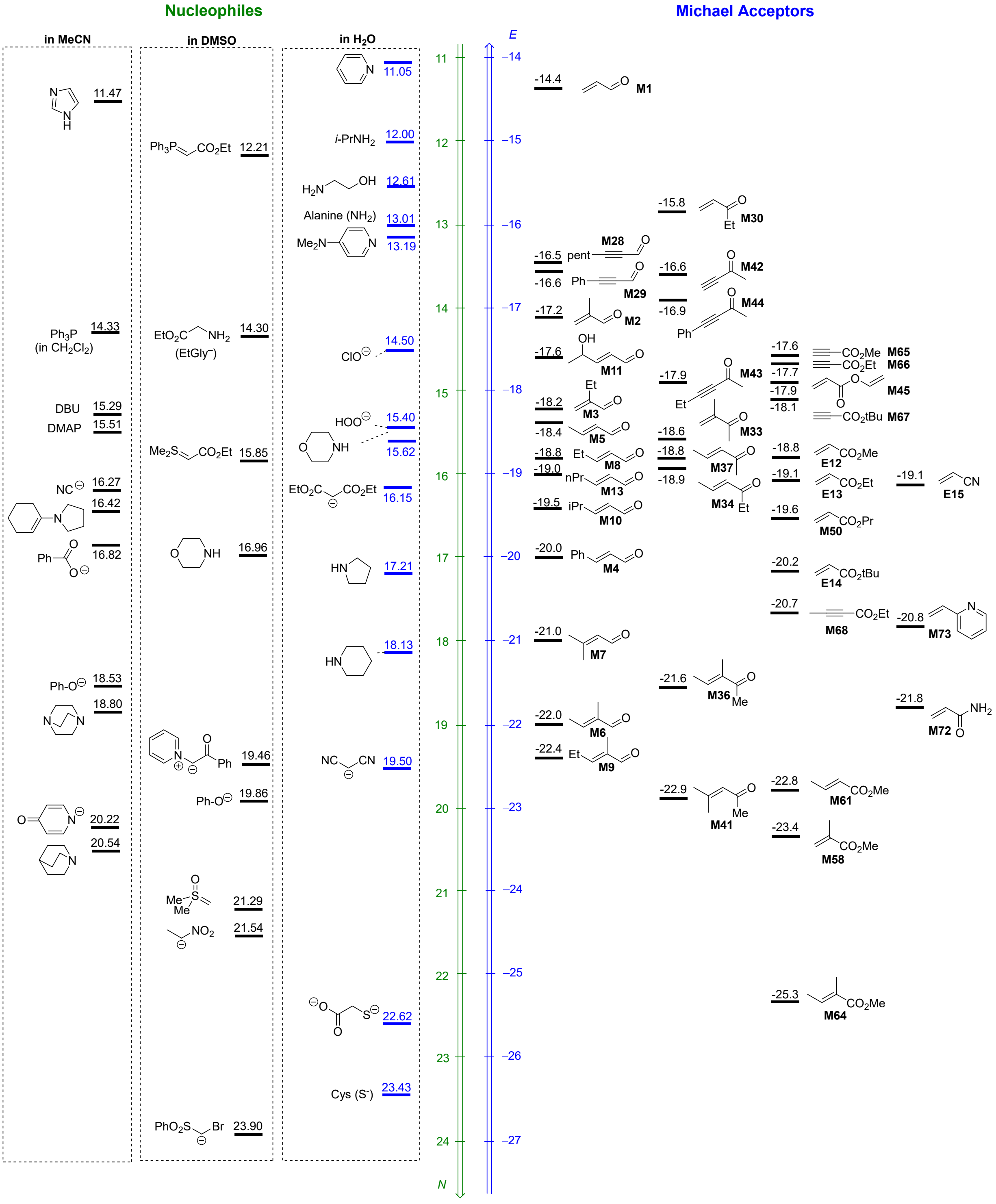

Figure S5. Nucleophilicity (*N*) and electrophilicity (*E*) scales arranged in a way that a certain Michael acceptor will react with those nucleophiles, which are positioned below its own level ($N + E > -3$). Nucleophile-specific parameters *N* were taken from ref[S21], electrophilicity parameters *E* for Michael acceptors were taken from Table S4.

References

[S1] J. T. Edsall, J. Wyman, *Biophysical Chemistry*, Academic Press, New York, 1958.

[S2] M. Friedman, J. F. Cavins, J. S. Wall, *J. Am. Chem. Soc.* **1965**, *87*, 3672–3682.

[S3] D. L. Rabenstein, *J. Am. Chem. Soc.* **1973**, *95*, 2797–2803.

[S4] A. Mirzahosseini, M. Somlyay, B. Noszál, *Chem. Phys. Lett.* **2015**, *622*, 50–56.

[S5] a) T. Bug, H. Mayr, *J. Am. Chem. Soc.* **2003**, *125*, 12980–12986. b) F. Brotzel, H. Mayr, *Org. Biomol. Chem.* **2007**, *5*, 3814–3820.

[S6] A. Meißner, P. Gockel, H. Vahrenkamp, *Chem. Ber.* **1994**, *127*, 1235–1241.

[S7] a) H. Esterbauer, H. Zollner, N. Scholz, *Z. Naturforsch. C* **1975**, *30*, 466–473. b) G. Eisenbrand, J. Schuhmacher, P. Gölzer, *Chem. Res. Toxicol.* **1995**, *8*, 40–46. c) K. Chan, R. Poon, P. J. O'Brien, *J. Appl. Toxicol.* **2008**, *28*, 1027–1039.

[S8] A. Böhme, A. Laqua, G. Schüürmann, *Chem. Res. Toxicol.* **2016**, *29*, 952–962.

[S9] J. A. H. Schwöbel, D. Wondrousch, Y. K. Koleva, J. C. Madden, M. T. D. Cronin, G. Schüürmann, *Chem. Res. Toxicol.* **2010**, *23*, 1576–1585.

[S10] a) H. Mayr, J. Ammer, M. Baidya, B. Maji, T. A. Nigst, A. R. Ofial, T. Singer, *J. Am. Chem. Soc.* **2015**, *137*, 2580–2599; b) P. A. Byrne, S. Kobayashi, E.-U. Würthwein, J. Ammer, H. Mayr, *J. Am. Chem. Soc.* **2017**, *139*, 1499–1511; c) D. S. Allgäuer, H. Jangra, H. Asahara, Z. Li, Q. Chen, H. Zipse, A. R. Ofial, H. Mayr, *J. Am. Chem. Soc.* **2017**, *139*, 13318–13329.

[S11] E. Harder, W. Damm, J. Maple, C. Wu, M. Reboul, J. Y. Xiang, L. Wang, D. Lupyan, M. K. Dahlgren, J. L. Knight, J. W. Kaus, D. S. Cerutti, G. Krilov, W. L. Jorgensen, R. Abel, R. A. Friesner, *J. Chem. Theory Comput.* **2016**, *12*, 281–296.

[S12] *MacroModel* (Schrödinger Release 2019-1), Schrödinger, LLC, New York, NY, 2019.

[S13] A. D. Becke, *J. Chem. Phys.* **1993**, *98*, 5648–5652.

[S14] R. Ditchfield, W. J. Hehre, J. A. Pople, *J. Chem. Phys.* **1971**, *54*, 724–728.

[S15] R. Krishnan, J. S. Binkley, R. Seeger, J. A. Pople, *J. Chem. Phys.* **1980**, *72*, 650–654.

[S16] T. Clark, J. Chandrasekhar, G. W. Spitznagel, P. v. R. Schleyer, *J. Comput. Chem.* **1983**, *4*, 294–301.

[S17] A. V. Marenich, C. J. Cramer, D. G. Truhlar, *J. Phys. Chem. B.* **2009**, *113*, 6378–6396.

[S18] *Gaussian 16, Revision A.03*, M. J. Frisch, G. W. Trucks, H. B. Schlegel, G. E. Scuseria, M. A. Robb, J. R. Cheeseman, G. Scalmani, V. Barone, G. A. Petersson, H. Nakatsuji, X. Li, M. Caricato, A. V. Marenich, J. Bloino, B. G. Janesko, R. Gomperts, B. Mennucci, H. P. Hratchian, J. V. Ortiz, A. F. Izmaylov, J. L. Sonnenberg, D. Williams-Young, F. Ding, F. Lipparini, F. Egidi, J. Goings, B. Peng, A. Petrone, T. Henderson, D. Ranasinghe, V. G. Zakrzewski, J. Gao, N. Rega, G. Zheng, W. Liang, M. Hada, M. Ehara, K. Toyota, R. Fukuda, J. Hasegawa, M. Ishida, T. Nakajima, Y. Honda, O. Kitao, H. Nakai, T. Vreven, K. Throssell, J. A. Montgomery, Jr., J. E. Peralta, F. Ogliaro, M. J. Bearpark, J. J. Heyd, E. N. Brothers, K. N. Kudin, V. N. Staroverov, T. A. Keith, R. Kobayashi, J. Normand, K. Raghavachari, A. P. Rendell, J. C. Burant, S. S. Iyengar, J. Tomasi, M. Cossi, J. M. Millam, M. Klene, C. Adamo, R. Cammi, J. W. Ochterski, R. L. Martin, K. Morokuma, O. Farkas, J. B. Foresman, and D. J. Fox, Gaussian, Inc., Wallingford CT, 2016.

[S19] H. Mayr, *Angew. Chem.* **2011**, *123*, 3692–3698; *Angew. Chem. Int. Ed.* **2011**, *50*, 3612–3618.

[S20] C. K. M. Heo, J. W. Bunting, *J. Org. Chem.* **1992**, *57*, 3570–3578.

[S21] A database of reactivity parameters *E*, *N*, and s_N can be accessed at: www.cup.lmu.de/oc/mayr/DBintro.html.

[S22] H. Shenhav, Z. Rappoport, S. Patai, *J. Chem. Soc. B* **1970**, 469–476.

[S23] H. Esterbauer, *Monatsh. Chem.* **1970**, *101*, 782–810.

[S24] M. Friedman, J. S. Wall, *J. Org. Chem.* **1966**, *31*, 2888–2894.

[S25] N. Ferry, F. J. McQuillin, *J. Chem. Soc.* **1962**, 103–113.

[S26] R. N. Ring, G. C. Tesoro, D. R. Moore, *J. Org. Chem.* **1967**, *32*, 1091–1094.

[S27] I.-H. Um, E.-J. Lee, J.-S. Min, *Tetrahedron* **2001**, *57*, 9585–9589.

[S28] I.-H. Um, J.-S. Lee, S.-M. Yuk, *J. Org. Chem*. **1998**, *63*, 9152–9153.

[S29] I.-H. Um, J.-S. Lee, S.-M. Yuk, *Bull. Korean Chem. Soc*. **1998**, *19*, 776–779.

[S30] S.-I Kim, H.-W. Baek, I.-H. Um, *Bull. Korean Chem. Soc*. **2009**, *30*, 2909–2912.

[S31] B.-A. Feit, A. Zilkha, *J. Org. Chem.* **1963**, *28*, 406–410.

[S32] X. Jiang, J. Chen, A. Bajić, C. Zhang, X. Song, S. L. Carroll, Z.-L. Cai, M. Tang, M. Xue, N. Cheng, C. P. Schaaf, F. Li, K. R. MacKenzie, A. C. M. Ferreon, F. Xia, M. C. Wang, M. Maletić-Savatić, J. Wang, *Nat. Commun*. **2017**, *8*, 16087.

[S33] K. Umezawa, M. Yoshida, M. Kamiya, T. Yamasoba, Y. Urano, *Nat. Chem*. **2017**, *9*, 279–286.

[S34] X. Jiang, Y. Yu, J. Chen, M. Zhao, H. Chen, X. Song, A. J. Matzuk, S. L. Carroll, X. Tan, A. Sizovs, N. Cheng, M. C. Wang, J. Wang, *ACS Chem. Biol.* **2015**, *10*, 864–874.

[S35] J. Chen, X. Jiang, S. L. Carroll, J. Huang, J. Wang, *Org. Lett*. **2015**, *17*, 5978–5981.

[S36] O. García-Beltrán, C. González, E. G. Pérez, B. K. Cassels, J. G. Santos, D. Millán, N. Mena, P. Pavez, M. E. Aliaga, *J. Phys. Org. Chem*. **2012**, *25*, 946–952.

[S37] a) H. Mayr, M. Patz, *Angew. Chem.* **1994**, *106*, 990-1010; *Angew. Chem. Int. Ed. Engl.* **1994**, *33*, 938–957. b) H. Mayr, A. R. Ofial, *Pure Appl. Chem.* **2017**, *89*, 729-744.

[S38] R. Appel, N. Hartmann, H. Mayr, *J. Am. Chem. Soc.* **2010**, *132*, 17894–17900.

[S39] a) R. J. Mayer, T. Tokuyasu, P. Mayer, J. Gomar, S. Sabelle, B. Mennucci, H. Mayr, A. R. Ofial, *Angew. Chem.* **2017**, *129*, 13463–13467; *Angew. Chem. Int. Ed.* **2017**, *56*, 13279–13282. b) R. J. Mayer, A. R. Ofial, *Org. Lett.* **2018**, *20*, 2816–2820. c) R. J. Mayer, A. R. Ofial, *Eur. J. Org. Chem.* **2018**, 6010–6017.
